# Scientific production in the era of large language models: Outcome-triggered treatment timing and spurious event-study dynamics

**DOI:** 10.1073/pnas.2618638123

**Published:** 2026-08-11

**Authors:** Thomas Renault, Antonin Bergeaud, Clément Bosquet

**Affiliations:** ^a^ Université Paris-Saclay, Faculté Jean Monnet, Sceaux 92330, France; ^b^ HEC Paris, Jouy-en-Josas 77850, France; ^c^ Université Paris 1 Panthéon-Sorbonne, Centre d’Economie de la Sorbonne, Paris 75013, France

**Keywords:** large language models, scientific productivity, event studies, treatment timing, causal inference

## Abstract

Large language models (LLMs) are increasingly used in scientific writing, but their effect on individual productivity is difficult to identify because adoption is rarely directly observed. [K. Kusumegi *et al.*, *Science* **390**, 1240–1243 (2025)] infer adoption from the first paper detected as LLM-assisted and report large productivity gains after adoption. We show that this treatment-timing rule mechanically generates positive event-study dynamics even in the absence of any causal effect. Because high-output months are more likely to produce a detected paper, treatment assignment becomes intrinsically linked to productivity. Using reconstructed arXiv data, we show that random treatment assignments, neutral-keyword triggers, inverted treatment, and pre-ChatGPT placebo periods all generate similar dynamics. Simulations with no treatment effect also reproduce the same posttreatment patterns reported in K. Kusumegi *et al.*, *Science* **390**, 1240–1243 (2025). Our results demonstrate that first-detection timing alone creates spurious evidence of productivity gains from LLM adoption.

Large language models (LLMs) are rapidly changing how scientists search for information, write manuscripts, and revise research outputs. An important question is whether these tools increase scientific productivity, and if so by how much. At the aggregate level, recent evidence documents a sharp increase in scientific submissions and working-paper production following the diffusion of generative AI tools (1, 2). Measuring the effect at the individual researcher level, however, is substantially more difficult because adoption is rarely observed directly and must instead be inferred from textual or behavioral traces.

A prominent recent study addresses this challenge by constructing an author-level measure of LLM adoption from scientific abstracts (3). The authors train a detector designed to identify LLM-assisted writing and classify a researcher as treated in the first month after the release of ChatGPT (December 2022) in which at least one submitted abstract is flagged as AI-assisted. Using this treatment definition in a stacked event-study design, they find that “the number of arXiv preprints published monthly once an author had adopted LLMs in their writing increased by 36.2% relative to nonadopters.”

We use this empirical setting to study a broader identification problem in observational research: treatment timing determined by the first occurrence of a detected event in an output-generating process. More specifically, we ask whether positive event-study dynamics can arise mechanically when treatment is assigned at the first observed detection, rather than from directly observed adoption behavior.

We address this question in three steps. First, we reconstruct the empirical strategy of ref. 3 using raw arXiv metadata and augment the analysis with several placebo exercises designed to preserve the treatment-timing structure while removing any information about true LLM adoption. Across different settings, we recover positive posttreatment patterns that are similar in both shape and magnitude to those reported in the original study. Second, we study the mechanism in simulations with independently distributed productivity and no causal treatment effect. Third, we formalize the statistical properties of first-detection timing and characterize the event-study dynamics implied by the design. Together, the results show that event-study dynamics generated by first-detection timing cannot, by themselves, be interpreted as evidence of productivity gains from LLM adoption.

## Results

The key feature of the design in ref. 3 is that treatment timing is defined by the first month in which a researcher has at least one paper classified as LLM-assisted. Because the probability of observing a flagged paper increases with the number of papers submitted in a month, treatment assignment is mechanically more likely to occur during periods of high output. In addition, pretreatment periods are selected under the condition that no earlier detection occurred, whereas posttreatment periods are not subject to the same restriction. These two features jointly alter the composition of observations around the treatment date and can generate positive event-study dynamics even when there is no causal effect of LLM adoption.

**Fig. 1.**
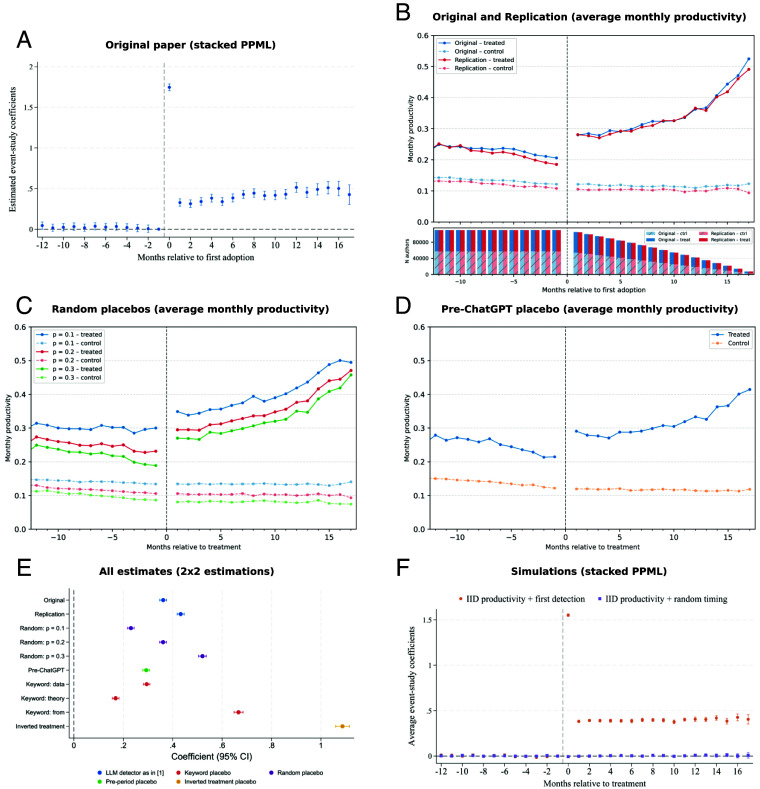
Placebo tests and simulations show spurious evidence of productivity gains from LLM adoption. (*A*) Reproduction of the stacked PPML event study from the replication package of Kusumegi et al., with the treatment-month coefficient k = 0 restored. (*B*) Average monthly productivity, measured as the number of arXiv articles per author, before and after the inferred treatment date, comparing the original analysis with our reconstruction from raw arXiv metadata (excluding k = 0). (*C*) Random placebo in which post-ChatGPT papers are independently flagged with fixed probability. (*D*) Pre-ChatGPT placebo in which the entire analysis window is shifted to January 2020–June 2022, before the release of ChatGPT. (*E*) Average treatment effect estimated from stacked 2×2 PPML regressions across all placebo specifications. (*F*) Average stacked-PPML event-study coefficients across 100 simulations with 10,000 authors and i.i.d. productivity (flag probability *P* = 0.3 in the first-detection scenario).

We first examined this mechanism directly using the original replication package of ref. 3. We reran the stacked Poisson event-study specification and restored the first treatment-month coefficient (k = 0), which was omitted from the published figure. The resulting event study shows a sharp increase exactly at the treatment month, consistent with the prediction of the first-detection rule: Researchers are most likely to enter treatment in months with unusually high submission activity (Fig. 1*A*).

We then reconstructed the arXiv component of the empirical strategy from raw metadata following (3) and applied a mixture-style detector to post-ChatGPT abstracts (as in ref. 4, see *Materials and Methods*). The reconstructed sample closely matches the original dataset (*SI Appendix*, section 1). Authors in the treated group (those inferred to use LLMs) appear to exhibit a large increase in productivity, whereas authors in the control group show no corresponding change (Fig. 1*B*; see *SI Appendix*, section 2 for the coefficients from the stacked Poisson Pseudo Maximum Likelihood (PPML regression). However, this apparent increase is an artifact of the treatment-timing identification.

To demonstrate this, we replaced the LLM detector with four placebo treatment clocks that preserve the same first-detection timing structure. In the random placebo, each post-ChatGPT paper is independently flagged with probability *P*. In the pre-ChatGPT placebo, the entire design is shifted to the January 2020 to June 2022 period, before ChatGPT existed. In the keyword placebo, papers are flagged when abstracts contain neutral words such as “*theory*,” “*data*,” or “*from*,” which have a similar probability of being written by a human or an LLM according to the mixture-style detector. In the inverted-treatment placebo, we reverse the treatment definition by classifying as LLM-written papers with a mixture-style detector score below 0.1, instead of above the 0.1 threshold used in the original paper. All four placebo procedures generate positive posttreatment event-study paths, with effect sizes that bracket the estimate reported in the original paper (Fig. 1 *C*–*E*; see also *SI Appendix*, section 3). The empirical signature attributed to LLM adoption in ref. 3 therefore also emerges when treatment timing is generated by rules that cannot measure true LLM adoption.^*^

Last, we conducted simulations in which productivity is independently distributed over time and unaffected by treatment. Under random treatment timing, estimated coefficients remain centered near zero. Under first-detection timing, however, the estimator produces a large spike at k = 0 followed by persistently positive posttreatment coefficients (Fig. 1*F*; closely matching both the pattern and the magnitude shown in Fig. 1*A*). We also derive the corresponding analytical expressions (*SI Appendix*, section 4) and show that the combination of a treatment-month jump and a lower positive posttreatment plateau is the theoretical prediction of the design even in the absence of any true LLM effect. Importantly, the estimated effect is a function of the probability of flagging a paper as LLM-written. This explains the patterns we observe when replicating the stacked event-study approach with our placebo analyses: The estimated effects become larger as the treatment probability (*P*) increases, for keywords that are more frequent (such as “from”) and when the treatment definition is inverted, because fewer than 50% of papers are classified as LLM-written under the original definition, so inverting the treatment increases the probability of treatment assignment.

## Discussion

Our results show that the treatment definition in ref. 3 mechanically links treatment timing to publication output. This concern is distinct from the standard endogeneity of technology adoption, a limitation highlighted in ref. 3. Even if one abstracts from nonrandom adoption decisions, the design itself creates an asymmetric comparison between pre- and posttreatment periods. The omitted pretreatment reference period is conditioned on the absence of earlier detections, whereas posttreatment months are not. Our analysis shows that this asymmetry alone is sufficient to generate the characteristic event-study pattern observed in the data: a sharp increase at treatment followed by persistently positive posttreatment coefficients. This means that the event-study pattern documented in ref. 3 is not diagnostic of productivity gains, even if the original estimates are read purely descriptively rather than causally.

These findings do not imply that LLMs have no effect on scientific productivity. Rather, they show that the empirical strategy in ref. 3 cannot distinguish the effect of LLM adoption from the mechanical bias induced by defining treatment based on the first detected LLM-generated paper. The LLM-detector robustness checks proposed in ref. 3, such as using alternative detection thresholds or GPTZero, do not address this issue: Any detector used to define first adoption from the first detected output creates the same timing problem and will only change the magnitude of the effect mechanically based on the probability of detection. The problem we document applies to all analyses in ref. 3 that examine productivity changes following LLM adoption. We do not make broader claims about the validity of the other empirical analyses presented in ref. 3, which we have not systematically investigated.

More broadly, our findings highlight a general identification problem in observational studies where treatment timing is inferred from the first occurrence of an event embedded in the outcome process itself. The issue is therefore not specific to LLM detection or to scientific productivity. More reliable identification of AI effects will require research designs in which adoption timing and intensity are directly observed rather than inferred from realized output. Future work should therefore prioritize targeted data collection on actual AI usage and exposure, ideally through prospective study designs or collaborations with organizations able to provide independent measures of AI adoption (see ref. 5 for recent survey evidence on AI and coding-agent use among social scientists). When direct measures of adoption are unavailable, an alternative is to exploit plausibly exogenous sources of variation in AI exposure, such as differential access to AI tools, staggered rollouts, institutional constraints, or pretreatment differences in potential exposure.

## Materials and Methods

We use arXiv metadata from the Cornell/arXiv Kaggle snapshot covering January 2016 to June 2024. Following the sample construction of Kusumegi et al. (3), we exclude papers from core AI fields, including computer vision, machine learning, artificial intelligence, information retrieval, and computational linguistics. To approximate the classification procedure used in ref. 3, and following (4), we implement a mixture-style detector trained on 20,000 synthetic abstract pairs composed of original abstracts and GPT-3.5 rewrites. Details on the placebo tests, the stacked difference-in-differences design, and the simulation design are provided in *SI Appendix* and the replication repository.

## Supplementary Material

Appendix 01 (PDF)

## Data Availability

Code and raw data have been deposited in GitHub (https://github.com/trenault/llm_productivity_comment (6)).
