# Supplementary material for: Scientific production in the era of large language models: Outcome-triggered treatment timing and spurious event-study dynamics
Source: Proc Natl Acad Sci U S A. 2026 Aug 11;123(33):e2618638123. doi: 10.1073/pnas.2618638123 (PMC13486544; doi:10.1073/pnas.2618638123)
Supplement: Supplementary file 1 [file pnas.2618638123.sapp.pdf]

Online appendix to
"Scientific Production in the Era of Large Language Models: Outcome-Triggered Treatment Timing and Spurious Event-Study Dynamics"

Thomas Renault* Antonin Bergeaud† Clément Bosquet‡

July 21, 2026

Contents

1 Samples' comparison **3**

2 Figure 1B in stacked-PPML event study **4**

3 Placebo exercises of Figure 1 **5**
3.1 Average monthly productivity . . . 5
3.2 Stacked-PPML event study . . . 6

4 The question and where the bias comes from **7**
4.1 The intuition in a single comparison . . . 7
4.2 The design the paper uses . . . 8
4.2.1 The post-adoption coefficient . . . 8
4.2.2 The stacked estimator . . . 8

5 Placebo exercises of Figure 1 in stacked-OLS event study **10**
5.1 With the dependent variable in levels (y = number of papers) . . . 10
5.2 with $\ln(0.1 + y)$ as dependent variable . . . 11
5.3 with $\ln(0.01 + y)$ as dependent variable . . . 12
5.4 with $\ln(x + \sqrt{x^2 + 1})$ (inverse hyperbolic sine) as dependent variable . . . 13

6 Placebo exercises of Figure 1 with modern event-study estimators for staggered designs **14**
6.1 de Chaisemartin and d'Haultfœuille's (2026) estimator . . . 14
6.1.1 with the dependent variable in levels (y = number of papers) . . . 14
6.1.2 with $\ln(0.1 + y)$ as dependent variable . . . 15
6.1.3 with $\ln(0.01 + y)$ as dependent variable . . . 16
6.1.4 with $\ln(x + \sqrt{x^2 + 1})$ (inverse hyperbolic sine) as dependent variable . . . 17
6.2 Sun and Abraham's (2021) estimator . . . 18
6.2.1 with the dependent variable in levels (y = number of papers) . . . 18
6.2.2 with $\ln(0.1 + y)$ as dependent variable . . . 19

*Université Paris-Saclay, RITM, Faculté DEM, 54 Boulevard Desgranges, 92330 Sceaux, France. Email: *thomas.renault1@universite-paris-saclay.fr*

†HEC Paris, 1 rue de la libération, 78350 Jouy-en-Josas, France. Email : bergeaud@hec.fr

‡Université Paris 1 Panthéon-Sorbonne, CNRS, Centre d'Économie de la Sorbonne, 106-112 bd de l'Hôpital, FR-75642 Paris, France. Email: clement.bosquet@univ-paris1.fr

6.2.3 with $\ln(0.01 + y)$ as dependent variable . . . 20
6.2.4 with $\ln(x + \sqrt{x^2 + 1})$ (inverse hyperbolic sine) as dependent variable . . . 21

7 Random treatment placebo with event-time balanced windows 22

8 Placebo exercises of Figure 1 in stacked-PPML event study with alternative control group timing 23

9 Estimations excluding observations corresponding to the first treatment month 24
9.1 Stacked-PPML event study . . . 24
9.2 Two-by-two DiD estimators (corresponding to Figure 1E) . . . 25

10 Event studies showing the first-treatment-month coefficient ($k = 0$) 26
10.1 Placebo exercises of Figure 1 in stacked-PPML event study . . . 26
10.2 Placebo exercises of Figure 1 in stacked-OLS event study . . . 27
10.2.1 With the dependent variable in levels (y = number of papers) . . . 27
10.2.2 with $\ln(0.1 + y)$ as dependent variable . . . 28
10.2.3 with $\ln(0.01 + y)$ as dependent variable . . . 29
10.2.4 with $\ln(x + \sqrt{x^2 + 1})$ (inverse hyperbolic sine) as dependent variable . . . 30
10.3 Placebo exercises of Figure 1 with modern event-study estimators for staggered designs . . . 31
10.3.1 de Chaisemartin and d'Haultfœuille's (2026) estimator . . . 31
10.3.2 Sun and Abraham's (2021) estimator . . . 35
10.4 Random treatment placebo with event-time balanced windows . . . 39
10.5 Stacked-PPML event study with alternative control group timing . . . 40

11 Materials and Methods: Additional Details 41

1 Samples' comparison

Table S1 compares key sample statistics between the original Kusumegi et al. [1] dataset (`production_arxiv.csv`) and our reconstructed replication dataset. Table S2 reports the distribution of treated authors across cohorts (month of first LLM adoption) in both datasets.

Table S1: **Sample comparison**: original vs. replication dataset

	Original paper	Replication
Authors (N)	109,965	109,511
— treated	52,628	52,383
— control	57,337	57,128
Treated share (%)	47.9	47.8
Cohorts (N)	18	18
Cohort range (month index)	13–30	13–30
Event-time support	-29 to +17	-29 to +17
Obs. (author-months)	3,298,950	3,285,330
Total papers (post-Dec 22)	407,569	393,267
Mean monthly output (all)	0.2031	0.1981
— treated, pre	0.2217	0.2150
— treated, post	0.3843	0.4026
— control, pre	0.1387	0.1289
— control, post	0.1176	0.1040
Mean event-time — treated	20.14	20.25
Mean event-time — control	21.49	21.50

Table S2: **Cohort distribution** of treated authors (% of treated total)

Cohort (month index)	N (original)	Original (%)	N (replication)	Replication (%)
13	4040	7.7	4087	7.8
14	3770	7.2	3805	7.3
15	4284	8.1	4030	7.7
16	3402	6.5	3296	6.3
17	3990	7.6	3941	7.5
18	3784	7.2	3540	6.8
19	3145	6	3178	6.1
20	2839	5.4	2757	5.3
21	2935	5.6	3049	5.8
22	3103	5.9	2877	5.5
23	2914	5.5	2745	5.2
24	2487	4.7	2566	4.9
25	2201	4.2	2282	4.4
26	2103	4	2187	4.2
27	2134	4.1	2178	4.2
28	1840	3.5	1961	3.7
29	1961	3.7	2016	3.8
30	1696	3.2	1888	3.6

2 Figure 1B in stacked-PPML event study

Figure S1 reports stacked-PPML event-study coefficients with our replication of Kusumegi et al. [1], with and without the first-treatment-month coefficient ($k = 0$).

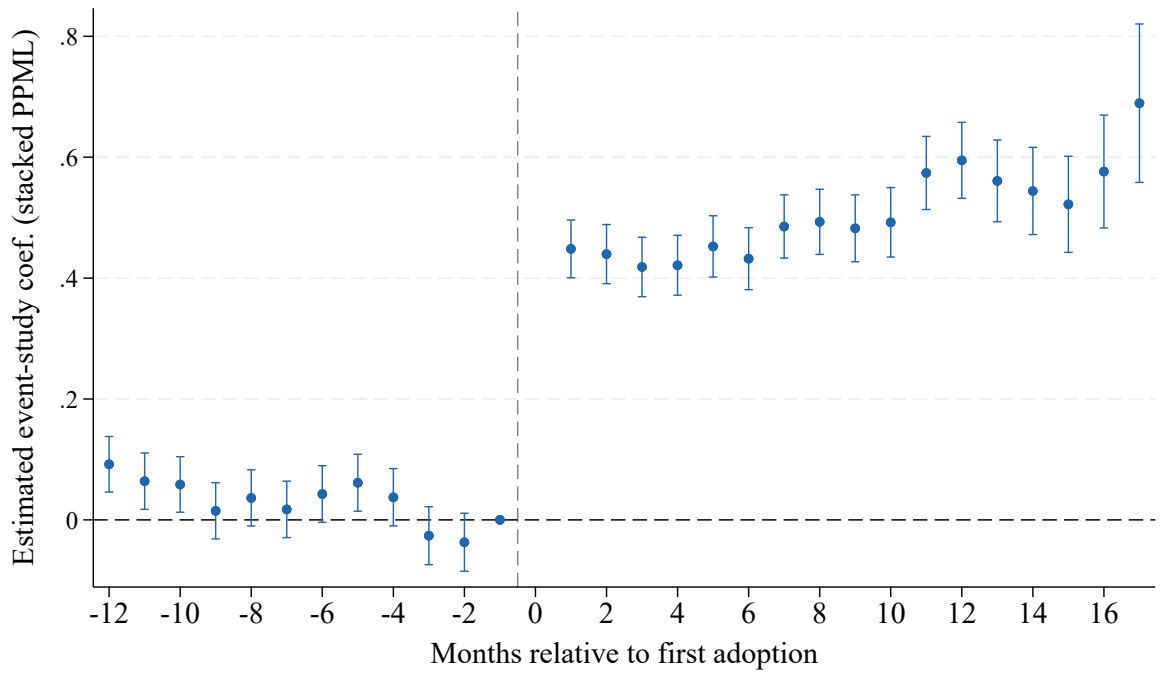

(A) not showing the coefficient for $k = 0$

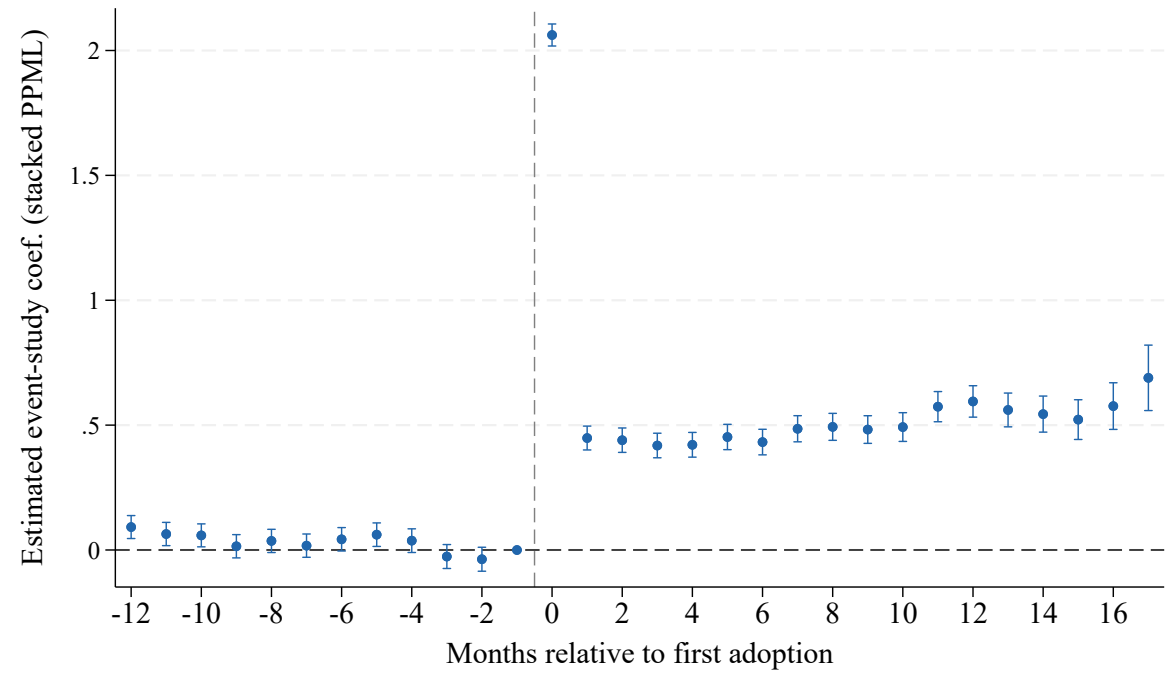

(B) showing the coefficient for $k = 0$

Figure S1: Stacked-PPML event study, **replication**

3 Placebo exercises of Figure 1

3.1 Average monthly productivity

Figure S2 reports average monthly productivity for treated and control authors around the treatment date under each placebo specification.

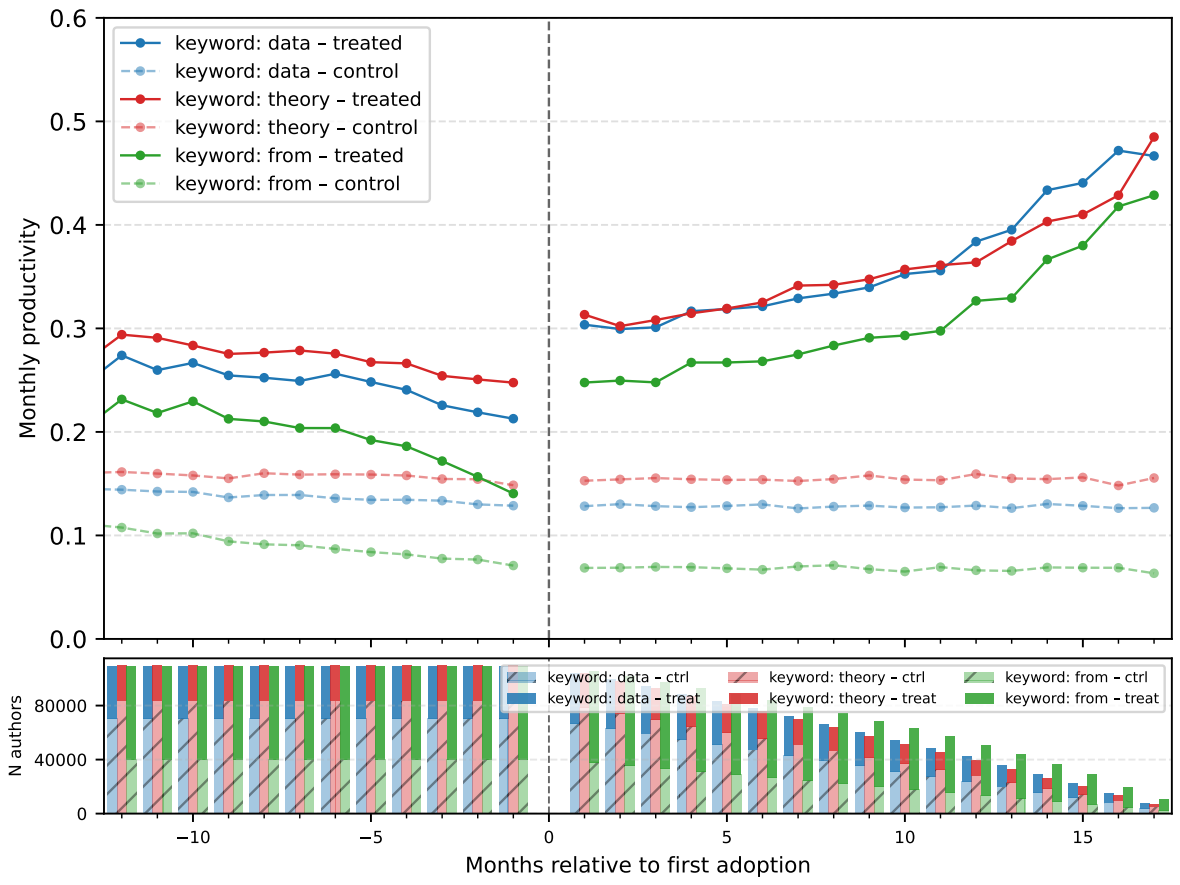

(A) **Placebo 2: keyword treatment**

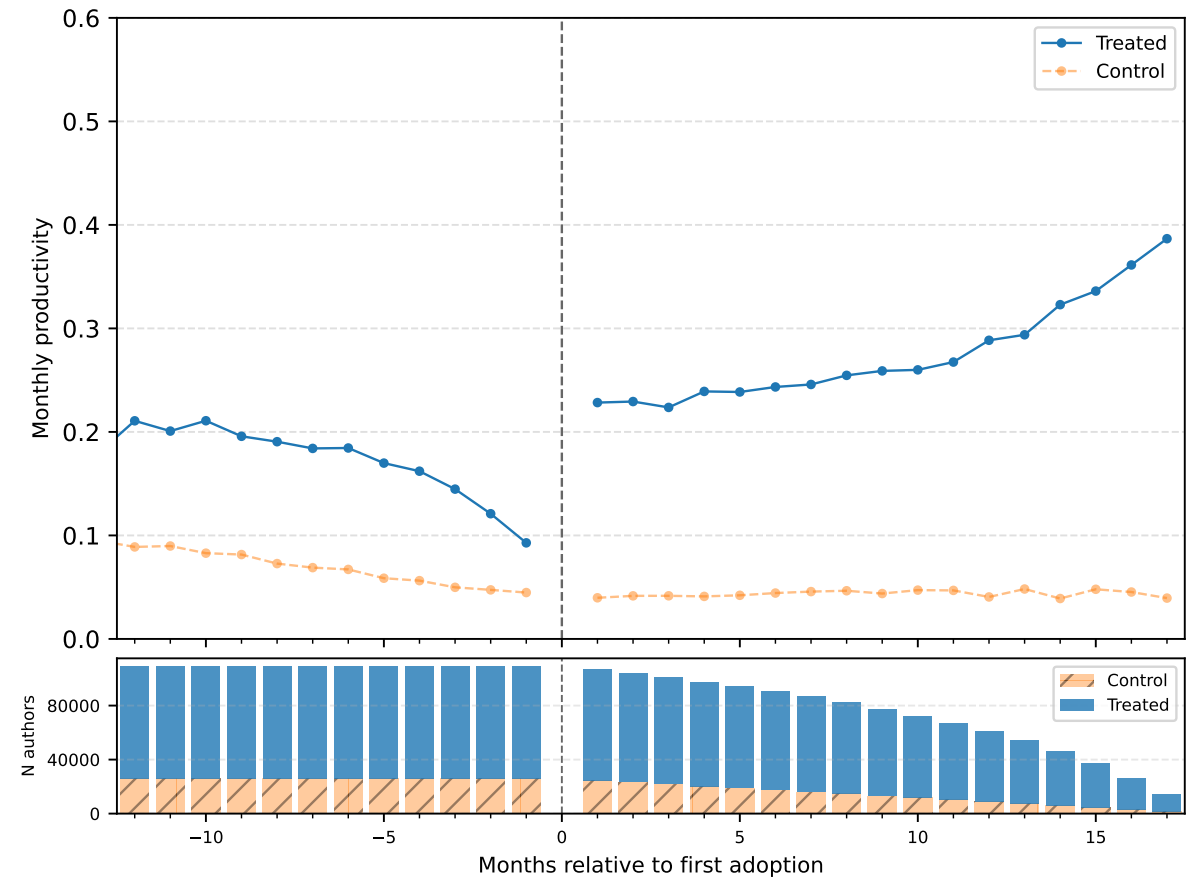

(B) **Placebo 4: inverted treatment**

Figure S2: **Placebo studies.** Both panels report average monthly productivity for placebo exercises that replace the LLM detector with neutral keyword flags (A), and inverted treatment using $\alpha < 0.1$ instead of $\alpha > 0.1$ (B).

3.2 Stacked-PPML event study

Figure S3 reports stacked-PPML event-study coefficients, with 95% confidence intervals, for each placebo specification. Coefficients are shown excluding the first treatment month ($k = 0$), which is mechanically positive when treatment is defined by the first occurrence of the outcome. Figure S20 in section 10.1 shows these coefficients.

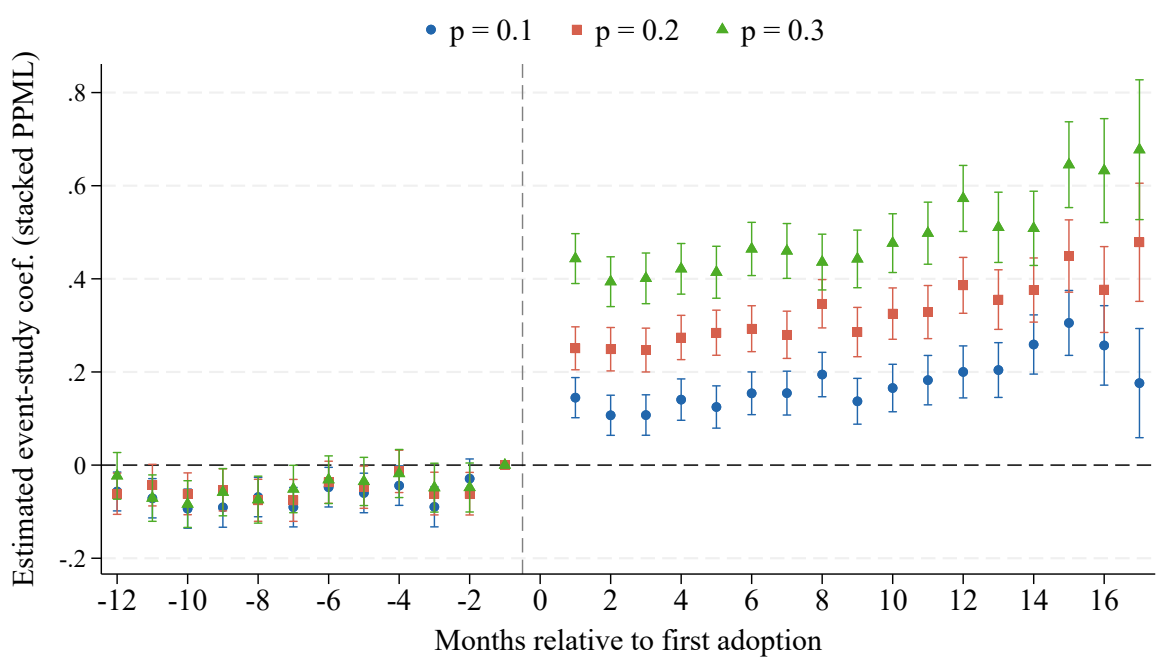

(A) **Placebo 1: random treatment**

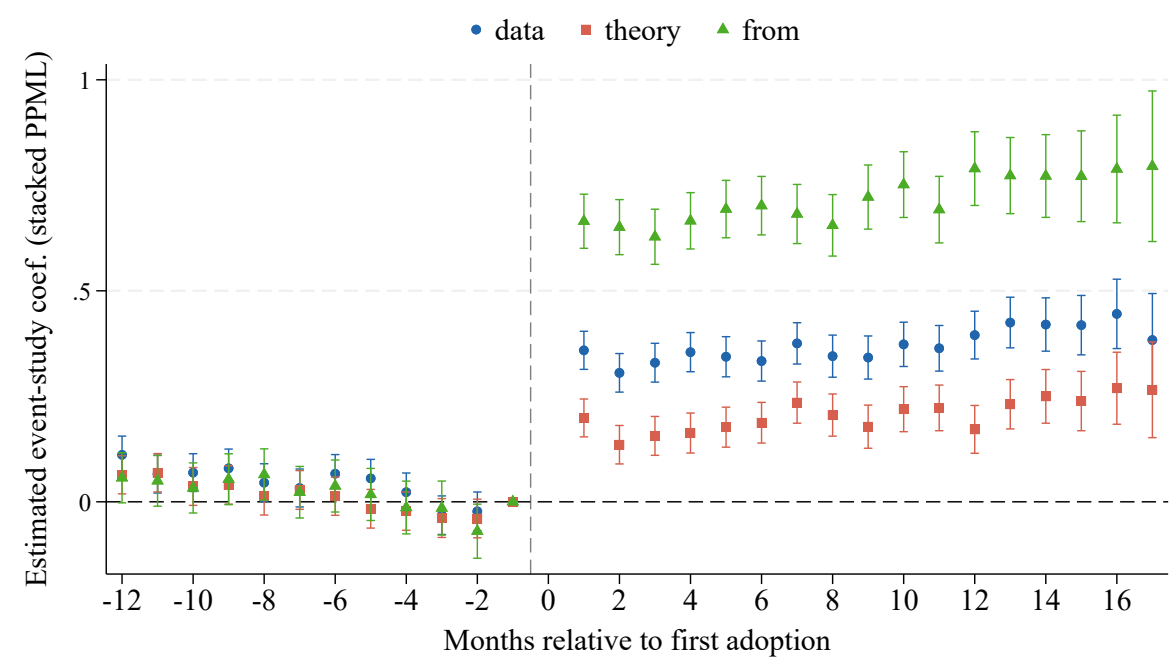

(B) **Placebo 2: keyword treatment**

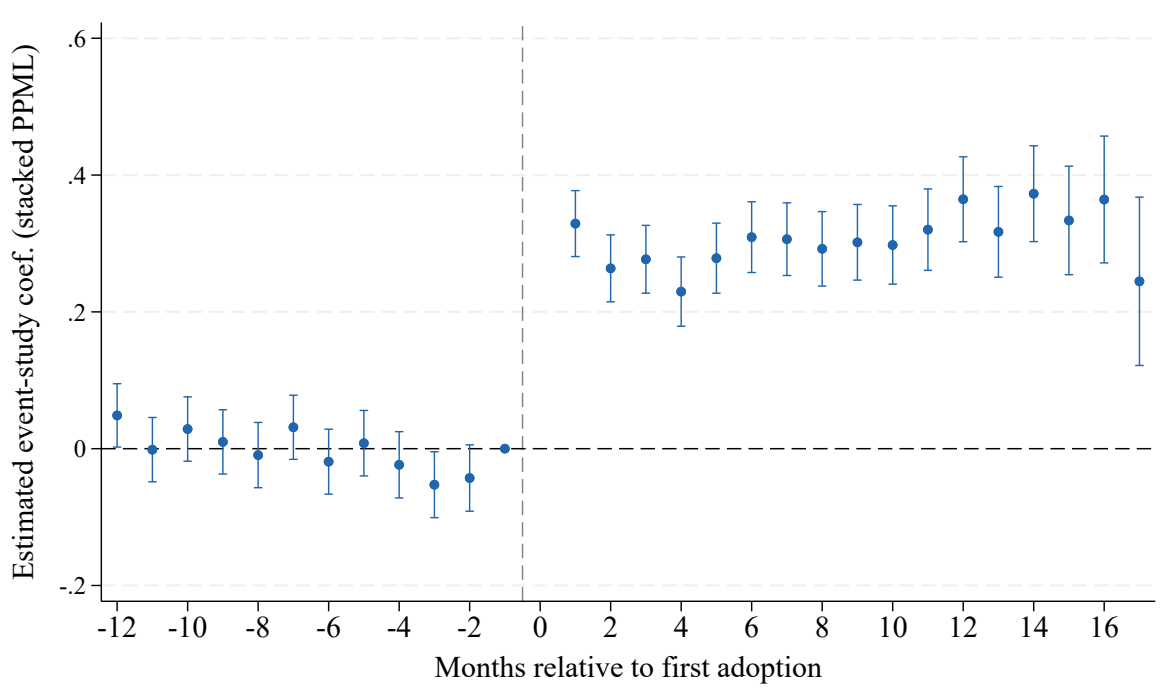

(C) **Placebo 3: pre-ChatGPT period**

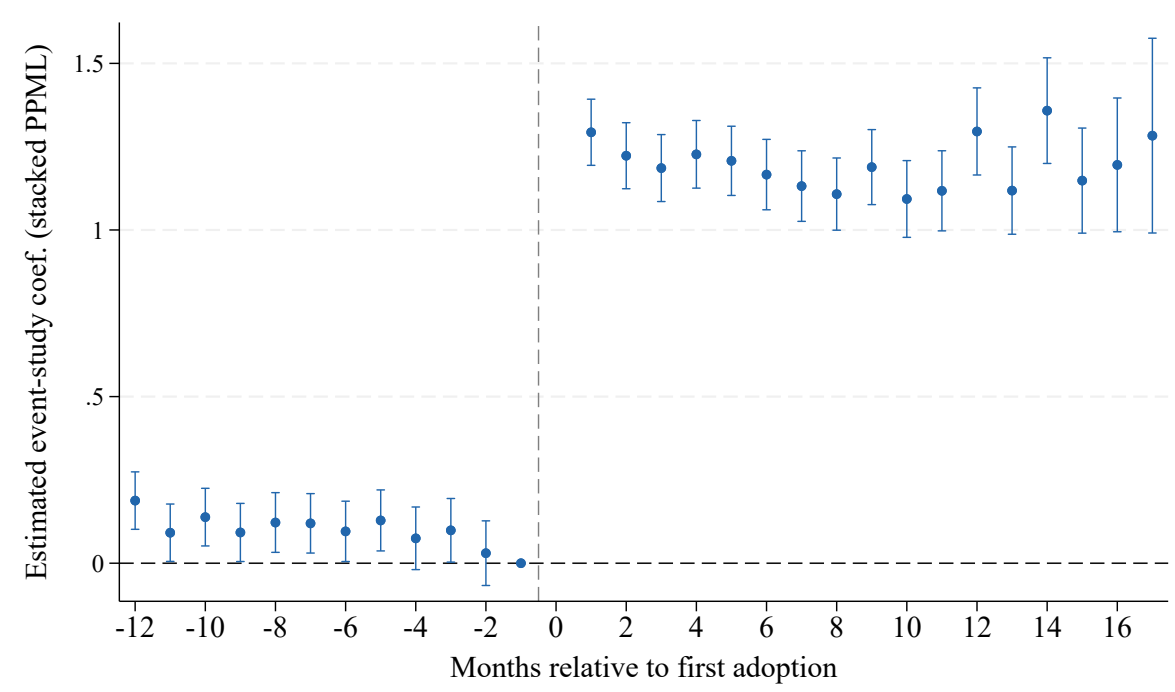

(D) **Placebo 4: inverted treatment**

Figure S3: **Placebo event studies.** All panels report stacked-PPML event-study coefficients, with 95% confidence intervals, of placebo exercises that replace the LLM detector with random paper-level flags (A), neutral keyword flags (B), an earlier observation window (C), and inverted treatment using $\alpha < 0.1$ instead of $\alpha > 0.1$ (D).

4 The question and where the bias comes from

Kusumegi et al. [1] ask whether adopting a large language model raises a researcher's output, measured by the number of preprints that author i posts on arXiv in month t, which we write $Y_{i,t}$. The difficulty is that adoption is never observed directly. The design infers it from text: an LLM detector is run on every abstract, and an author is treated from the first month in which one of their abstracts is flagged as LLM-assisted.

Two objects make this precise. Let $F_{i,t}$ be the number of author i's month-t abstracts that the detector flags, and let

$$H_{i,t} = \mathbb{1}(F_{i,t} > 0)$$

indicate a month with at least one flagged abstract. The detector can only flag abstracts written after the public release of ChatGPT in December 2022, which we denote T_0; earlier abstracts are used to learn what human writing looks like and are never flagged. An author's adoption date is the first flagged month,

$$G_i = \min\{\, t > T_0 : \; H_{i,t} = 1 \,\},$$

and $A_i = \mathbb{1}(G_i < \infty)$ marks the authors who are flagged at some point. The study compares these authors with those who are never flagged ($A_i = 0$).

We ask what this design recovers when the technology does nothing. The null hypothesis is that LLM use has no causal effect on output: each author would post the same preprints whether or not they used an LLM, $Y_{i,t}(1) = Y_{i,t}(0)$. To expose the mechanical force at work, we add one modeling assumption about the detector under this null: each submitted abstract is flagged independently with probability $p \in (0, 1)$. It is worth being clear about what p is. It is a property of the *detector*, not of true adoption: it mixes the detector's true-positive and false-positive rates. Nothing below uses the meaning of a flag; what matters is only that flagging happens paper by paper, so that a month containing y abstracts avoids a flag with probability $(1 - p)^y$ and contains at least one with probability

$$\mathbb{P}(H_{i,t} = 1 \mid Y_{i,t} = y) = 1 - (1 - p)^y,$$

which rises with y. A productive month is therefore more likely to be flagged. This single fact is the whole problem: the first flagged month is selected to be an unusually *high*-output month, while every month before it is selected to contain no flag and is therefore an unusually *low*-output month. An event study built around the first flag reads this selection as if it were a treatment effect.

4.1 The intuition in a single comparison

To see the selection cleanly, begin with the simplest possible case: one adoption cohort and two groups of authors (those eventually flagged and those never flagged) with output and flags independent across months. Let $\mu = \mathbb{E}[Y]$ be ordinary monthly output, and define the average output in the two kinds of month that the design conditions on:

$$m^- \equiv \mathbb{E}[Y \mid H = 0] = \frac{\mathbb{E}\big[Y(1-p)^Y\big]}{\mathbb{E}[(1-p)^Y]}, \qquad m^+ \equiv \mathbb{E}[Y \mid H = 1] = \frac{\mathbb{E}\big[Y\big(1 - (1-p)^Y\big)\big]}{\mathbb{E}[1 - (1-p)^Y]}.$$

It is straightforward to show that $m^- < \mu < m^+$.

Now follow an author through event time $k = t - G_i$, the number of months since the first flag. Before the first flag every month is, by definition, a no-flag month, with expected output m^-. The first-flag month contains a flag, with expected output m^+. After the first flag the event definition imposes no restriction, so expected output returns to μ. A never-flagged author is in a no-flag month throughout, with expected output m^- in every period. The linear event study contrasts each group's change from the reference month $k = -1$, which gives

$$\delta_k = \begin{cases} 0, & k \le -2, \\ m^+ - m^-, & k = 0, \\ \mu - m^-, & k \ge 1. \end{cases}$$

The leads are flat, there is a one-month spike when the flag occurs, and the post-adoption coefficients form a positive plateau $\mu - m^- > 0$.

In practice, the empirical specification is not linear but a Poisson regression with a log link, so the same contrast is taken in logs and the plateau becomes $\log \mu - \log m^-$. When output is Poisson with mean μ, the no-flag mean has a simple closed form, $m^- = \mu(1-p)$, and the post-adoption plateau is

$$\delta_k = \log \mu - \log\big(\mu(1-p)\big) = -\log(1-p) > 0, \qquad k \geq 1,$$

a proportional increase of $\exp(\delta_k) - 1 = p/(1-p)$ that depends only on the flag rate p and on nothing about productivity. The Poisson assumption delivers this closed-form solution but the positive *sign* needs only $m^- < \mu$, which holds for any nondegenerate output distribution.

4.2 The design the paper uses

The single comparison above conveys the intuition, but the actual estimator used in the paper is more complex and uses a stacked design with fixed effects.

4.2.1 The post-adoption coefficient

For clarity we present the result with a single post-adoption coefficient rather than the full event-time path. In place of the dynamic coefficients δ_k, we use the static specification

$$\mathbb{E}[Y_{i,t} \mid \alpha_i, \lambda_{C_i,t}, A_i] = \exp(\alpha_i + \lambda_{C_i,t} + \delta\, A_i\, \text{Post}_{i,t}), \qquad t \neq G_i,$$

where $\text{Post}_{i,t} = \mathbb{1}(t > G_i)$ and the adoption month $t = G_i$ is dropped. Nothing in the argument turns on this choice, for three reasons. First, the post-adoption dynamics presented in the paper (in the first figure) are essentially flat: in the simple comparison above every month after adoption carries the same value $-\log(1-p)$, and the authors' own event study shows the same positive plateau. Second, the adoption month is special as it is selected to contain a flag and it has the size-biased mean $m^+ > \mu$, as we confirm below and the authors' post-adoption claim does not rest on it. Third, the identical selection argument can be run coefficient by coefficient in the dynamic specification, where it returns $-\log(1-p)$ at every $k \geq 1$.

4.2.2 The stacked estimator

The estimator *stacks* the data: each cohort of authors first flagged in the same month is paired with never-flagged authors, who are assigned a pseudo-adoption month so that they sit somewhere on the same event-time axis. The regression then includes author fixed effects α_i and a separate calendar path for each cohort, the cohort-by-month fixed effects $\lambda_{C_i,t}$, where $C_i = G_i$ for flagged authors and C_i is the randomized pseudo-month for the rest. Stacking is designed to avoid the contaminated comparisons across already-treated units that distort ordinary staggered-adoption regressions. We check whether it also undoes the first-flag selection highlighted above. To answer it under the null, we need a baseline model for output, and we keep it as permissive as the fixed effects allow. We assume that, absent any flag, monthly output is

$$Y_{i,t} \sim \text{Poisson}(\nu_{i,t}), \qquad \nu_{i,t} = \exp(a_i + b_t),$$

independent across months. The two assumptions here are deliberately weak: a_i lets each author have an arbitrary productivity level, and b_t lets the overall climate move arbitrarily from month to month. Flagging is per-paper with probability p, and, as in the data, can occur only after ChatGPT's release. We write $J_t = 1$ for those post-release months and $J_t = 0$ before.

By the property of the Poisson distribution, if a month's output is Poisson with mean $\nu_{i,t}$ and each paper is flagged independently with probability p, then the flagged and unflagged counts are themselves independent Poisson variables,

$$F_{i,t} \sim \text{Poisson}(p\,\nu_{i,t}), \qquad Y_{i,t} - F_{i,t} \sim \text{Poisson}\big((1-p)\nu_{i,t}\big).$$

A no-flag month is one with $H_{i,t} = 0$, that is $F_{i,t} = 0$. Because the two counts are independent, conditioning on it removes the flagged piece and leaves expected output $\mathbb{E}[Y_{i,t} \mid H_{i,t} = 0, a_i] = (1 - p)\nu_{i,t}$. Applying this to each group of retained observations:

$$\underbrace{\mathbb{E}[Y_{i,t} \mid a_i, A_i = 0]}_{\text{never-flagged author}} = \begin{cases} \nu_{i,t}, & J_t = 0, \\ (1-p)\nu_{i,t}, & J_t = 1, \end{cases} \qquad \underbrace{\mathbb{E}[Y_{i,t} \mid a_i, G_i = c]}_{\text{flagged author, } t \neq c} = \begin{cases} \nu_{i,t}, & t < c,\ J_t = 0, \\ (1-p)\nu_{i,t}, & t < c,\ J_t = 1, \\ \nu_{i,t}, & t > c. \end{cases}$$

A never-flagged author is selected on no flag in every post-release month. A flagged author is selected on no flag only *before* adoption; after adoption the event definition no longer restricts that month, so its mean returns to $\nu_{i,t}$. Since adoption occurs after T_0, every retained post-adoption month has $J_t = 1$. Collecting the three cases, the expected output of every retained observation is exactly

$$\mathbb{E}[Y_{i,t} \mid a_i, A_i, C_i, t] = \exp\Big(\underbrace{a_i}_{\alpha_i} + \underbrace{b_t + J_t \log(1-p)}_{\lambda_{C_i,t}} + \underbrace{\big(-\log(1-p)\big)}_{\delta} A_i \operatorname{Post}_{i,t}\Big).$$

This is term for term the stacked-PPML model. The cohort-by-month fixed effect absorbs both the calendar climate b_t and the no-flag deflation $\log(1-p)$ that applies in post-release months; the treated-post coefficient $\delta = -\log(1-p)$ exactly undoes that deflation for flagged authors once they have adopted. The conclusion is the answer to the referee's question. Because the model's conditional mean is reproduced exactly, the population coefficient is exactly

$$\delta = -\log(1-p) > 0,$$

and this holds for arbitrary author productivity a_i, arbitrary calendar shocks b_t, any number of cohorts, never-flagged controls with random pseudo-dates, and both author and cohort-by-month fixed effects. Stacking, the never-flagged controls, and the fixed effects relocate the first-flag selection but do not remove it. If the detection month were kept, its expected output would be

$$\mathbb{E}[Y_{i,c} \mid a_i, G_i = c] = (1-p)\nu_{i,c} + \frac{p\,\nu_{i,c}}{1 - \exp(-p\,\nu_{i,c})},$$

the no-flag level plus an inflation term from conditioning on at least one flag ($H_{i,c} = 1$). That inflation depends on the author's own rate $\nu_{i,c}$, so the adoption month carries a separate, author-specific spike with no single coefficient.

5 Placebo exercises of Figure 1 in stacked-OLS event study

This section replicates the placebo exercises of Figure 1 using a stacked-OLS estimator instead of the Poisson pseudo-maximum-likelihood (PPML) used in the main text, confirming that the results are robust to the choice of estimator.

Results below do not show the first-treatment-month coefficients ($k = 0$), while section 10.2 below does.

5.1 With the dependent variable in levels (y = number of papers)

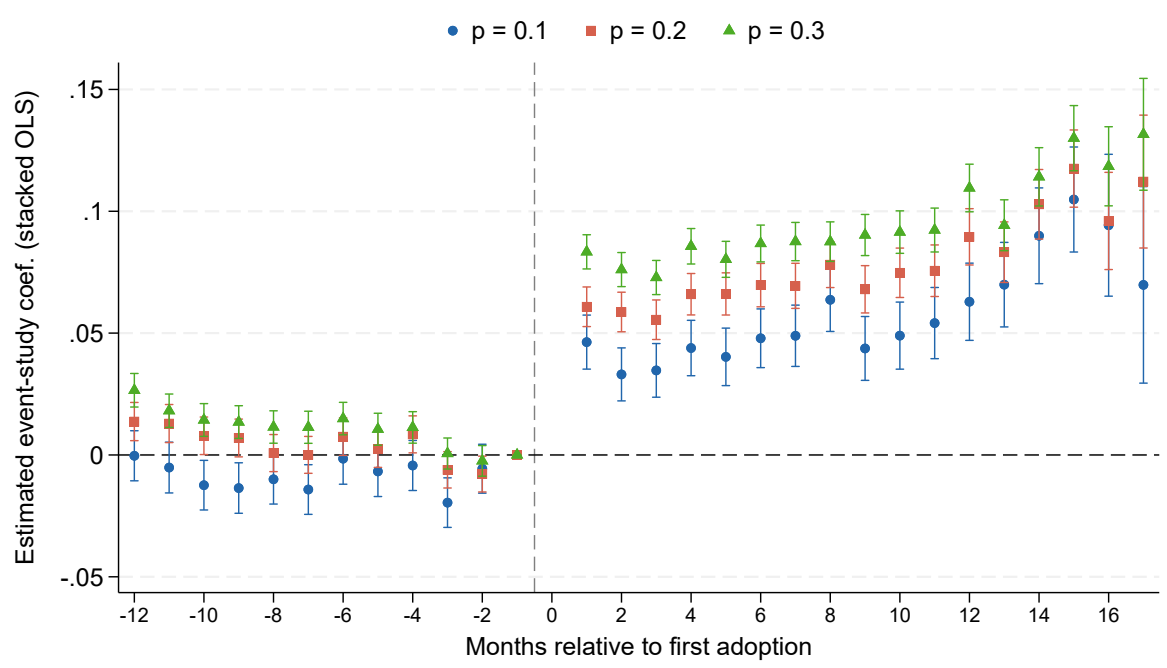

(A) **Placebo 1: random treatment**

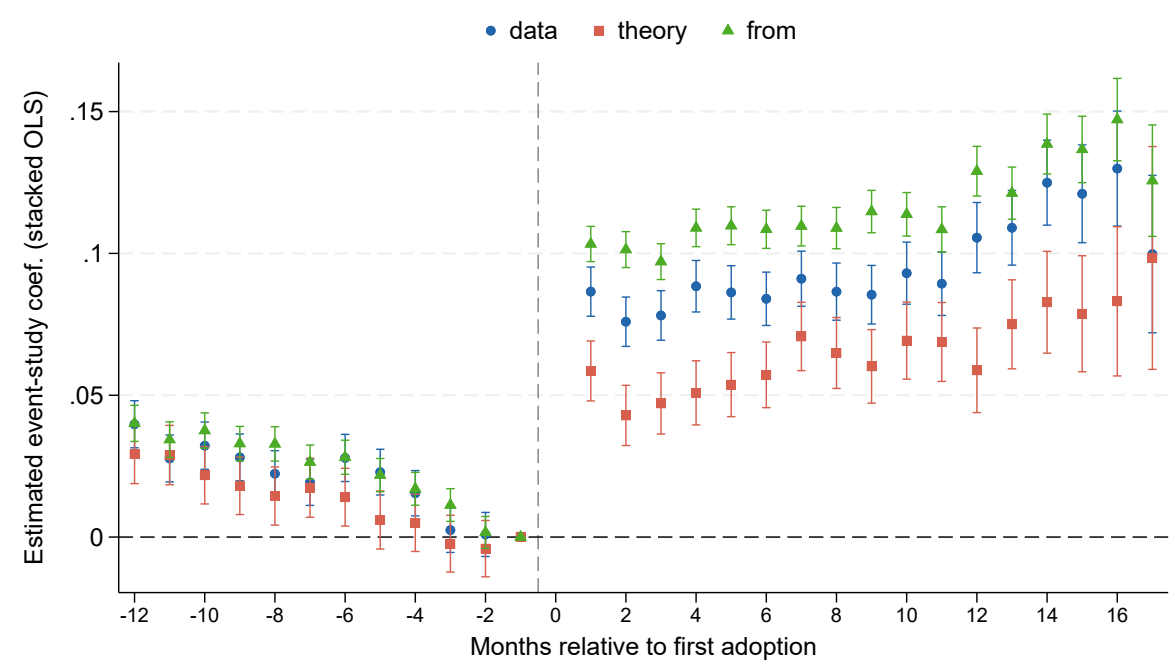

(B) **Placebo 2: keyword treatment**

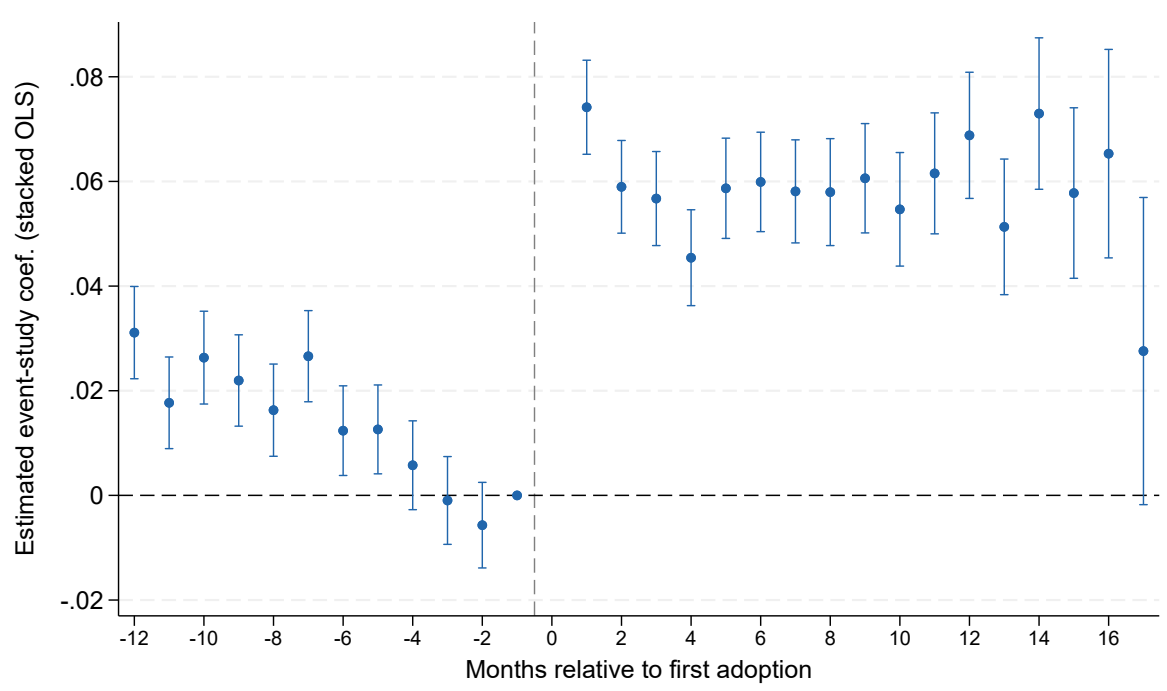

(C) **Placebo 3: pre-ChatGPT period**

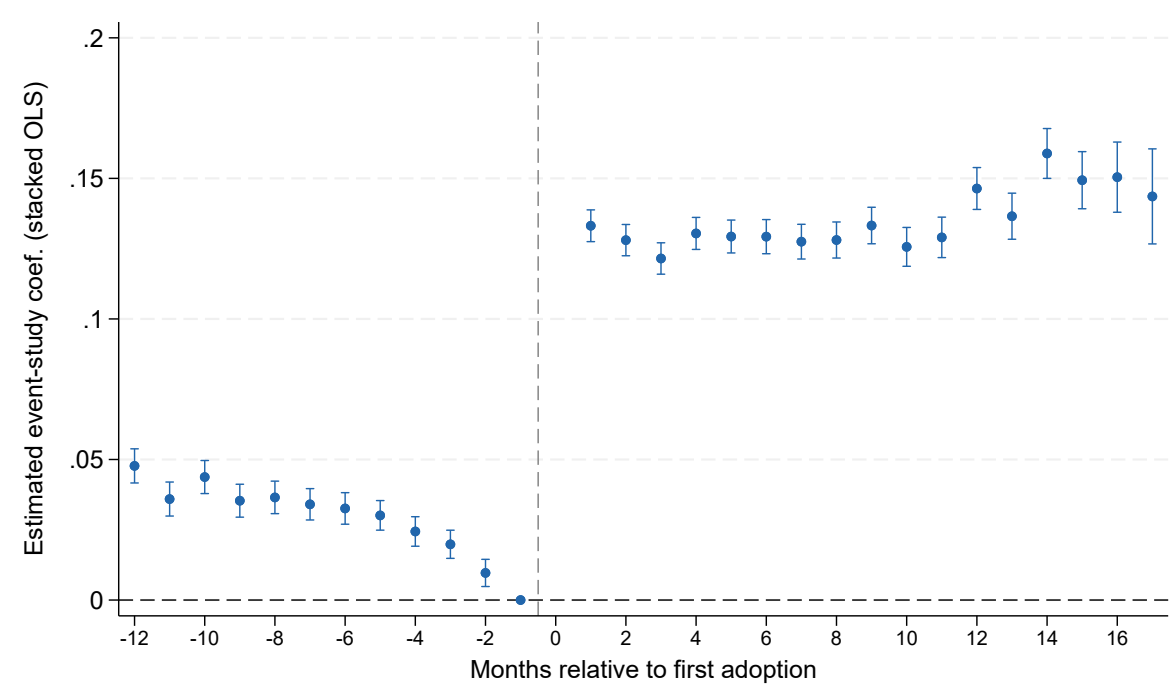

(D) **Placebo 4: inverted treatment**

Figure S4: **Placebo event studies.** All panels report stacked-OLS event-study coefficients, with 95% confidence intervals, of placebo exercises that replace the LLM detector with random paper-level flags (A), neutral keyword flags (B), an earlier observation window (C), and inverted treatment using $\alpha < 0.1$ instead of $\alpha > 0.1$ (D).

5.2 with $\ln(0.1 + y)$ as dependent variable

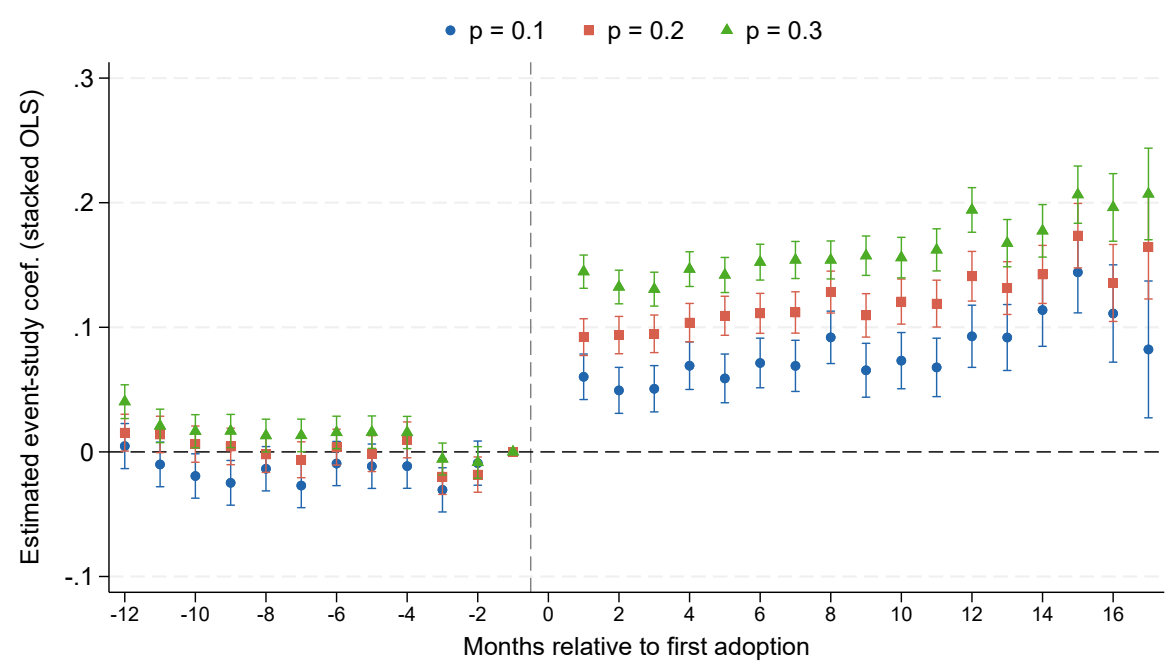

(A) **Placebo 1: random treatment**

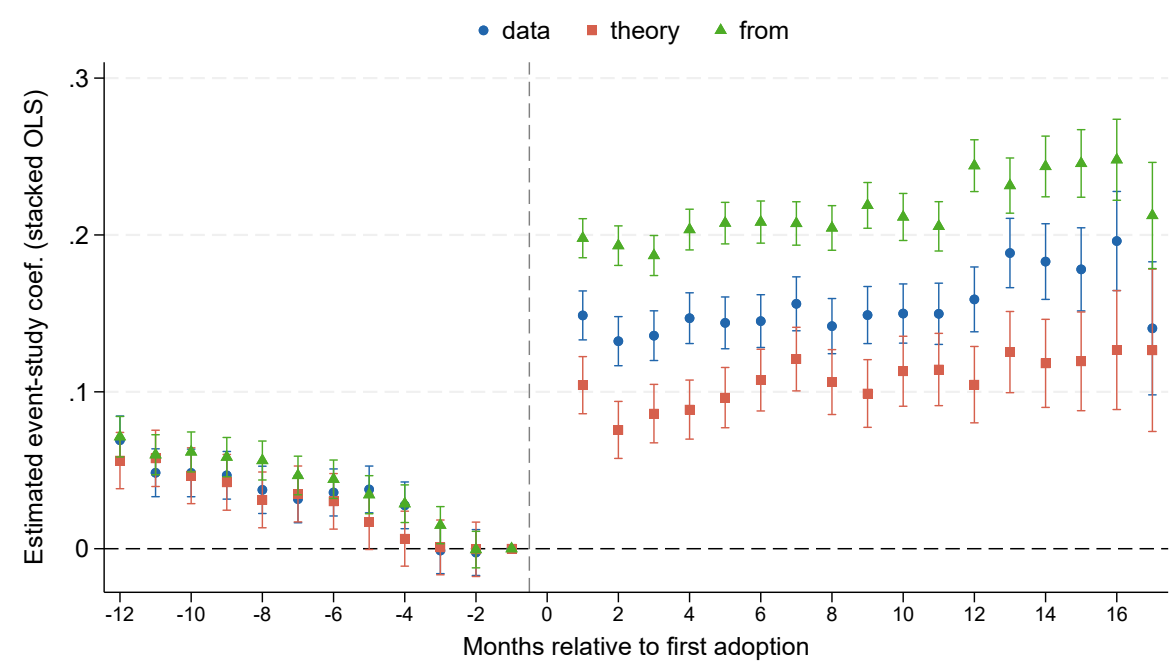

(B) **Placebo 2: keyword treatment**

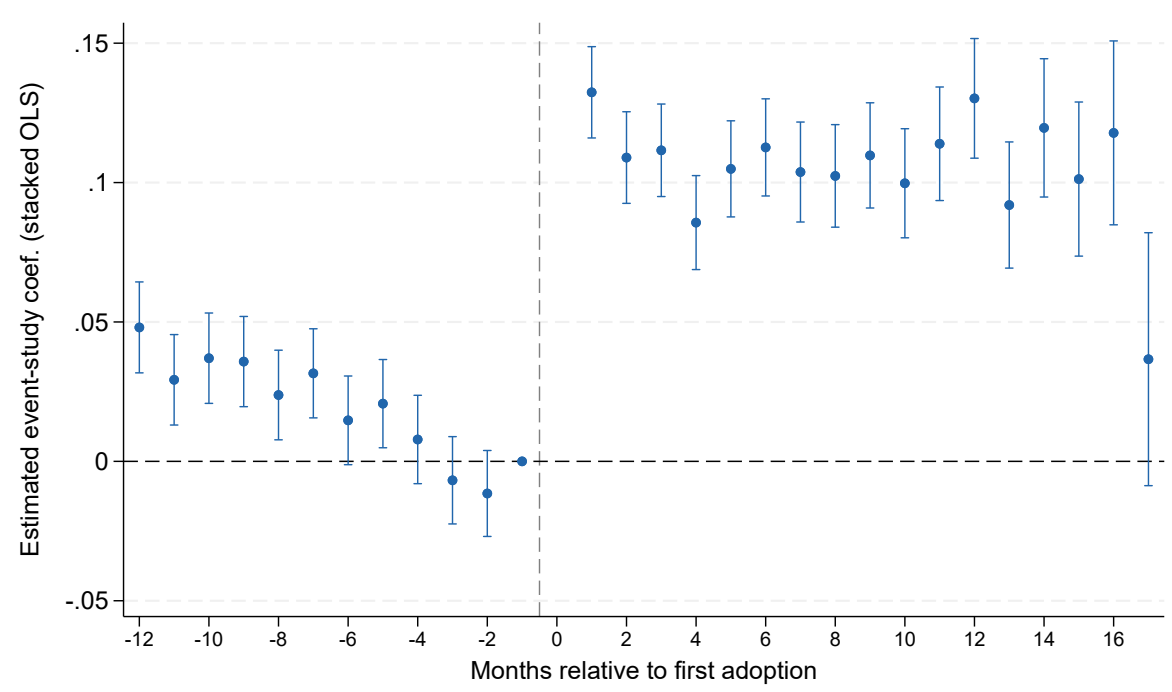

(C) **Placebo 3: pre-ChatGPT period**

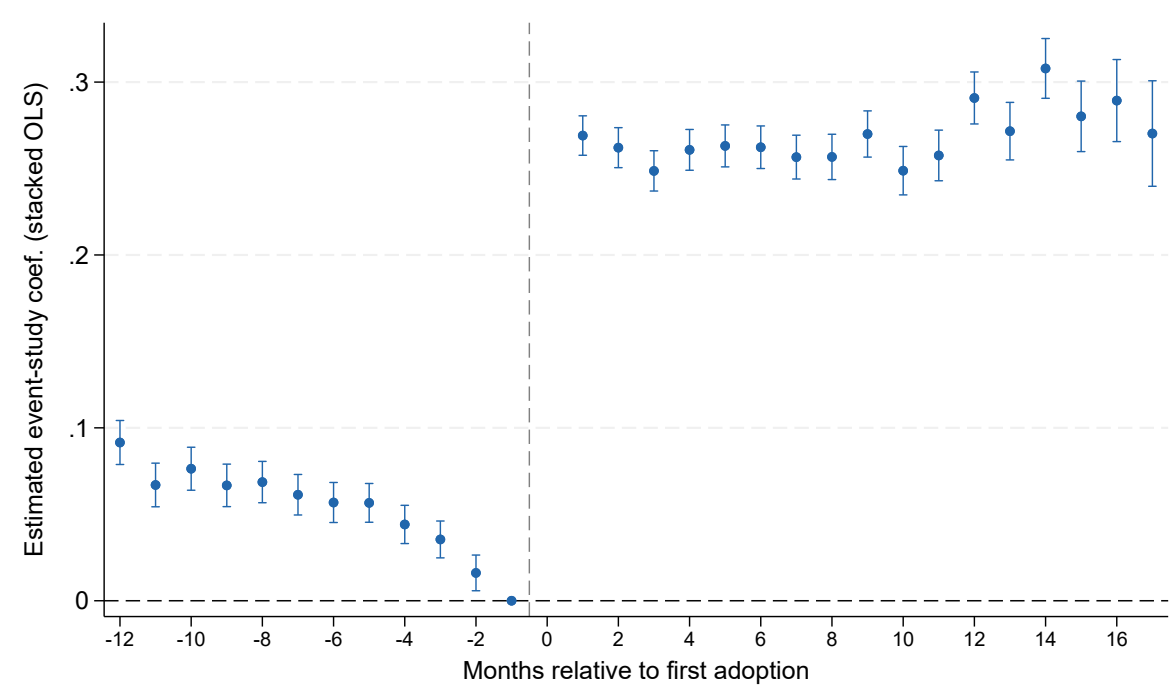

(D) **Placebo 4: inverted treatment**

Figure S5: **Placebo event studies.** All panels report stacked-OLS event-study coefficients, with 95% confidence intervals, of placebo exercises that replace the LLM detector with random paper-level flags (A), neutral keyword flags (B), an earlier observation window (C), and inverted treatment using $\alpha < 0.1$ instead of $\alpha > 0.1$ (D).

5.3 with $\ln(0.01 + y)$ as dependent variable

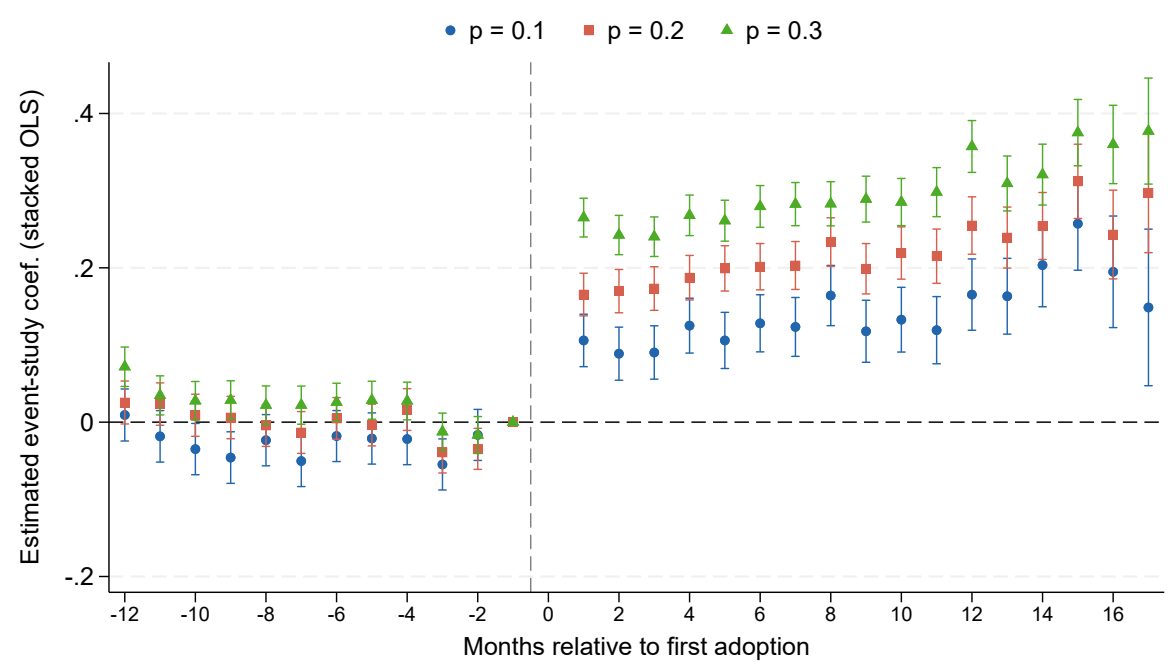

(A) **Placebo 1: random treatment**

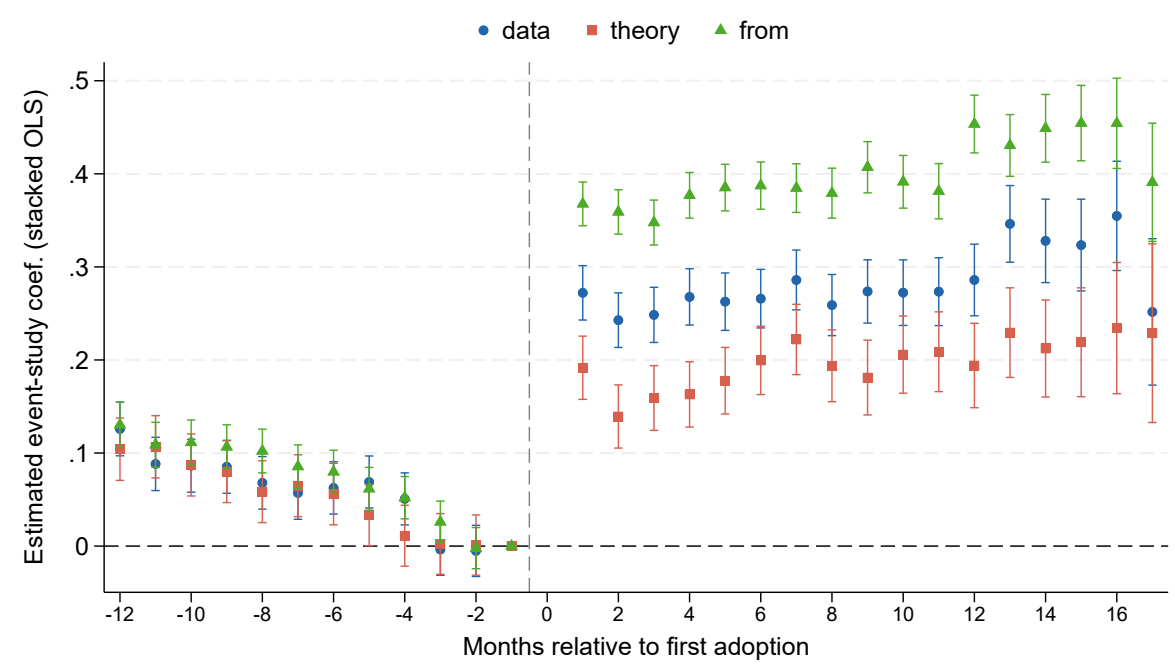

(B) **Placebo 2: keyword treatment**

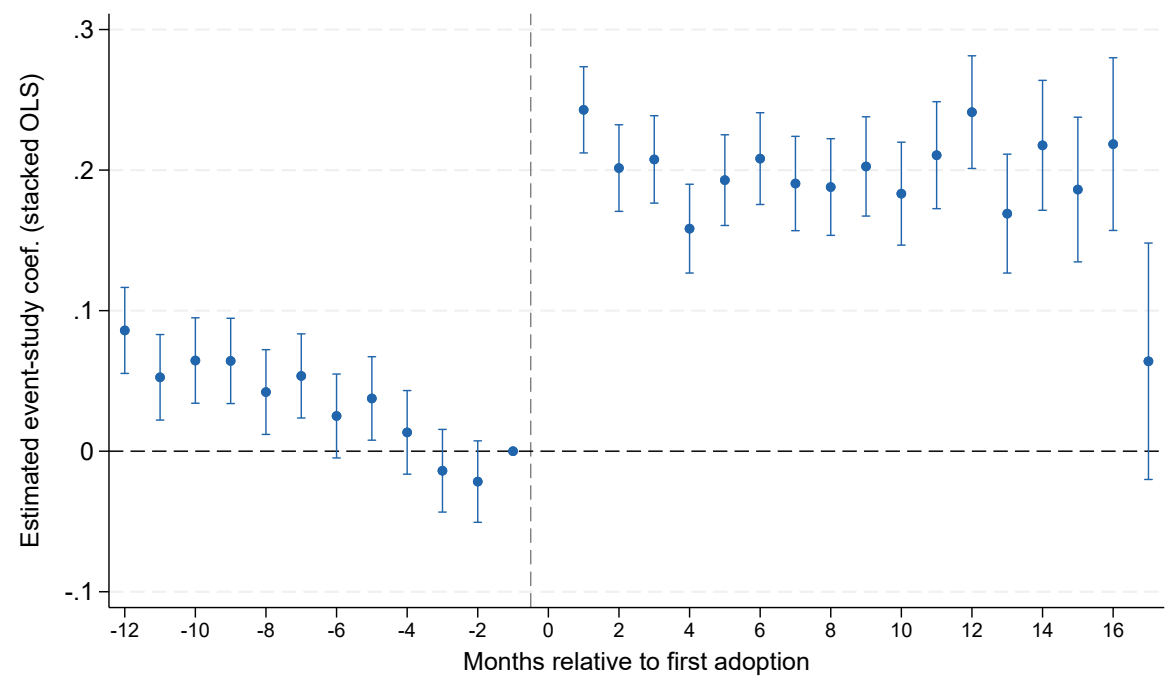

(C) **Placebo 3: pre-ChatGPT period**

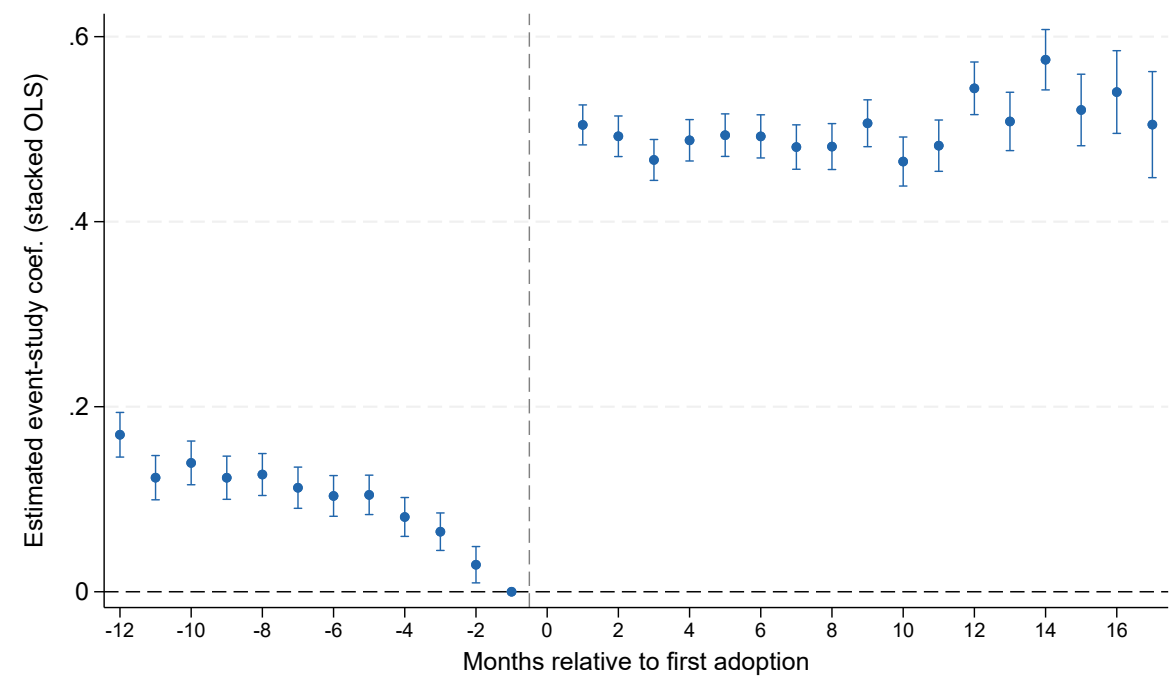

(D) **Placebo 4: inverted treatment**

Figure S6: **Placebo event studies.** All panels report stacked-OLS event-study coefficients, with 95% confidence intervals, of placebo exercises that replace the LLM detector with random paper-level flags (A), neutral keyword flags (B), an earlier observation window (C), and inverted treatment using $\alpha < 0.1$ instead of $\alpha > 0.1$ (D).

5.4 with $\ln(x+\sqrt{x^2+1})$ (inverse hyperbolic sine) as dependent variable

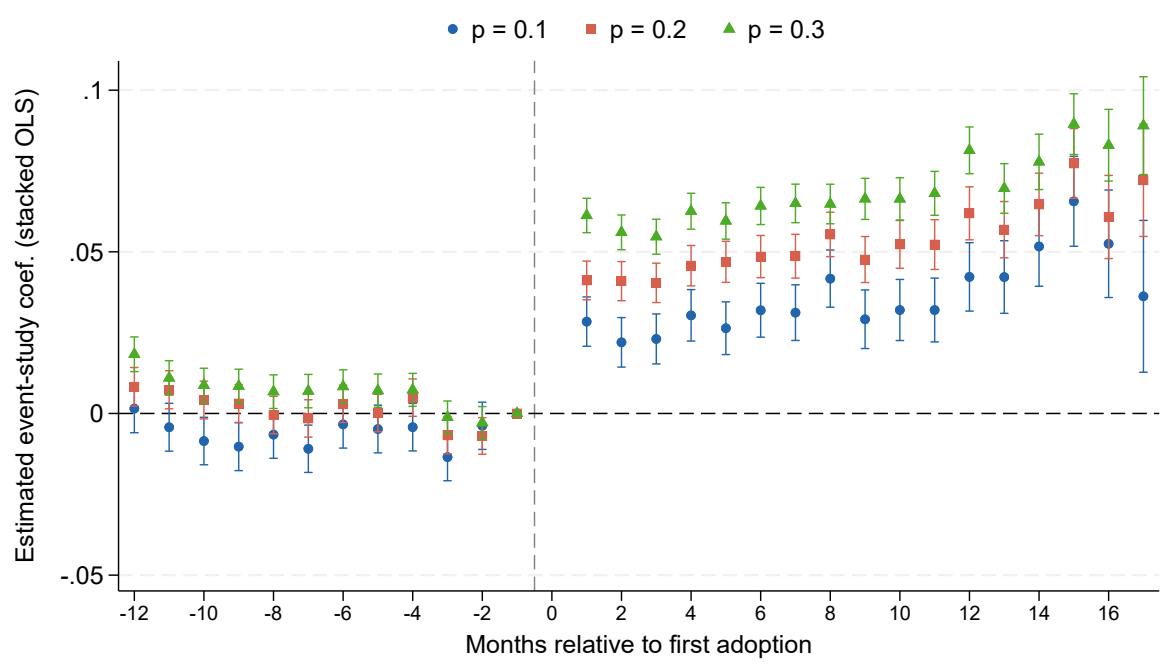

(A) **Placebo 1: random treatment**

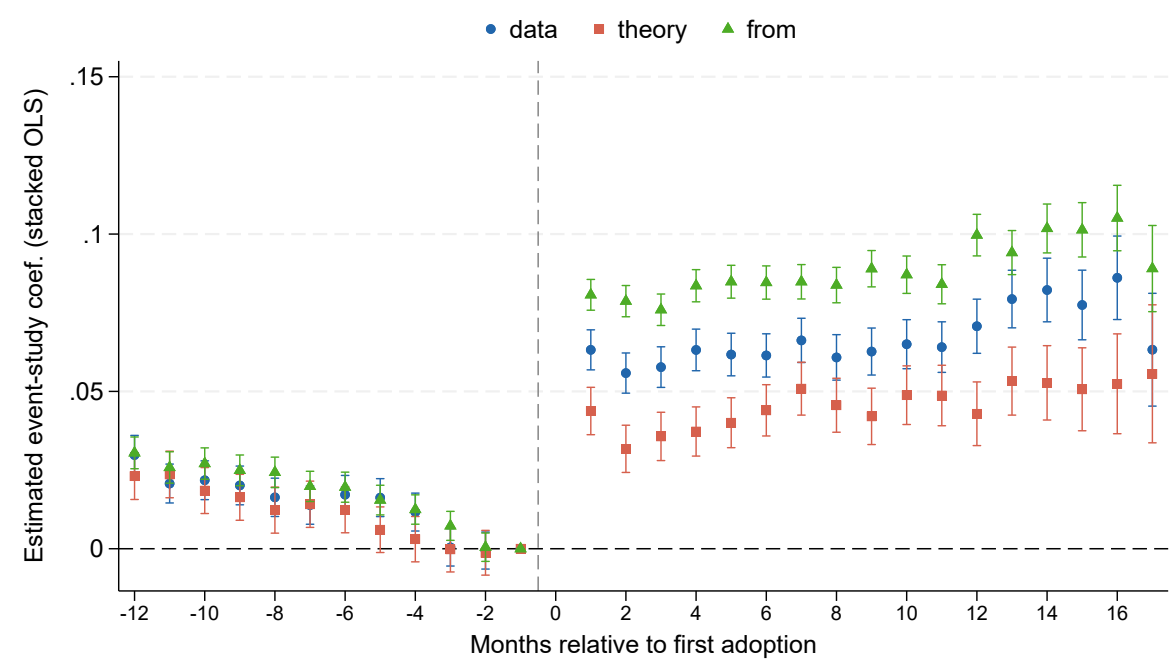

(B) **Placebo 2: keyword treatment**

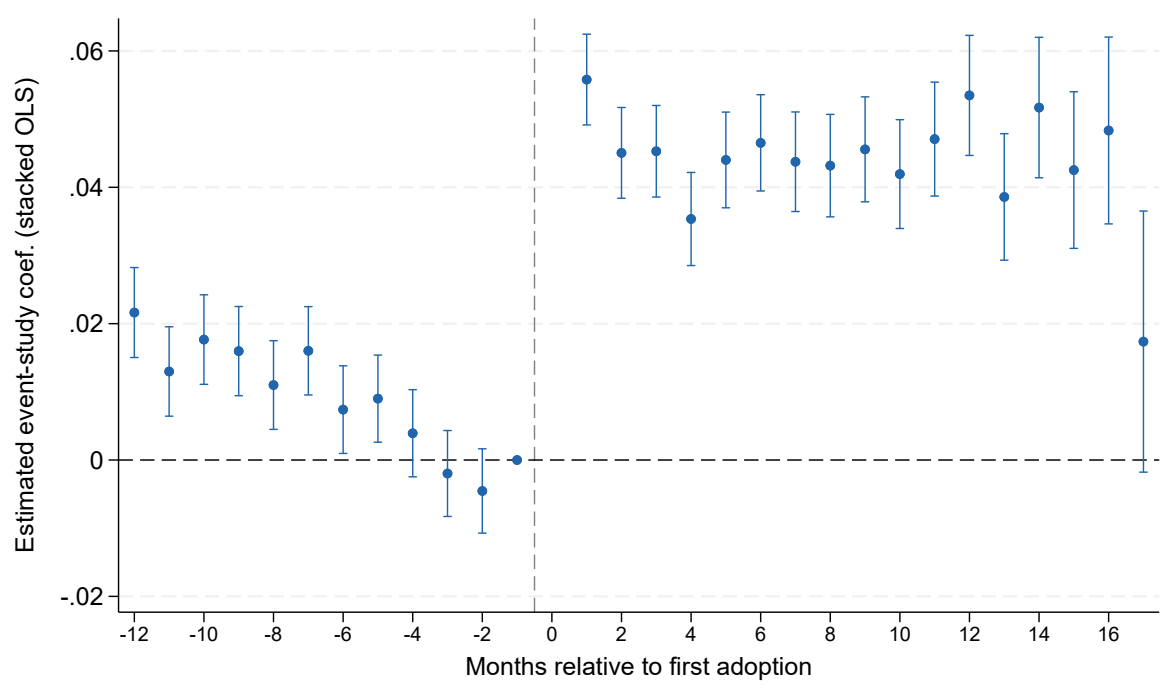

(C) **Placebo 3: pre-ChatGPT period**

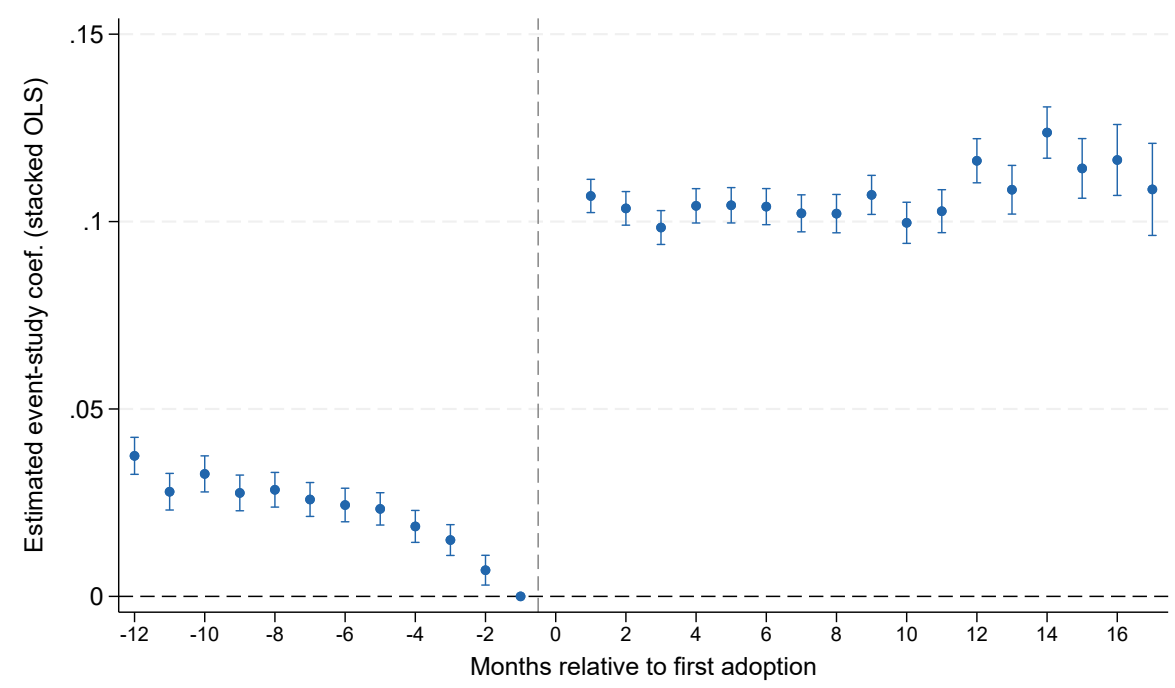

(D) **Placebo 4: inverted treatment**

Figure S7: **Placebo event studies.** All panels report stacked-OLS event-study coefficients, with 95% confidence intervals, of placebo exercises that replace the LLM detector with random paper-level flags (A), neutral keyword flags (B), an earlier observation window (C), and inverted treatment using $\alpha < 0.1$ instead of $\alpha > 0.1$ (D).

6 Placebo exercises of Figure 1 with modern event-study estimators for staggered designs

This section replicates the placebo exercises of Figure 1 using modern event-study estimators designed for staggered treatment adoption.

Results below do not show the first-treatment-month coefficients ($k = 0$), while section 10.3 below does.

6.1 de Chaisemartin and d'Haultfœuille's (2026) estimator

6.1.1 with the dependent variable in levels (y = number of papers)

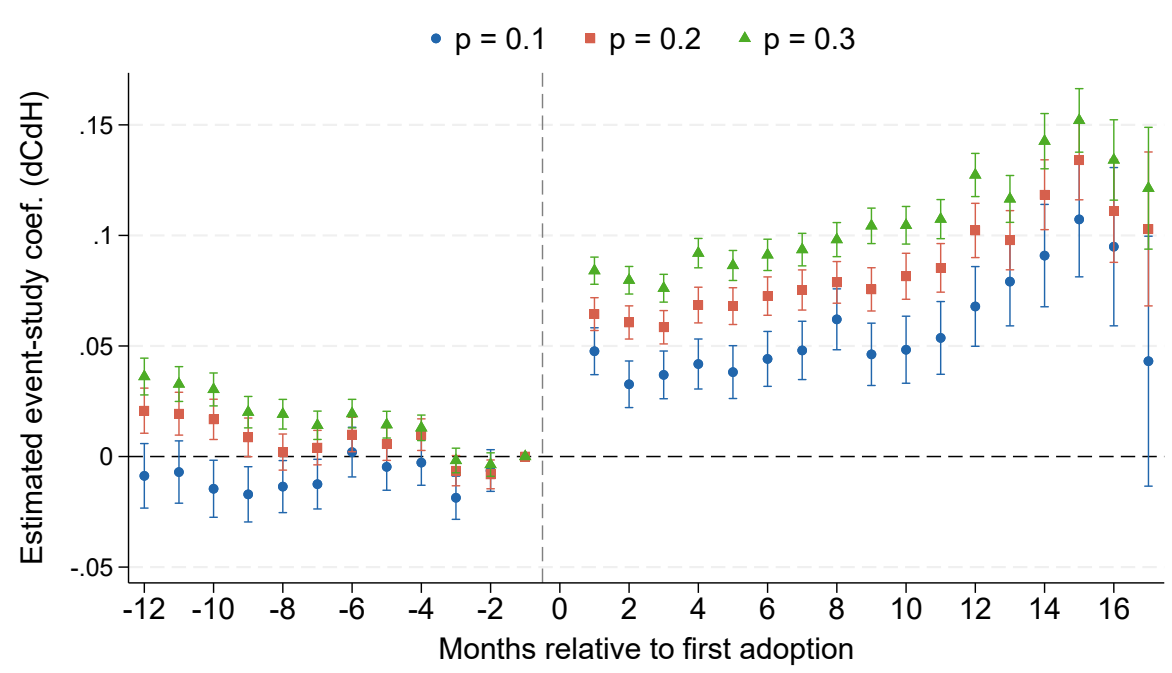

(A) **Placebo 1: random treatment**

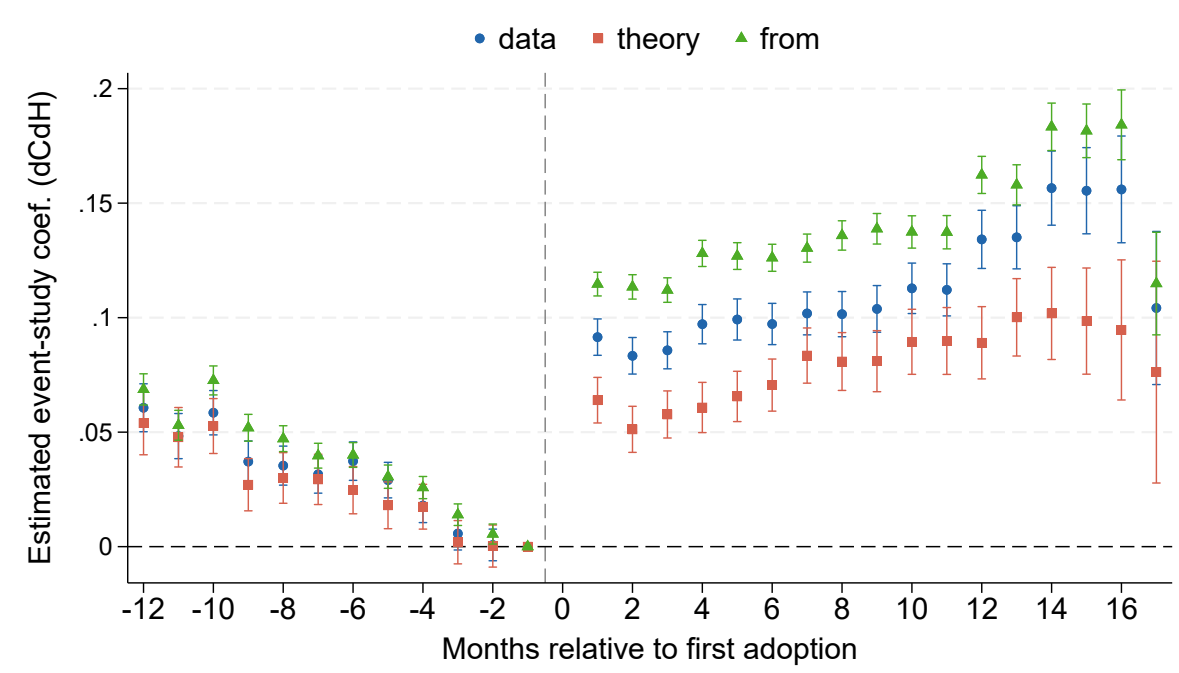

(B) **Placebo 2: keyword treatment**

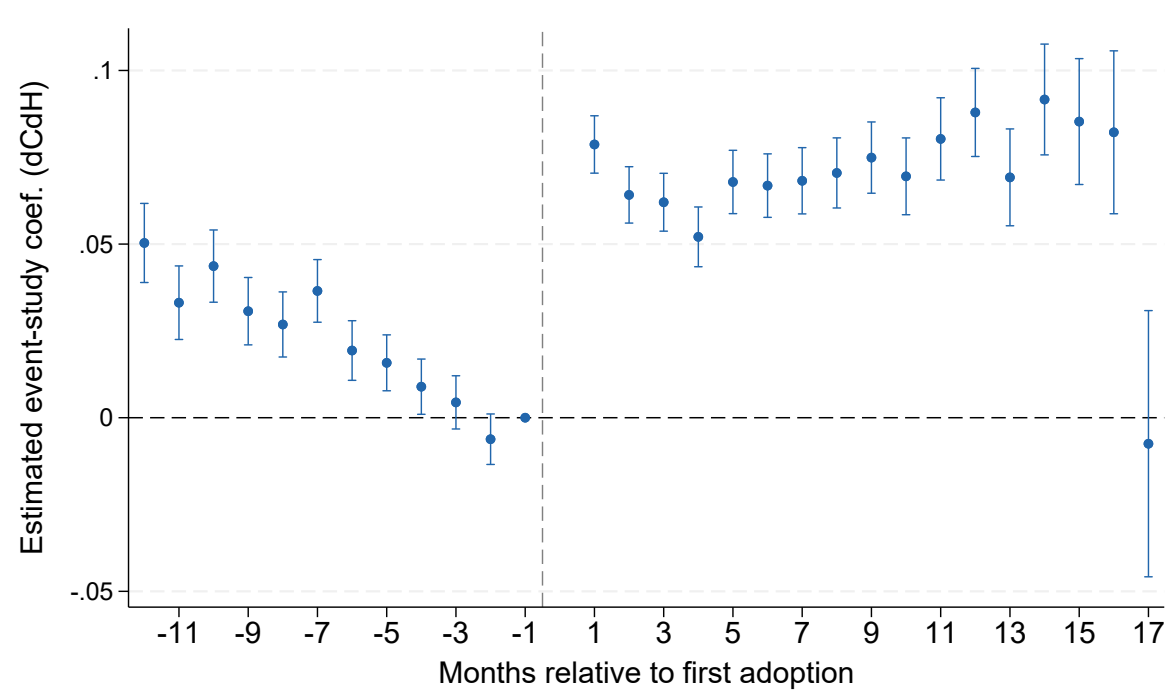

(C) **Placebo 3: pre-ChatGPT period**

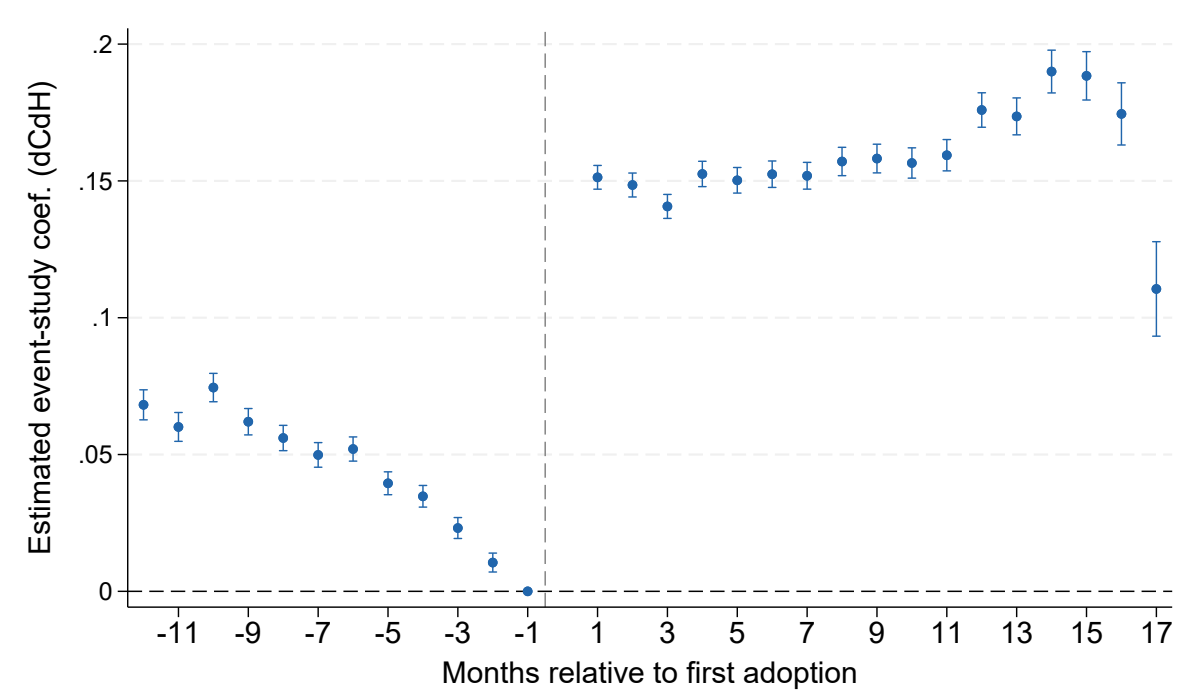

(D) **Placebo 4: inverted treatment**

Figure S8: **Placebo event studies.** All panels report de Chaisemartin and d'Haultfœuille's (2026) event-study coefficients, with 95% confidence intervals, of placebo exercises that replace the LLM detector with random paper-level flags (A), neutral keyword flags (B), an earlier observation window (C), and inverted treatment using $\alpha < 0.1$ instead of $\alpha > 0.1$ (D).

6.1.2 with $\ln(0.1 + y)$ as dependent variable

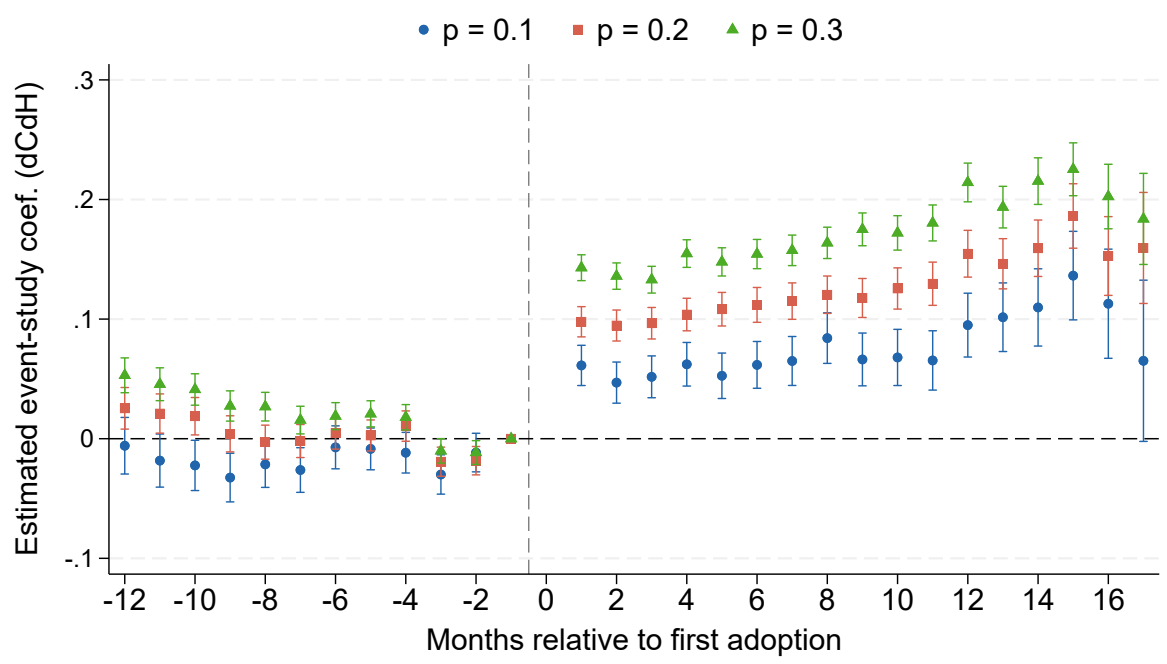

(A) **Placebo 1: random treatment**

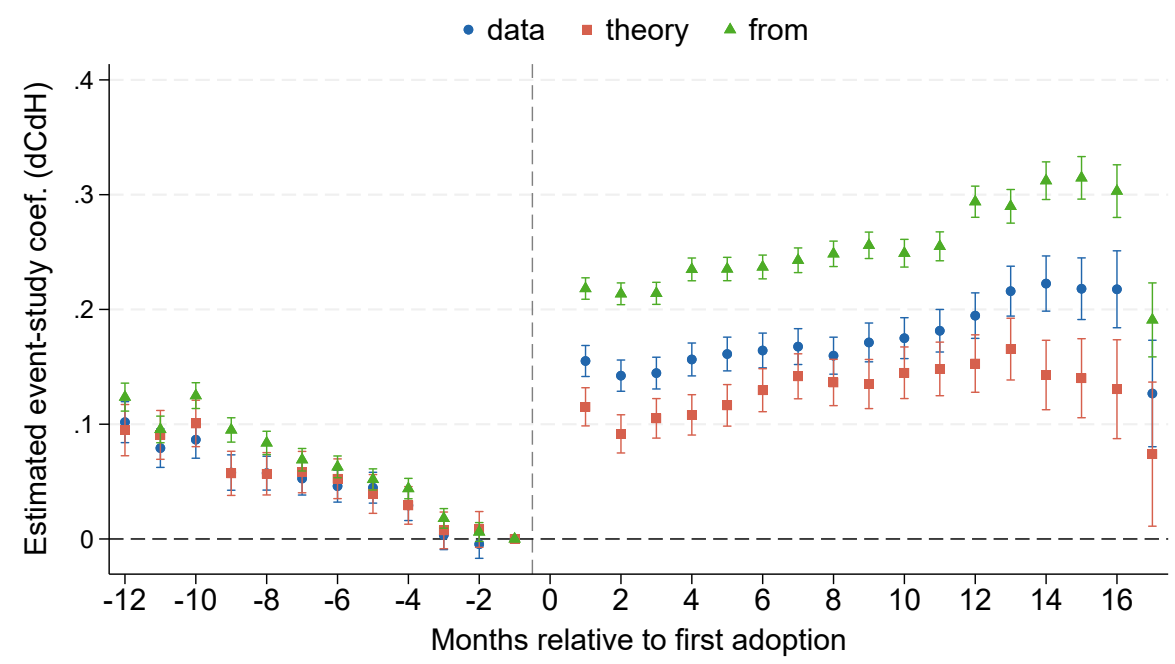

(B) **Placebo 2: keyword treatment**

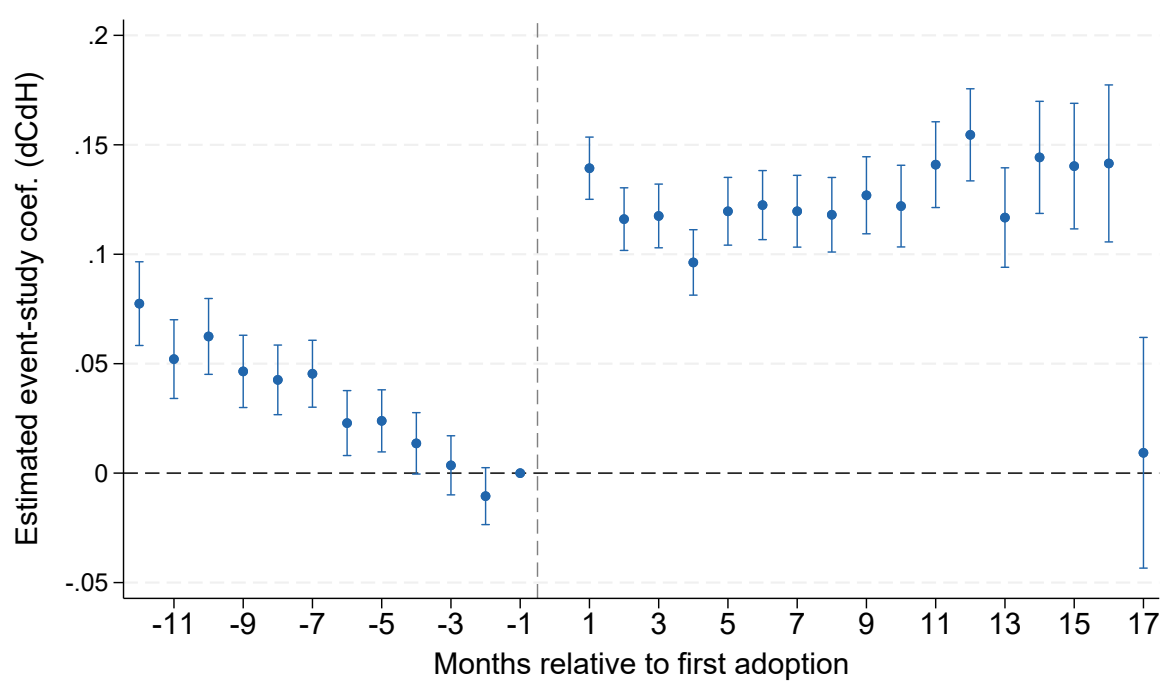

(C) **Placebo 3: pre-ChatGPT period**

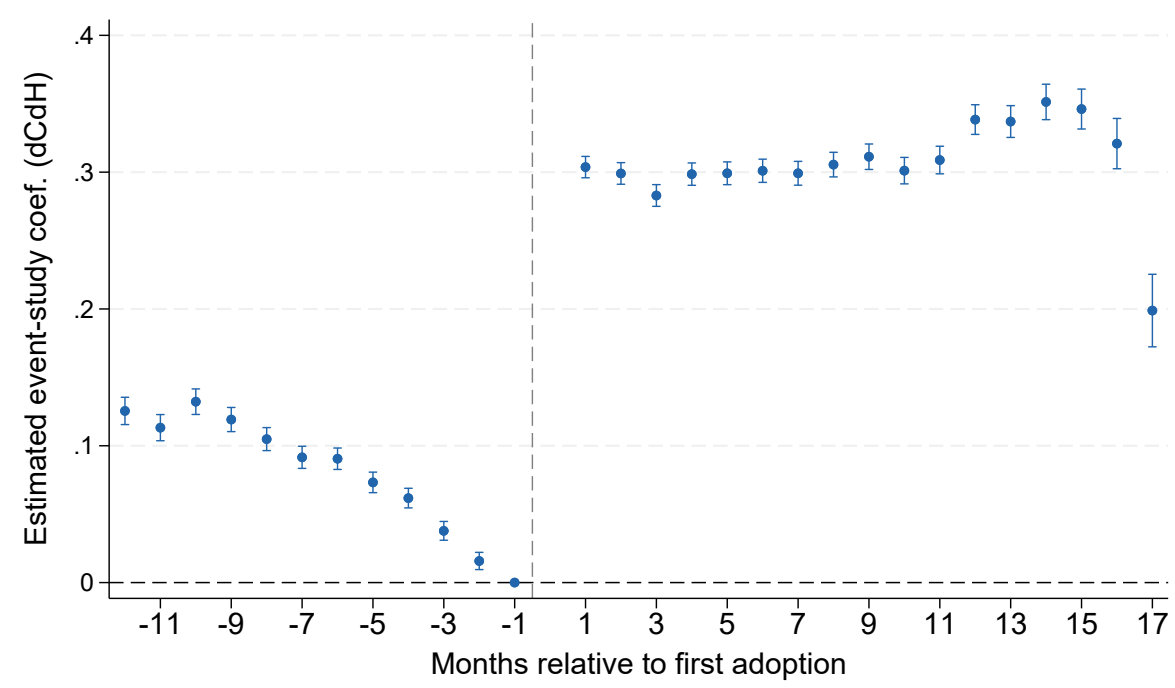

(D) **Placebo 4: inverted treatment**

Figure S9: **Placebo event studies.** All panels report de Chaisemartin and d'Haultfœuille's (2026) event-study coefficients, with 95% confidence intervals, of placebo exercises that replace the LLM detector with random paper-level flags (A), neutral keyword flags (B), an earlier observation window (C), and inverted treatment using $\alpha < 0.1$ instead of $\alpha > 0.1$ (D).

6.1.3 with $\ln(0.01 + y)$ as dependent variable

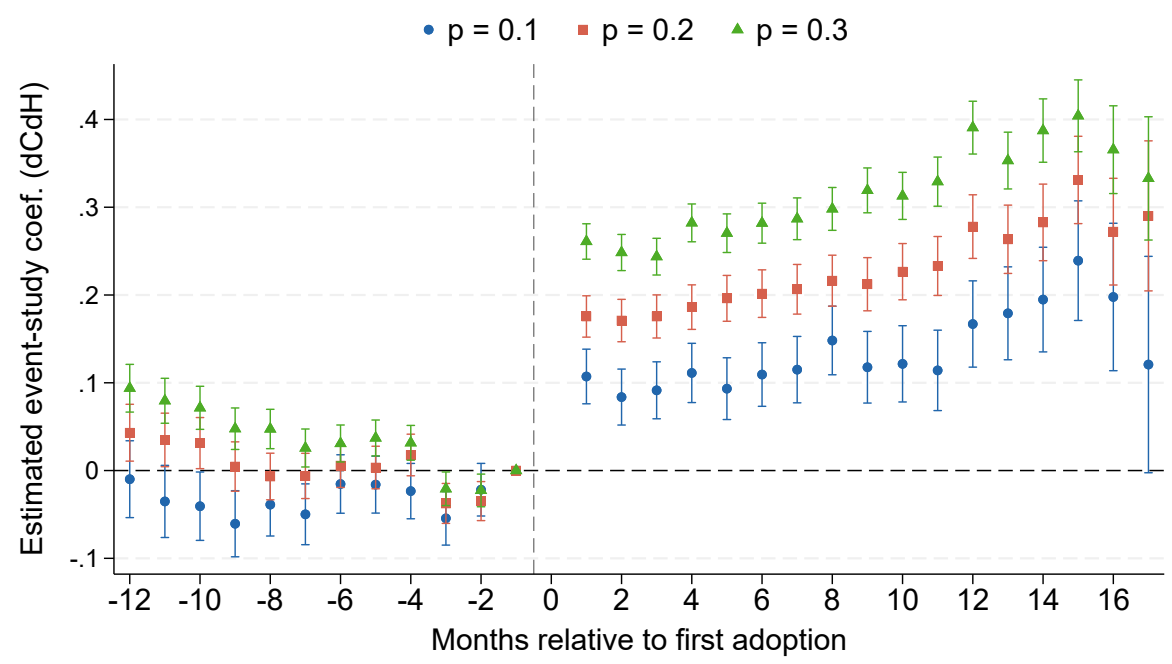

(A) **Placebo 1: random treatment**

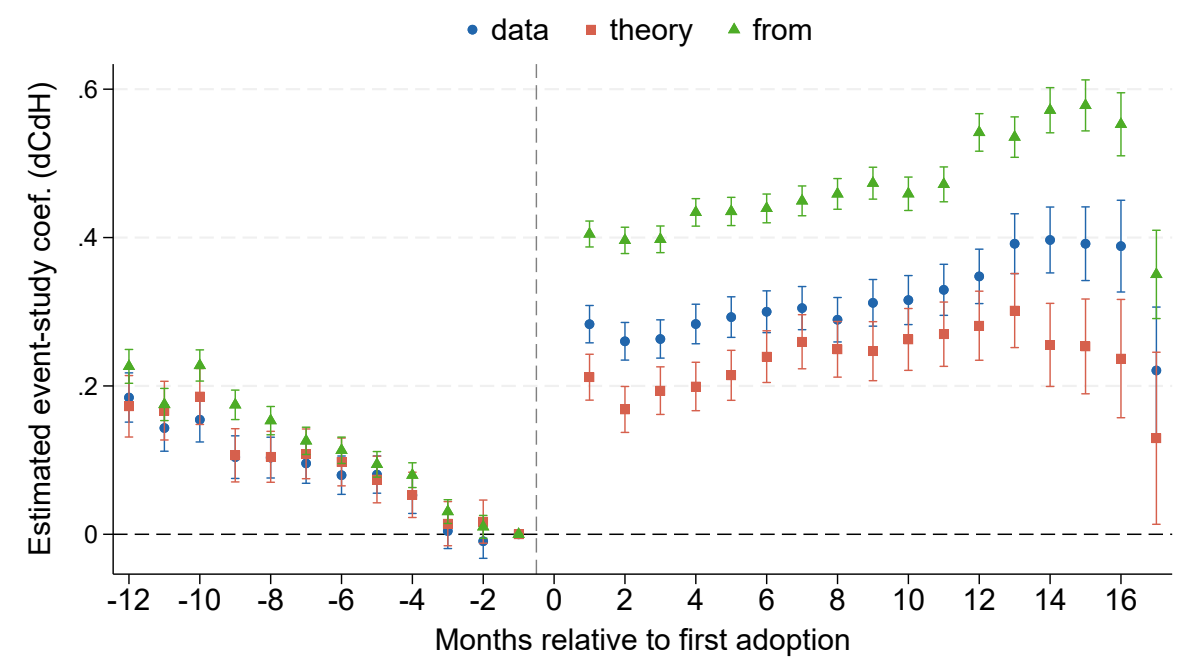

(B) **Placebo 2: keyword treatment**

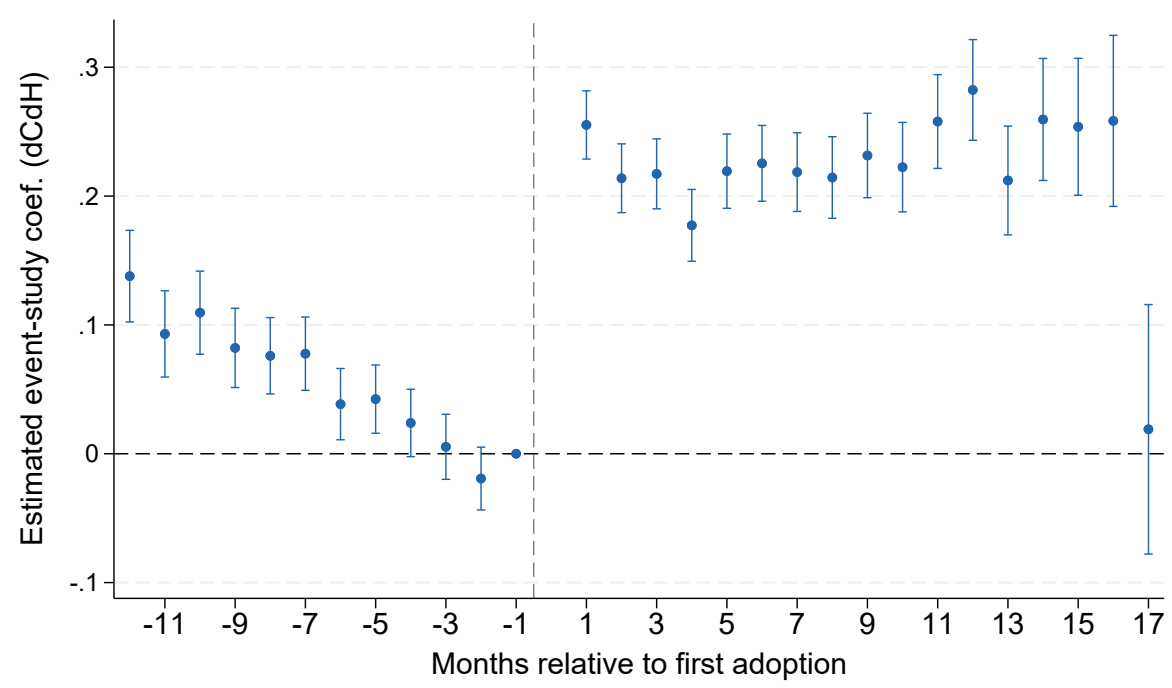

(C) **Placebo 3: pre-ChatGPT period**

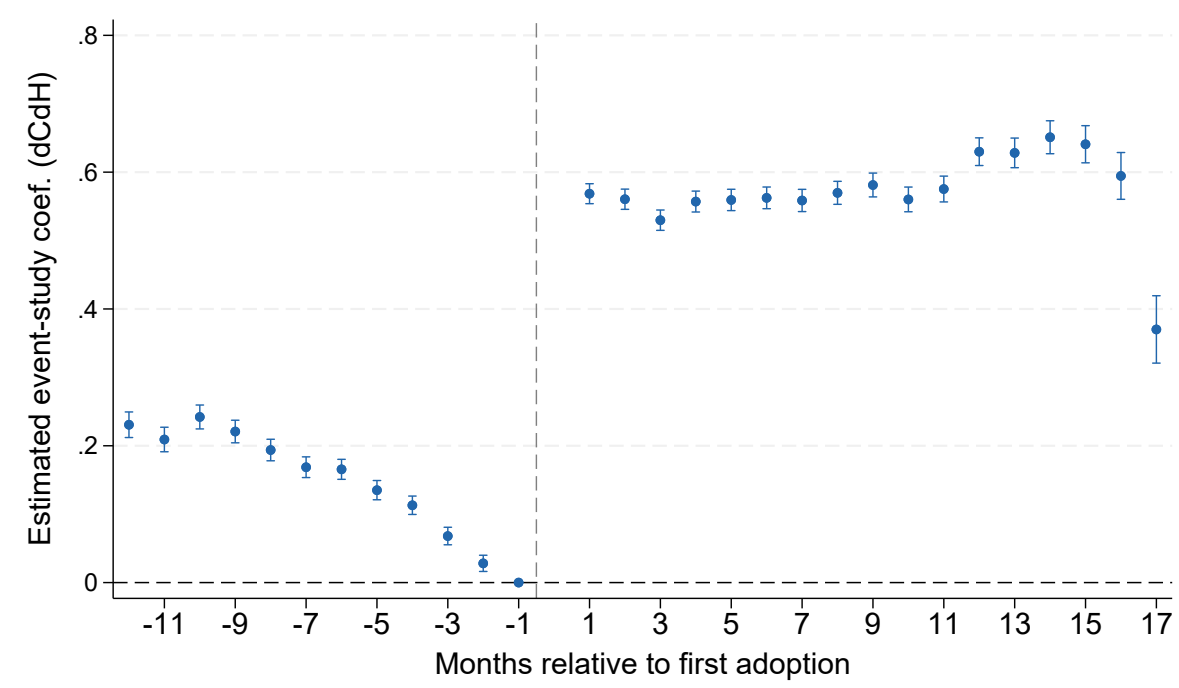

(D) **Placebo 4: inverted treatment**

Figure S10: **Placebo event studies.** All panels report de Chaisemartin and d'Haultfœuille's (2026) event-study coefficients, with 95% confidence intervals, of placebo exercises that replace the LLM detector with random paper-level flags (A), neutral keyword flags (B), an earlier observation window (C), and inverted treatment using $\alpha < 0.1$ instead of $\alpha > 0.1$ (D).

6.1.4 with $\ln(x + \sqrt{x^2 + 1})$ (inverse hyperbolic sine) as dependent variable

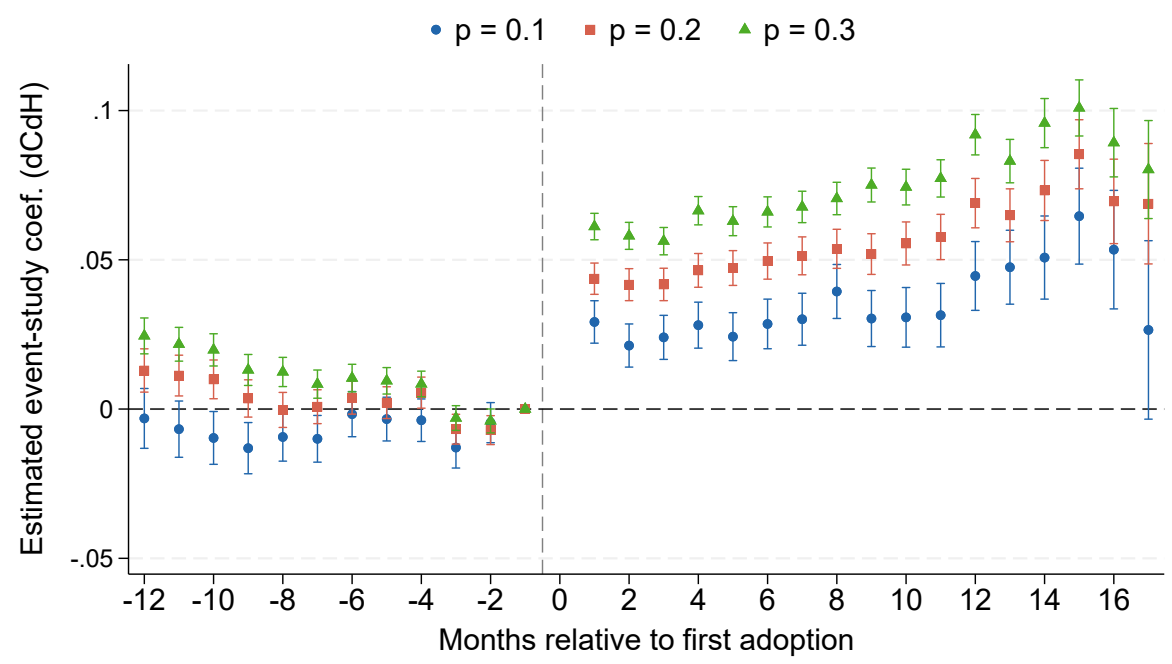

(A) **Placebo 1: random treatment**

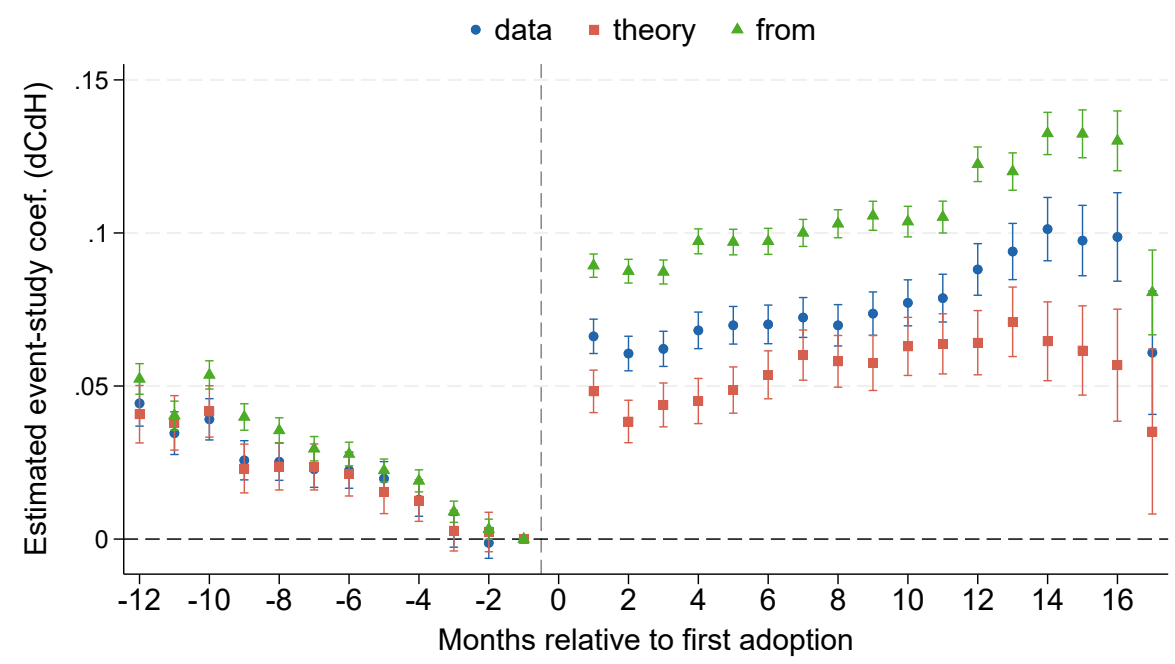

(B) **Placebo 2: keyword treatment**

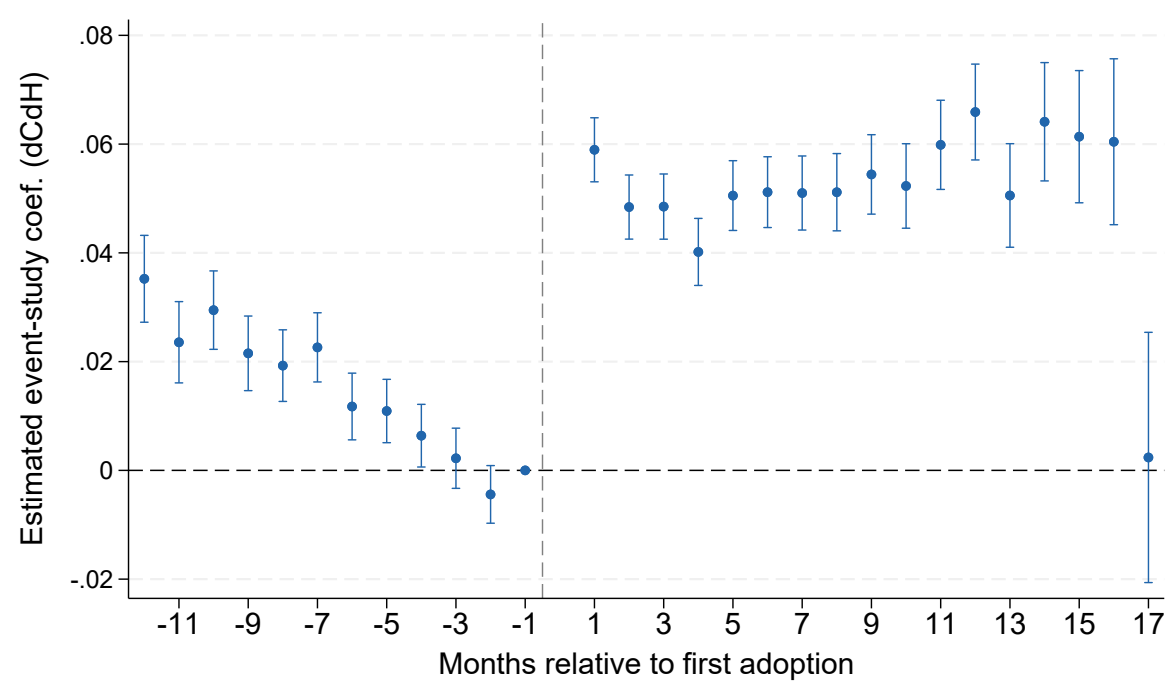

(C) **Placebo 3: pre-ChatGPT period**

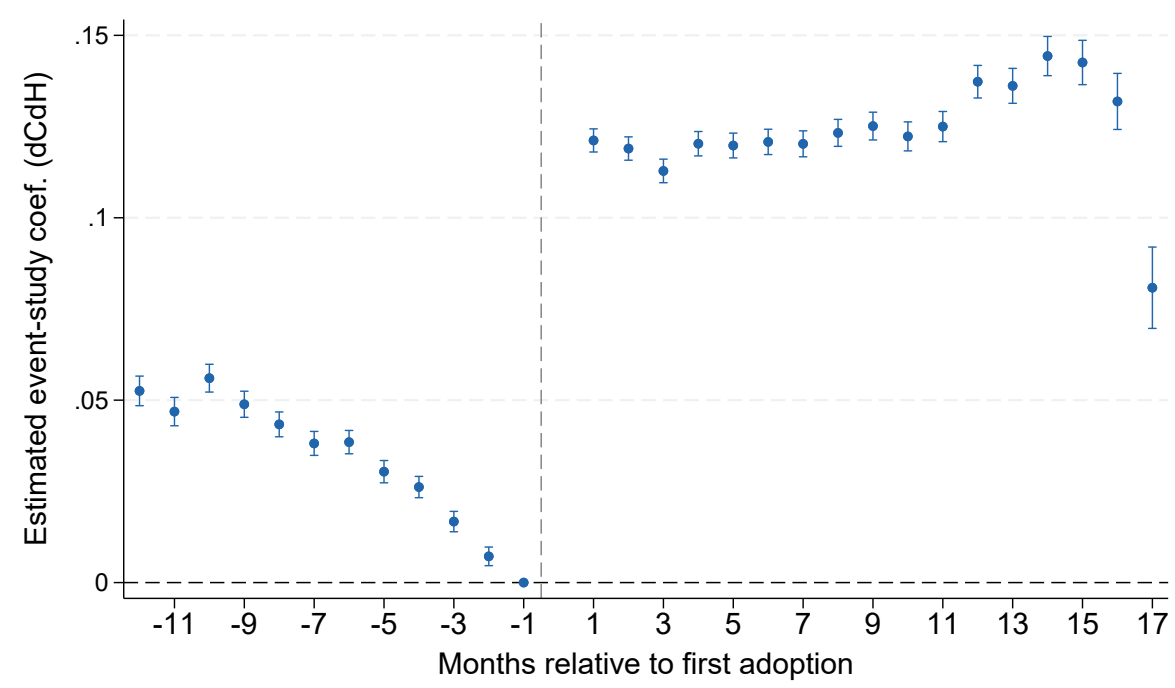

(D) **Placebo 4: inverted treatment**

Figure S11: **Placebo event studies.** All panels report de Chaisemartin and d'Haultfœuille's (2026) event-study coefficients, with 95% confidence intervals, of placebo exercises that replace the LLM detector with random paper-level flags (A), neutral keyword flags (B), an earlier observation window (C), and inverted treatment using $\alpha < 0.1$ instead of $\alpha > 0.1$ (D).

6.2 Sun and Abraham's (2021) estimator

6.2.1 with the dependent variable in levels (y = number of papers)

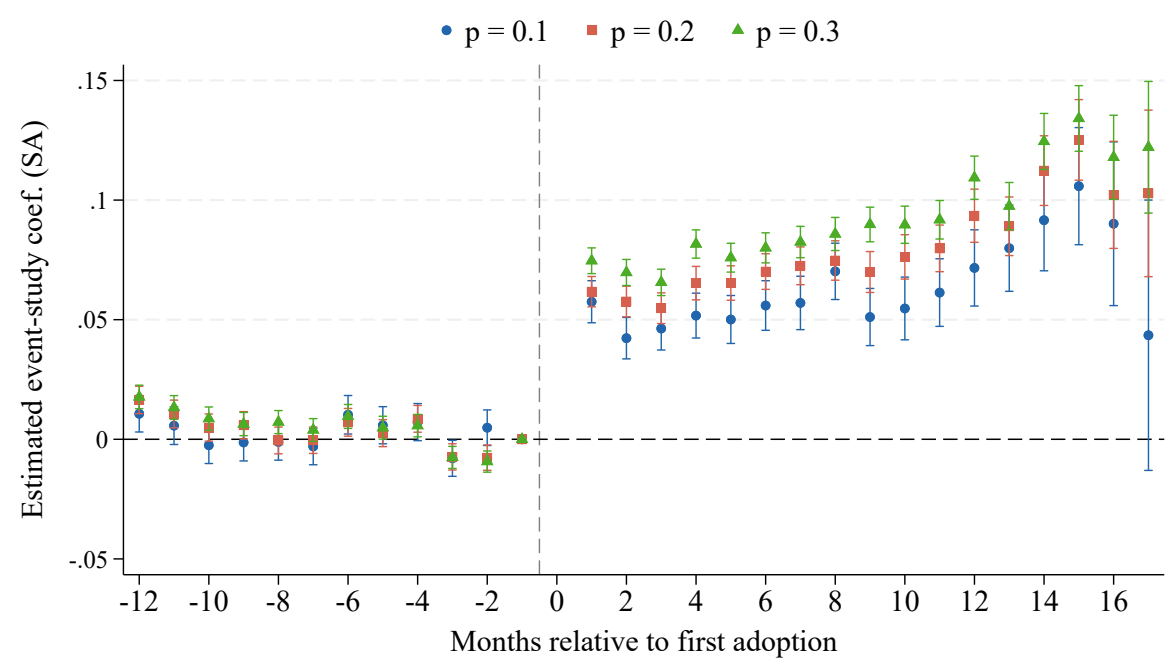

(A) **Placebo 1: random treatment**

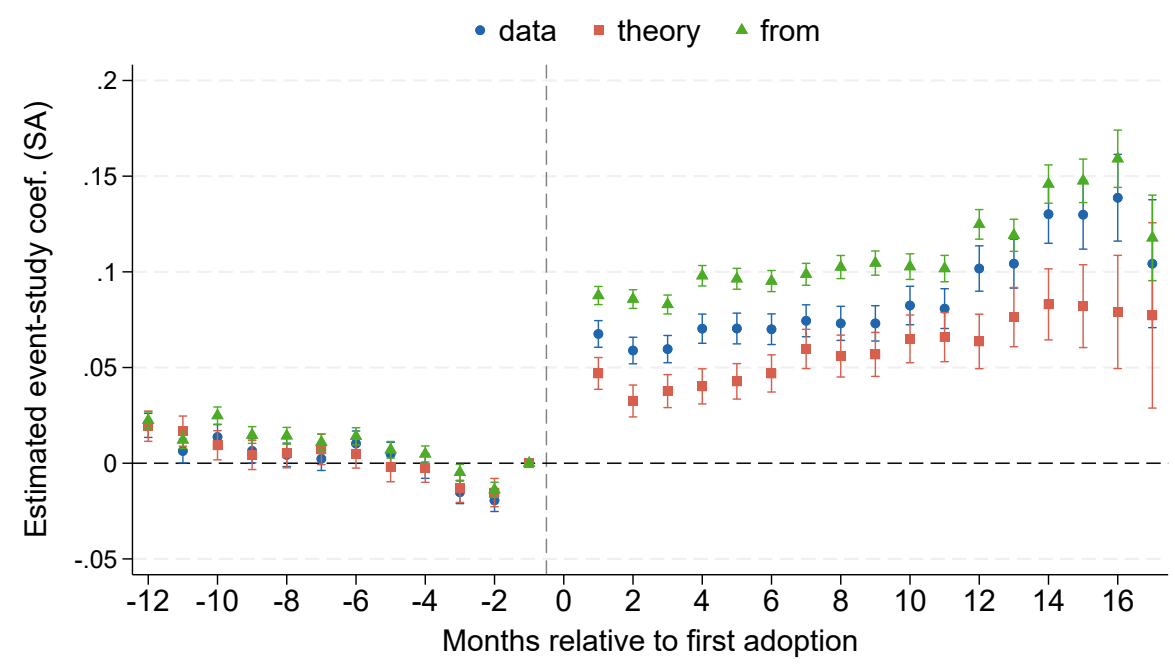

(B) **Placebo 2: keyword treatment**

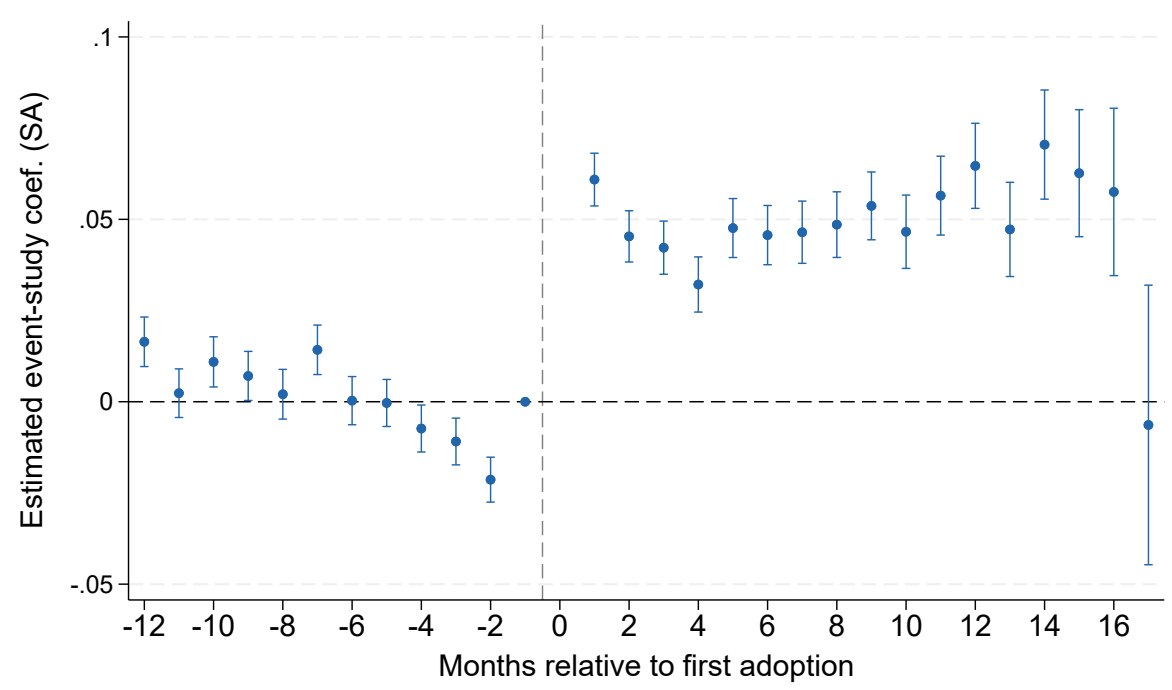

(C) **Placebo 3: pre-ChatGPT period**

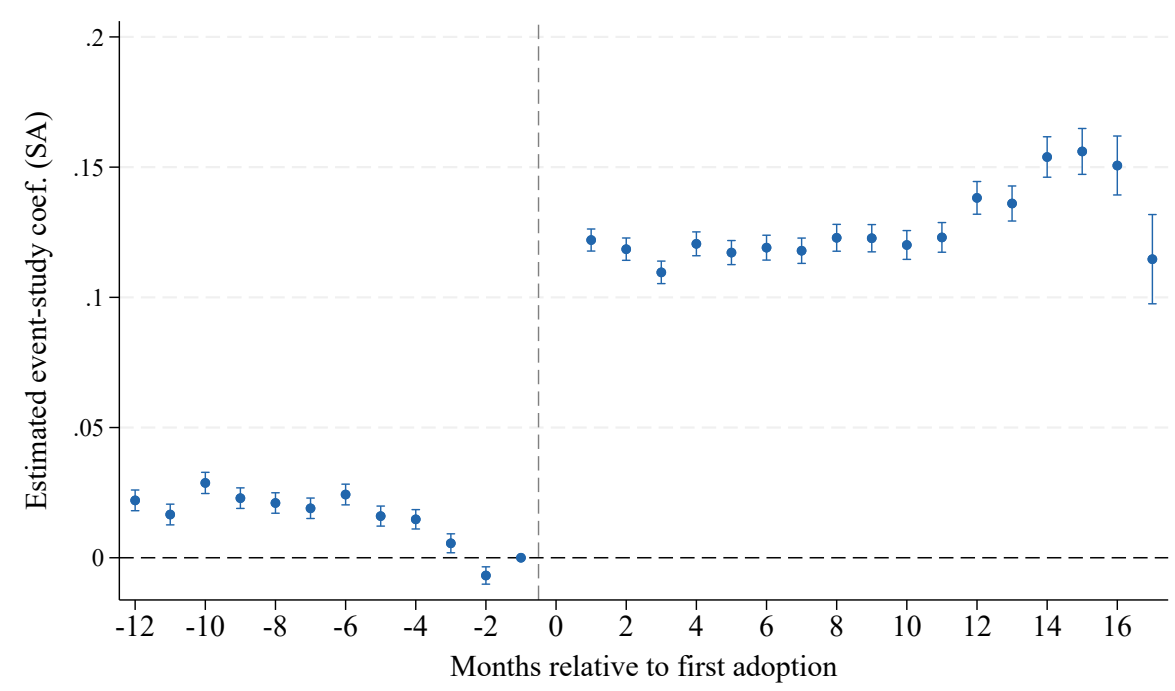

(D) **Placebo 4: inverted treatment**

Figure S12: **Placebo event studies.** All panels report Sun and Abraham's (2021) event-study coefficients, with 95% confidence intervals, of placebo exercises that replace the LLM detector with random paper-level flags (A), neutral keyword flags (B), an earlier observation window (C), and inverted treatment using $\alpha < 0.1$ instead of $\alpha > 0.1$ (D).

6.2.2 with $\ln(0.1 + y)$ as dependent variable

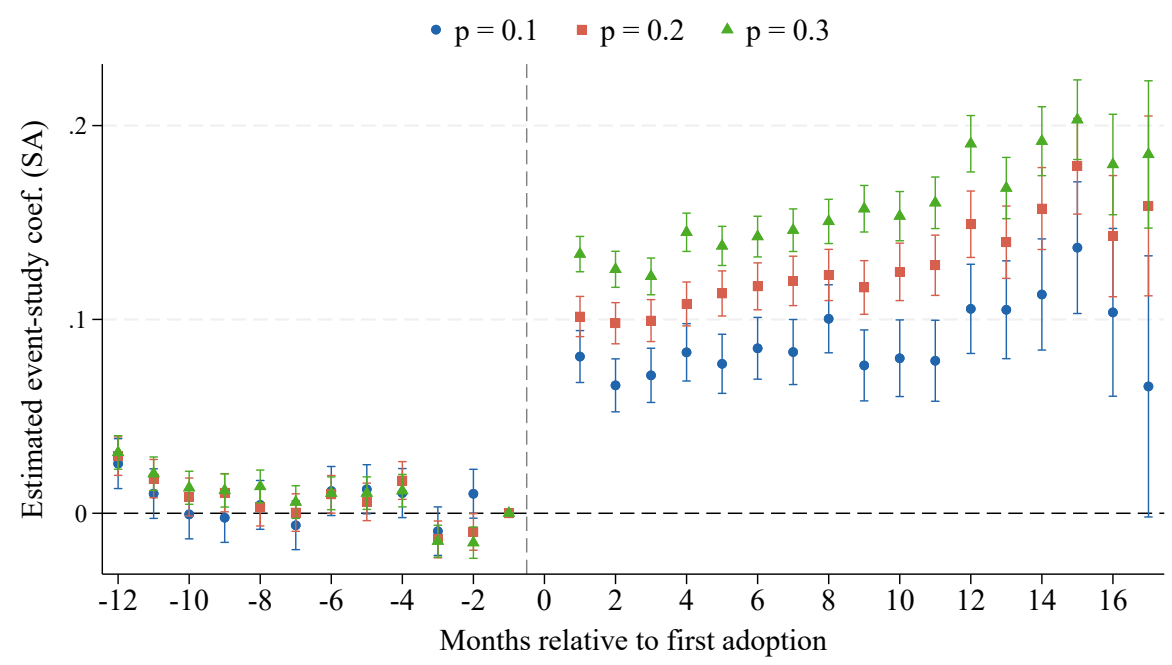

(A) **Placebo 1: random treatment**

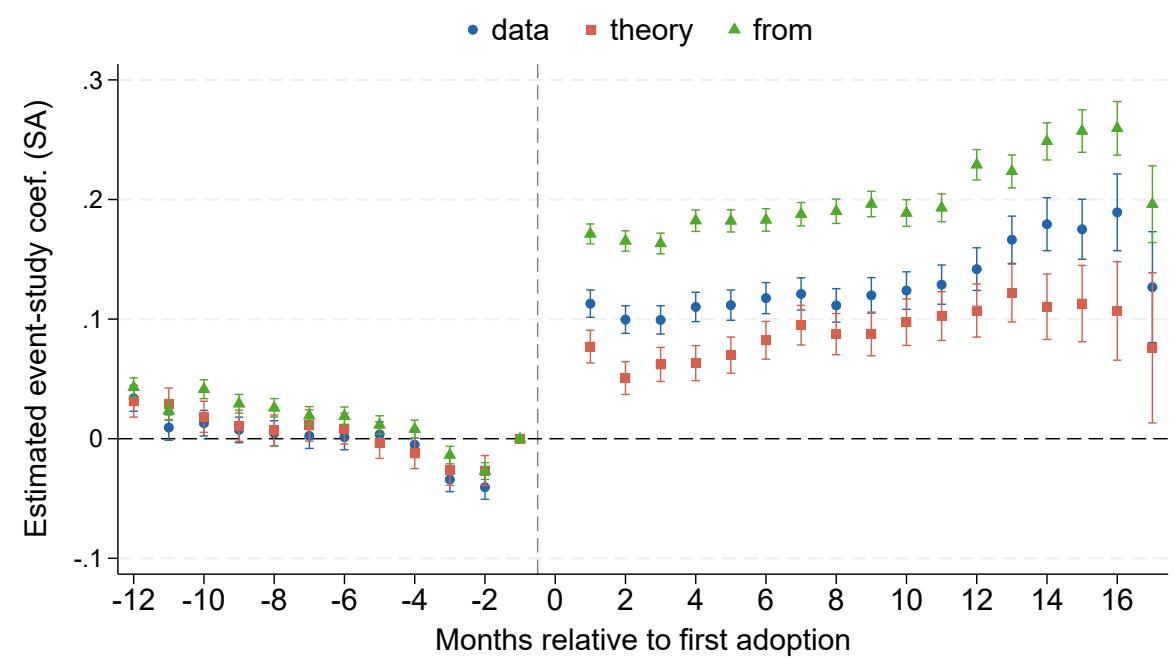

(B) **Placebo 2: keyword treatment**

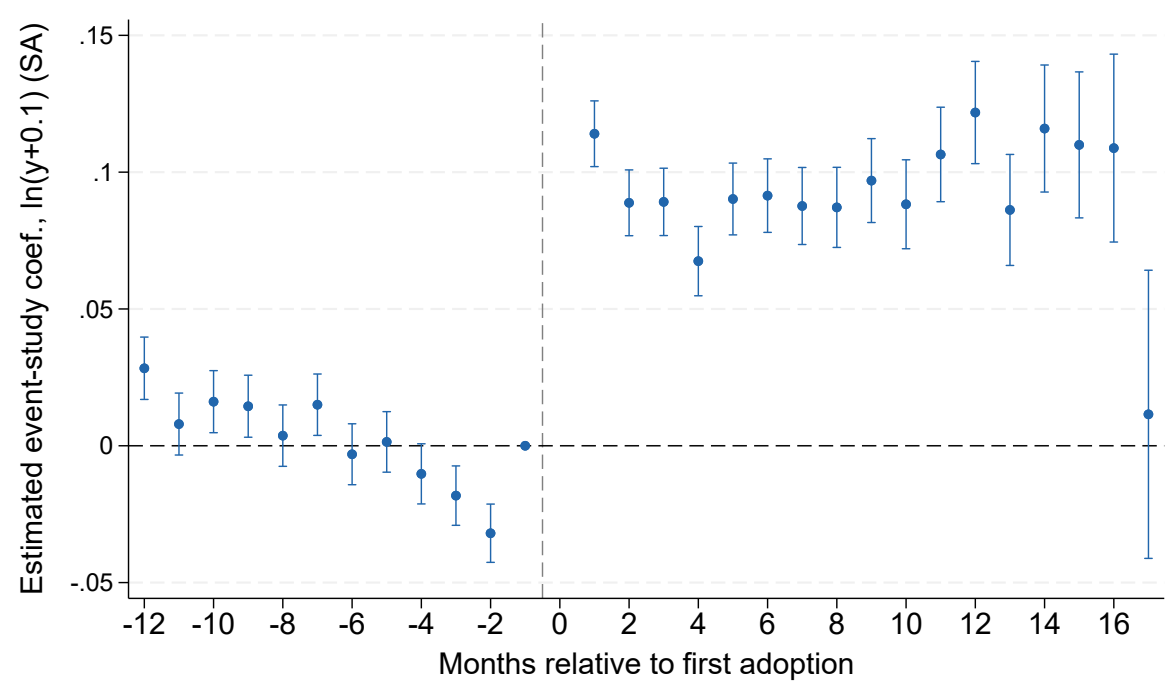

(C) **Placebo 3: pre-ChatGPT period**

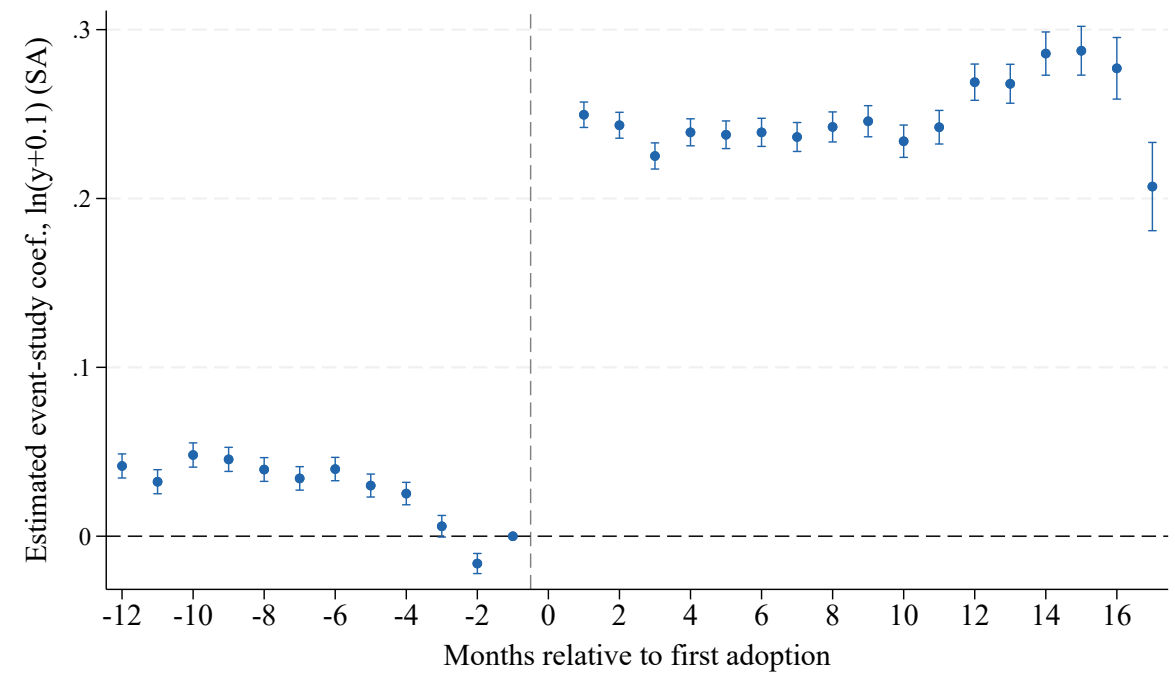

(D) **Placebo 4: inverted treatment**

Figure S13: **Placebo event studies.** All panels report Sun and Abraham's (2021) event-study coefficients, with 95% confidence intervals, of placebo exercises that replace the LLM detector with random paper-level flags (A), neutral keyword flags (B), an earlier observation window (C), and inverted treatment using $\alpha < 0.1$ instead of $\alpha > 0.1$ (D).

6.2.3 with $\ln(0.01 + y)$ as dependent variable

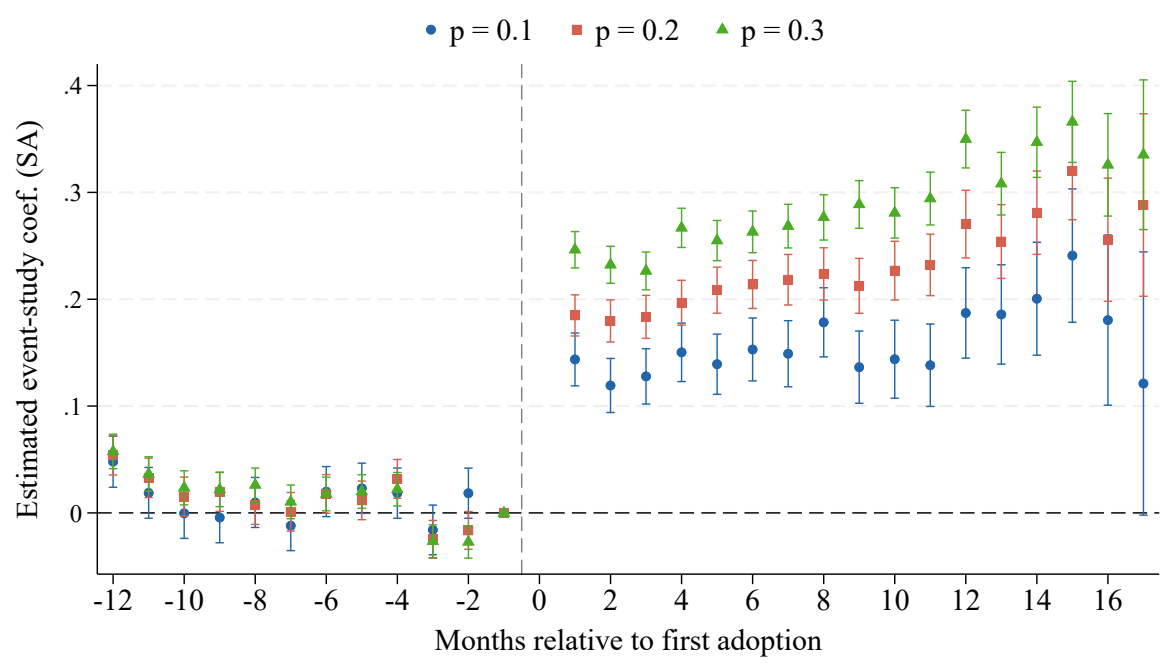

(A) **Placebo 1: random treatment**

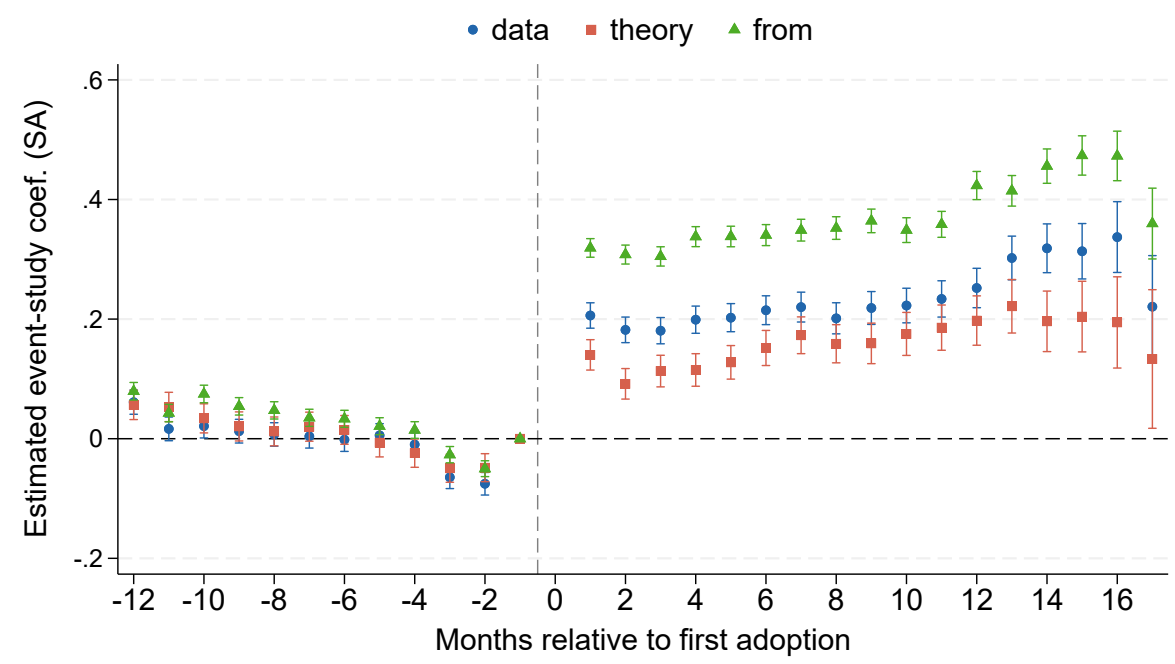

(B) **Placebo 2: keyword treatment**

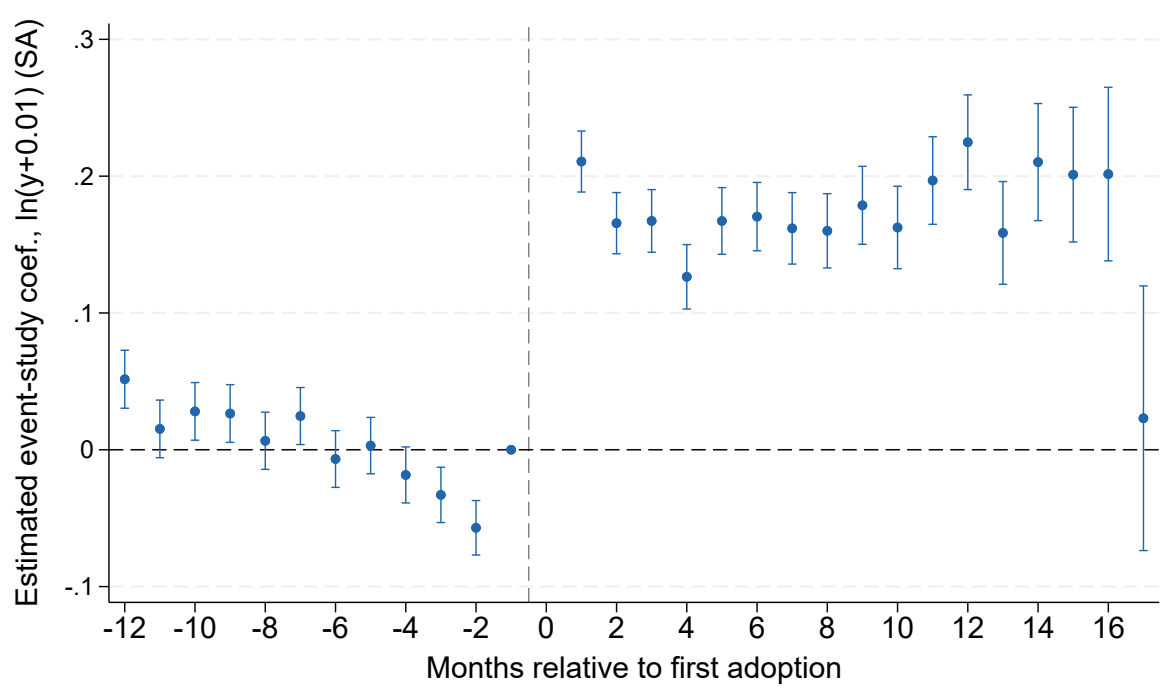

(C) **Placebo 3: pre-ChatGPT period**

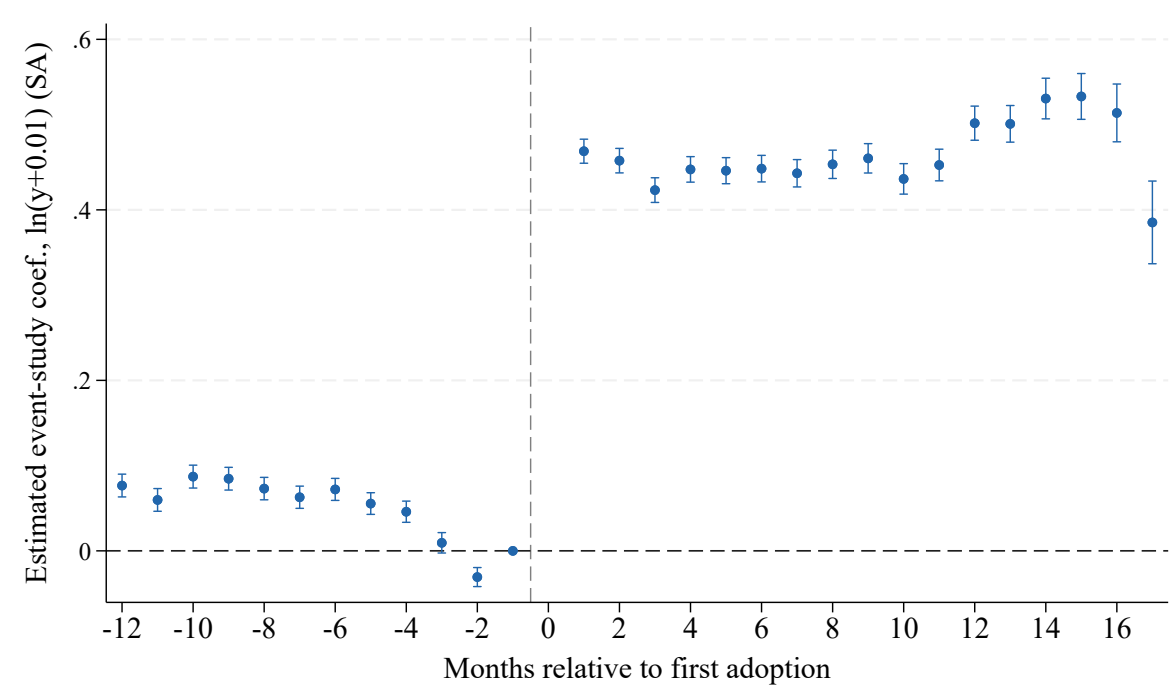

(D) **Placebo 4: inverted treatment**

Figure S14: **Placebo event studies.** All panels report Sun and Abraham's (2021) event-study coefficients, with 95% confidence intervals, of placebo exercises that replace the LLM detector with random paper-level flags (A), neutral keyword flags (B), an earlier observation window (C), and inverted treatment using $\alpha < 0.1$ instead of $\alpha > 0.1$ (D).

6.2.4 with $\ln(x + \sqrt{x^2 + 1})$ (inverse hyperbolic sine) as dependent variable

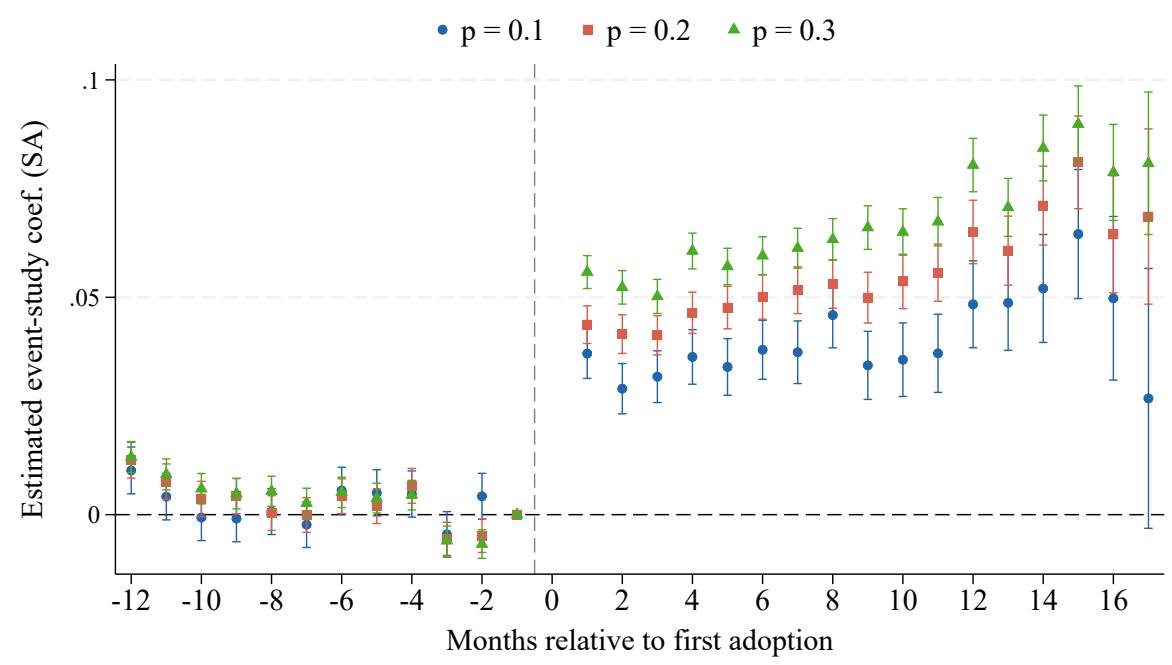

(A) **Placebo 1: random treatment**

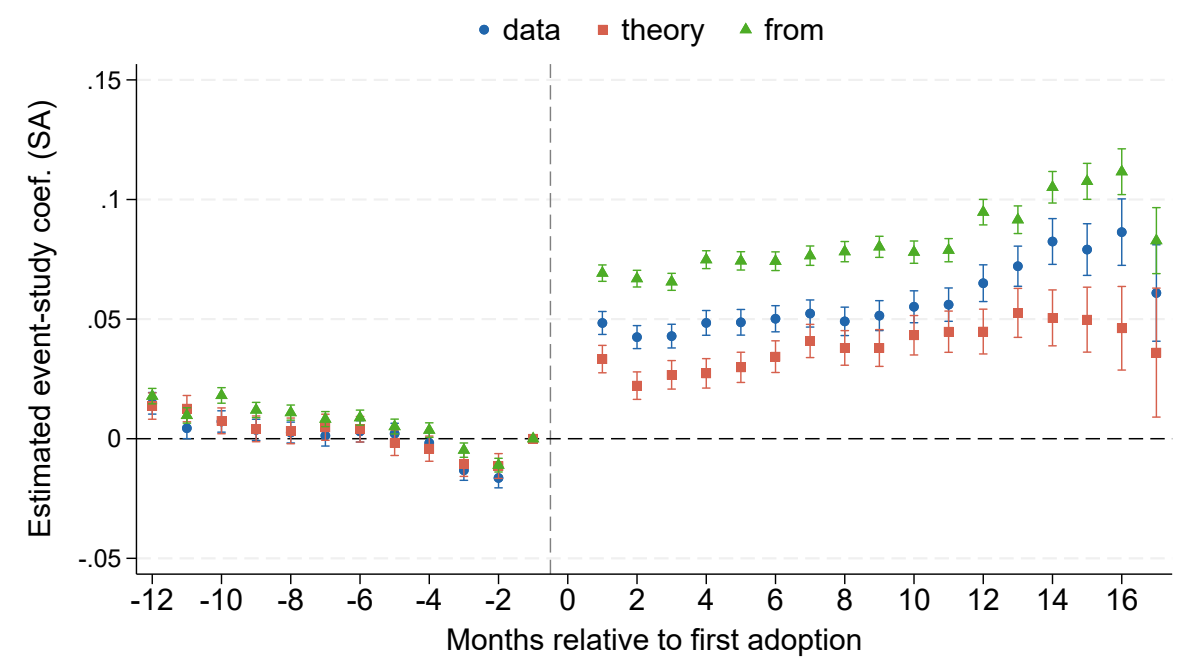

(B) **Placebo 2: keyword treatment**

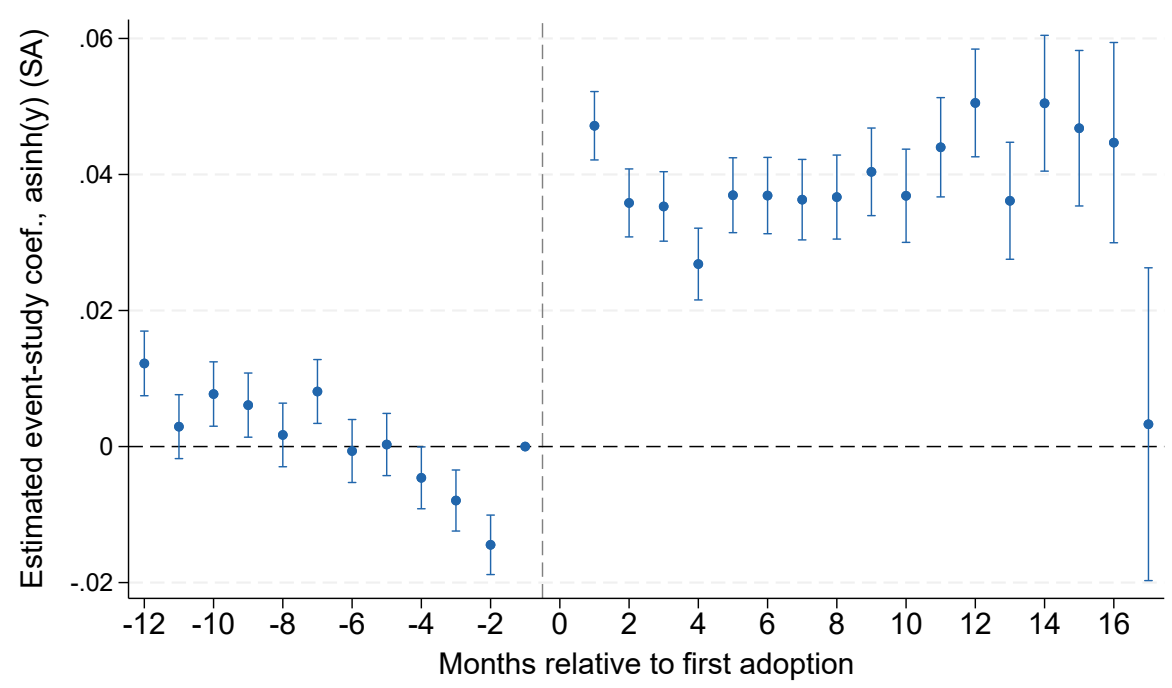

(C) **Placebo 3: pre-ChatGPT period**

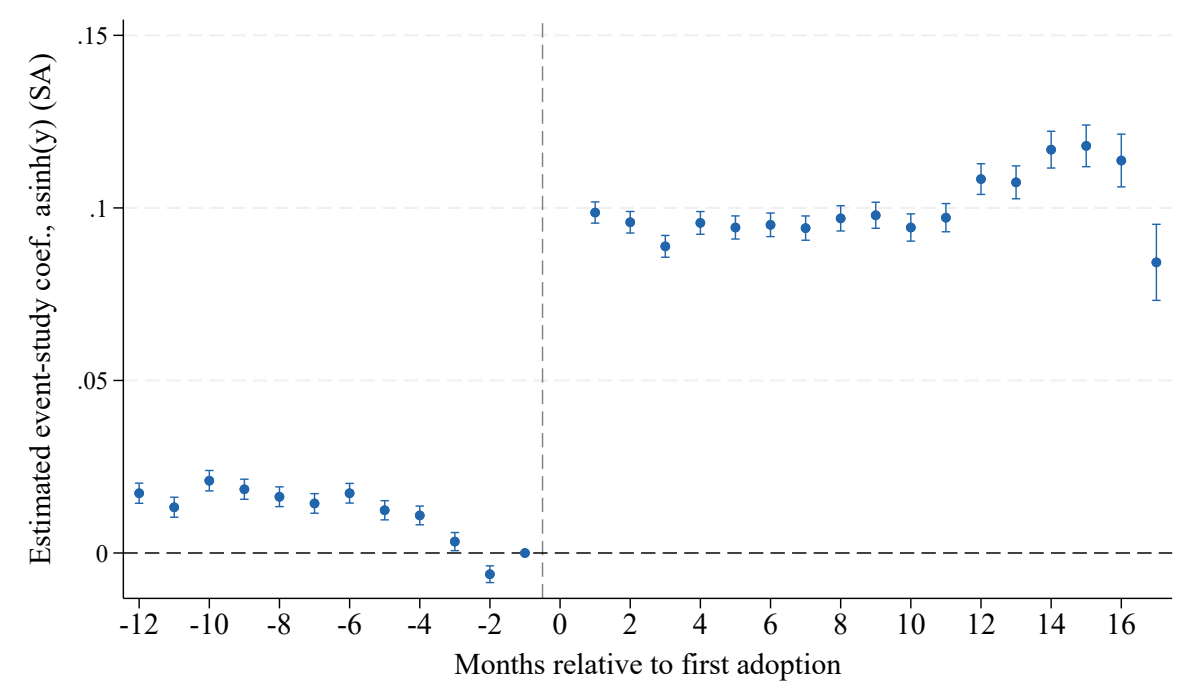

(D) **Placebo 4: inverted treatment**

Figure S15: **Placebo event studies.** All panels report Sun and Abraham's (2021) event-study coefficients, with 95% confidence intervals, of placebo exercises that replace the LLM detector with random paper-level flags (A), neutral keyword flags (B), an earlier observation window (C), and inverted treatment using $\alpha < 0.1$ instead of $\alpha > 0.1$ (D).

7 Random treatment placebo with event-time balanced windows

Figure S16 reports the random treatment placebo results using event-time balanced windows, ensuring that the comparison between treated and control authors covers the same range of relative periods.

It does not show the first-treatment-month coefficients ($k = 0$), while Figure S33 in section 10.4 below does.

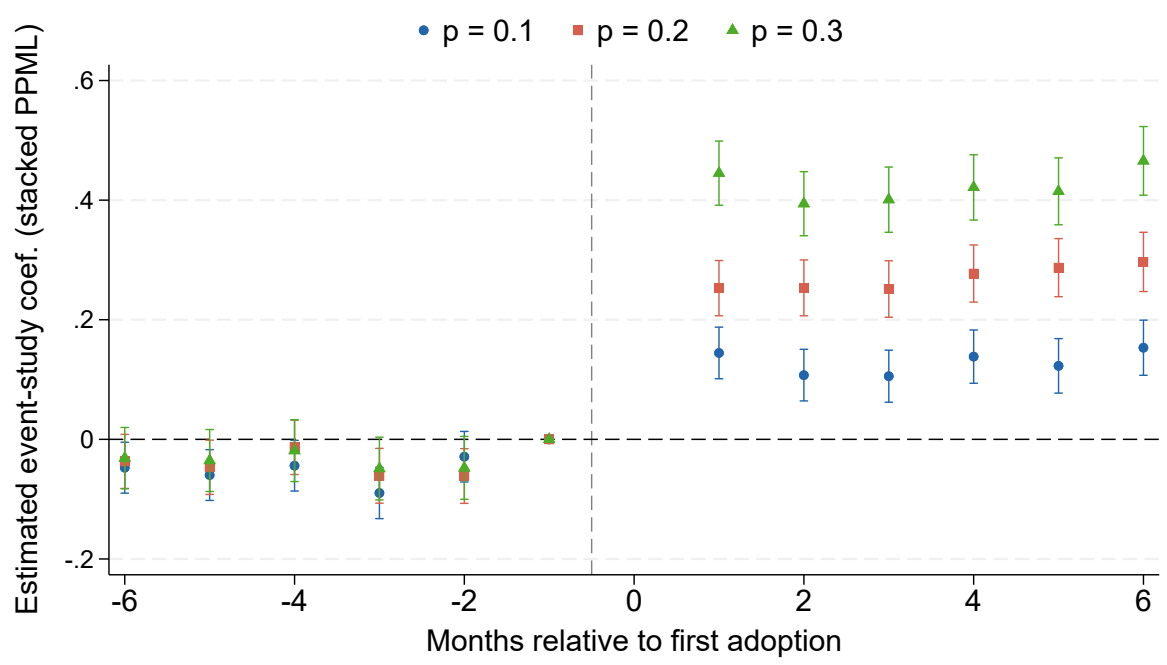

(A) **Balanced window:** $[-6; +6]$

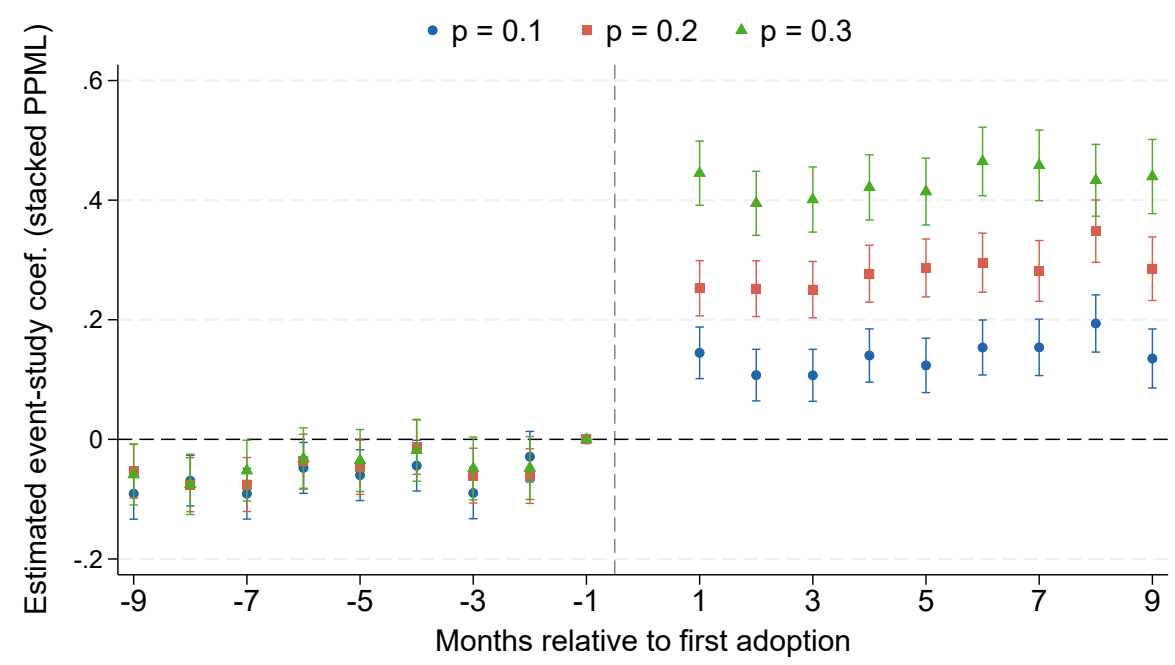

(B) **Balanced window:** $[-9; +9]$

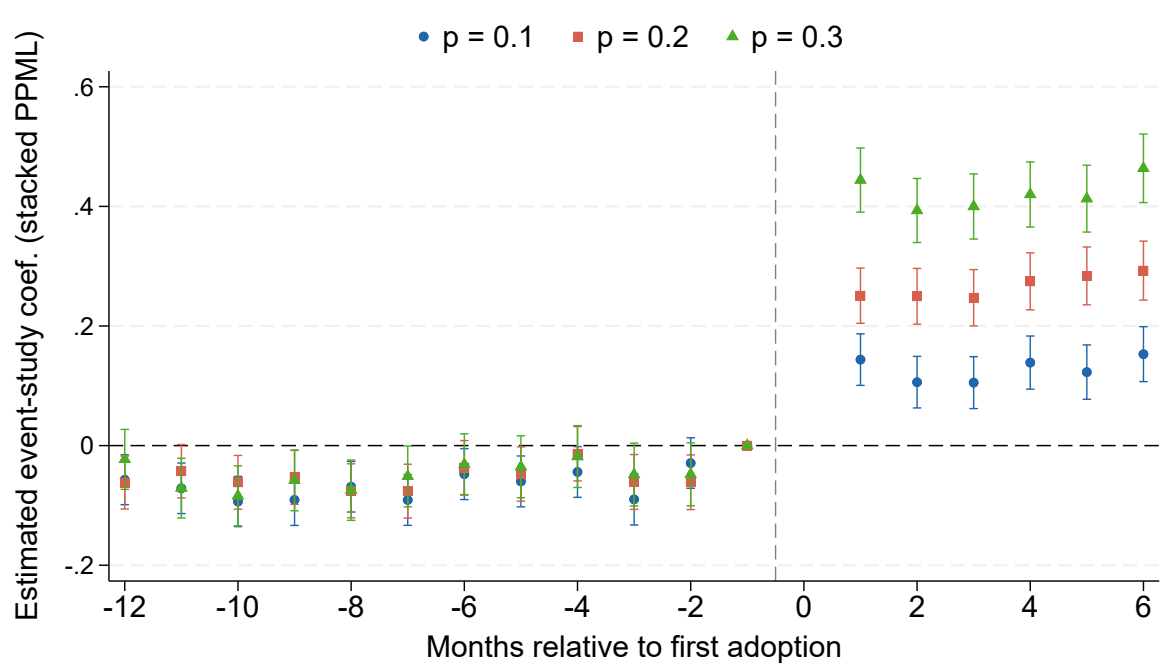

(C) **Balanced window:** $[-12; +6]$

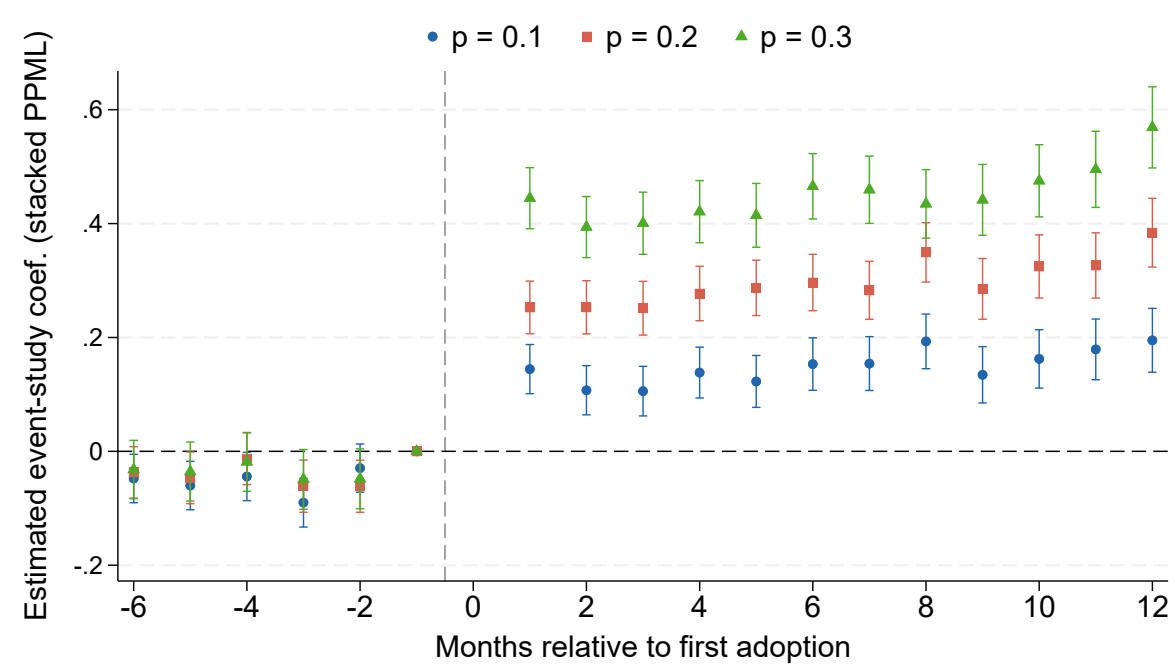

(D) **Balanced window:** $[-6; +12]$

Figure S16: **Random placebo event studies.** All panels report stacked-PPML event-study coefficients, with 95% confidence intervals, of the random placebo exercise, with different event-time balanced windows.

8 Placebo exercises of Figure 1 in stacked-PPML event study with alternative control group timing

Figure S17 reports stacked-PPML event-study estimates when applying alternative methods for defining the timing of the control group in the stacked design. Rather than assigning control authors a pseudo-treatment date equal to the treated cohort date, each control author is assigned a pseudo-date drawn from the empirical distribution of treated authors' adoption dates.

Figure S17 does not show the first-treatment-month coefficients ($k = 0$), while Figure S34 in section 10.5 below does.

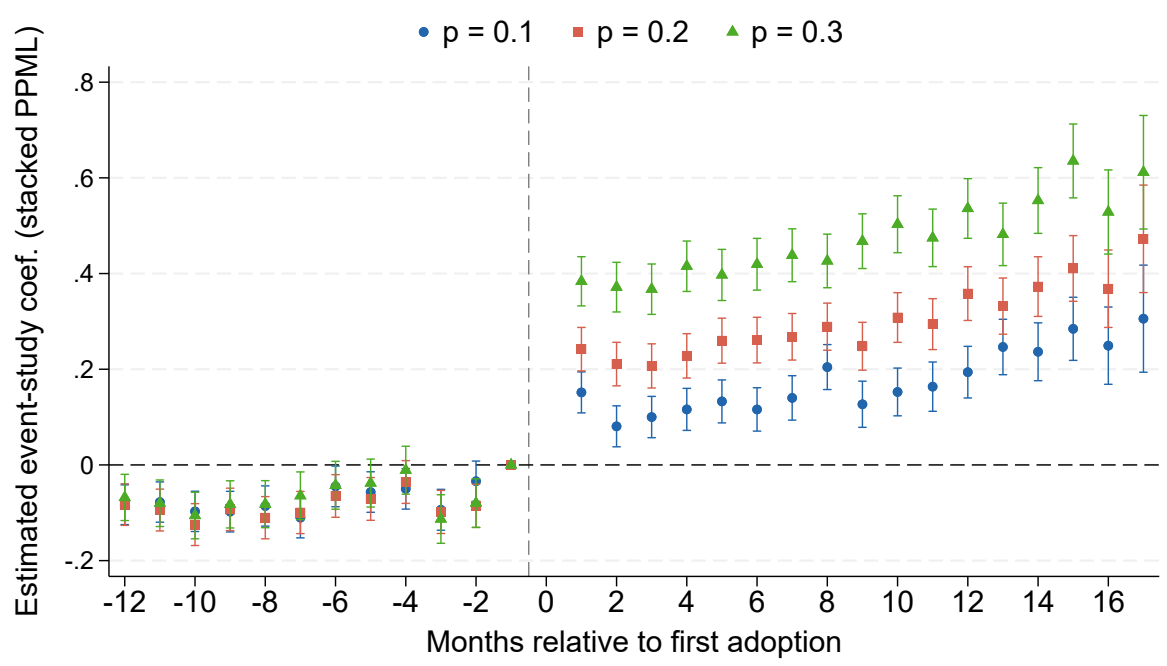

(A) **Placebo 1: random treatment**

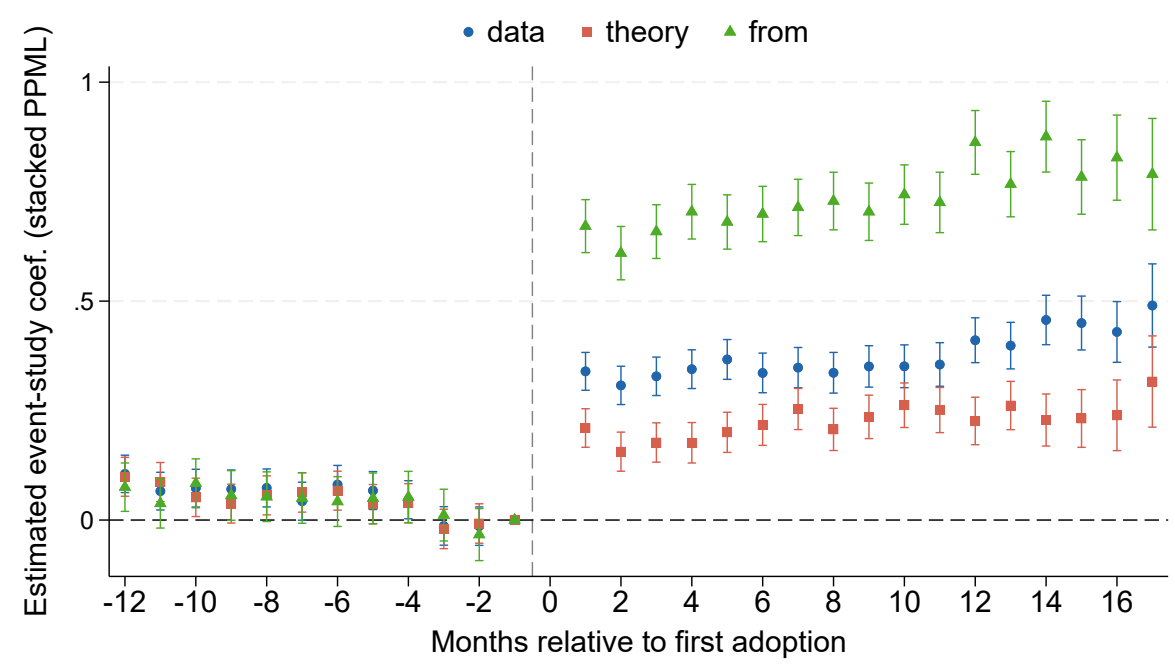

(B) **Placebo 2: keyword treatment**

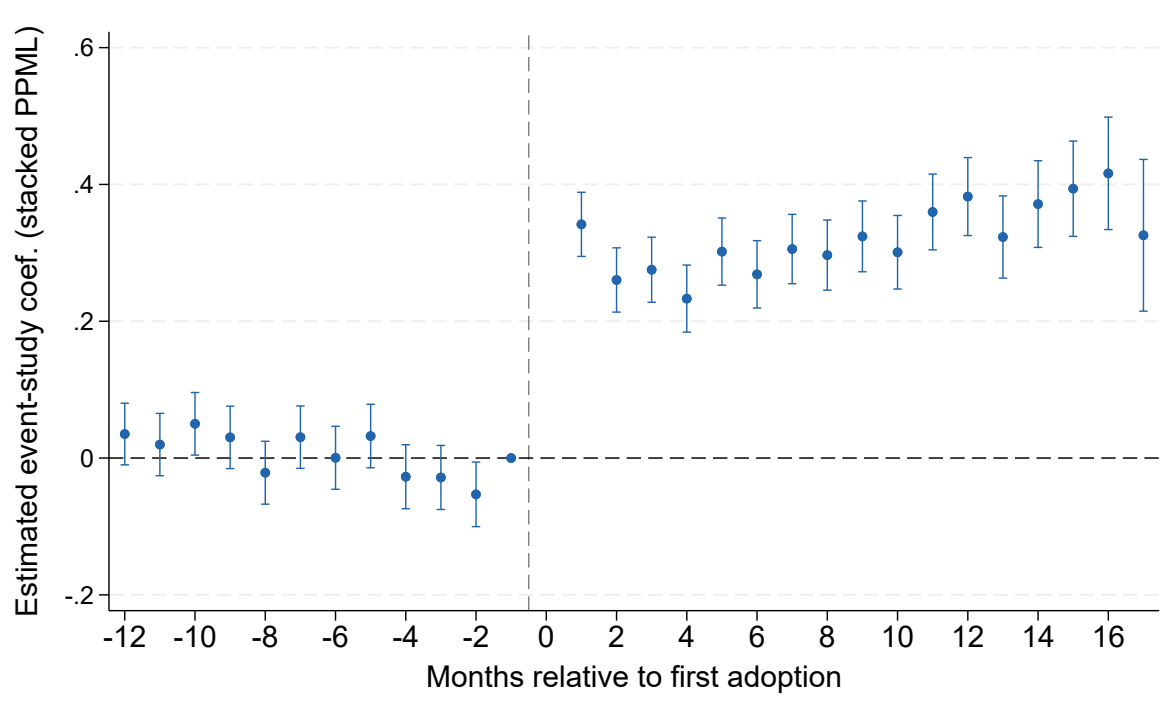

(C) **Placebo 3: pre-ChatGPT period**

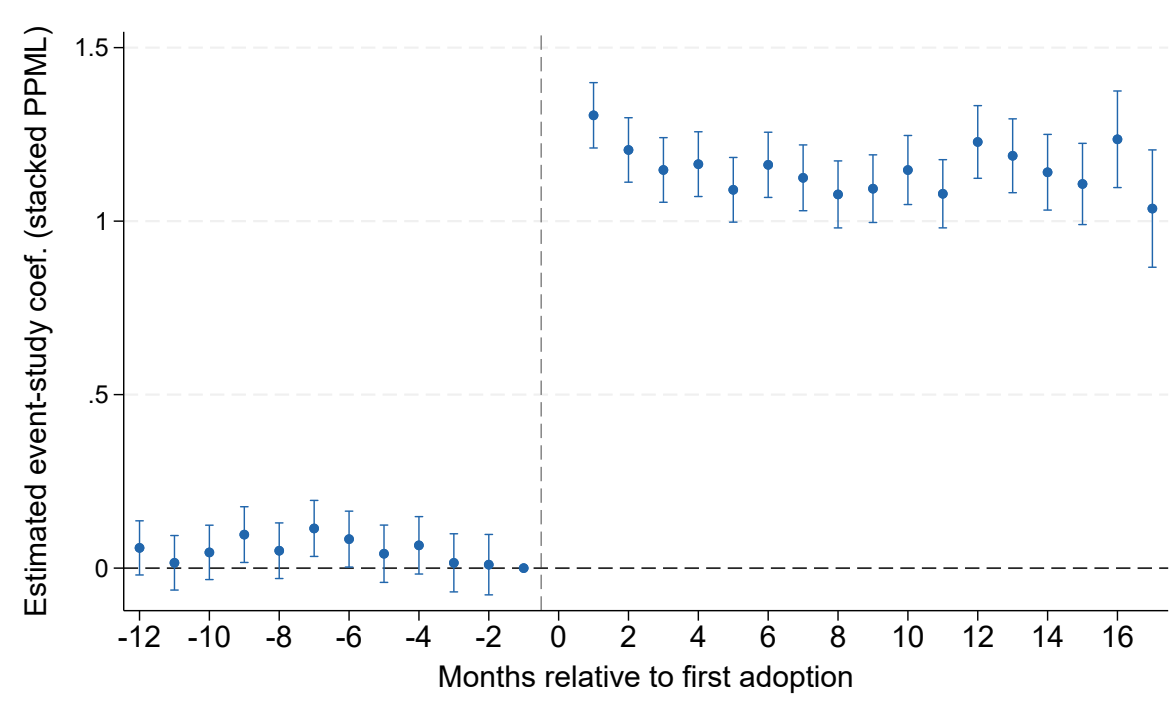

(D) **Placebo 4: inverted treatment**

Figure S17: **Placebo event studies.** All panels report stacked-PPML event-study coefficients, with 95% confidence intervals, of placebo exercises that replace the LLM detector with random paper-level flags (A), neutral keyword flags (B), an earlier observation window (C), and inverted treatment using $\alpha < 0.1$ instead of $\alpha > 0.1$ (D).

9 Estimations excluding observations corresponding to the first treatment month

9.1 Stacked-PPML event study

Figures S18 and S19 report stacked-PPML event-study estimates when observations corresponding to the first treatment month have been dropped prior to the estimation.

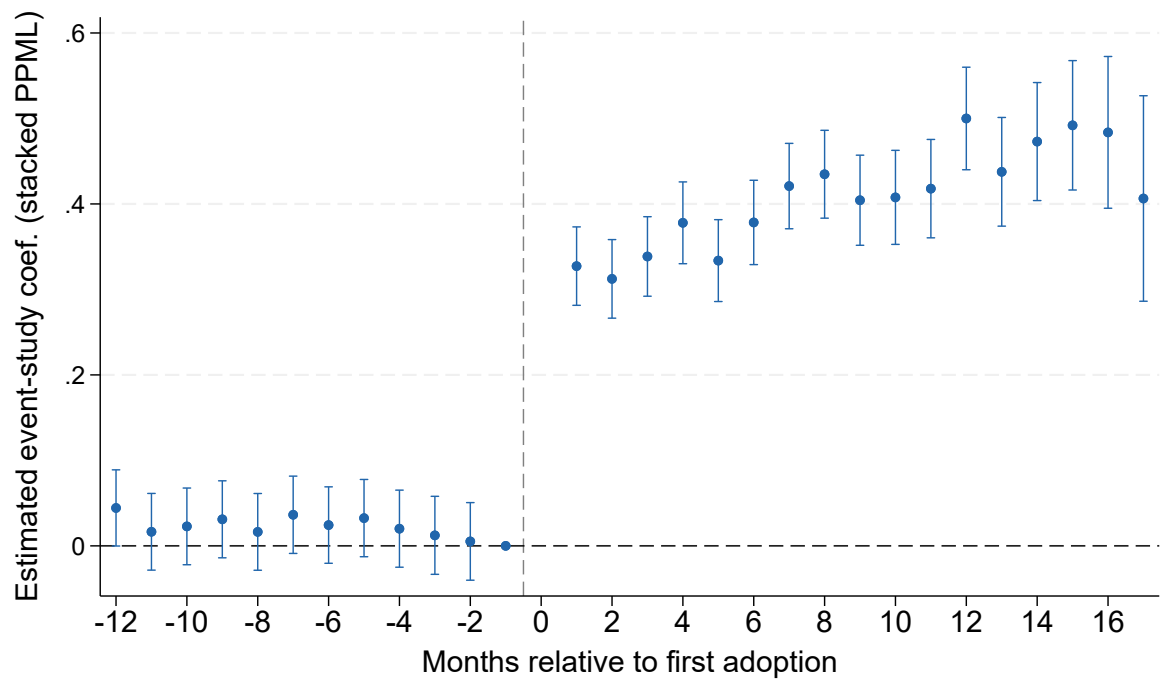

(A) Kusumegi et al. [1]' data

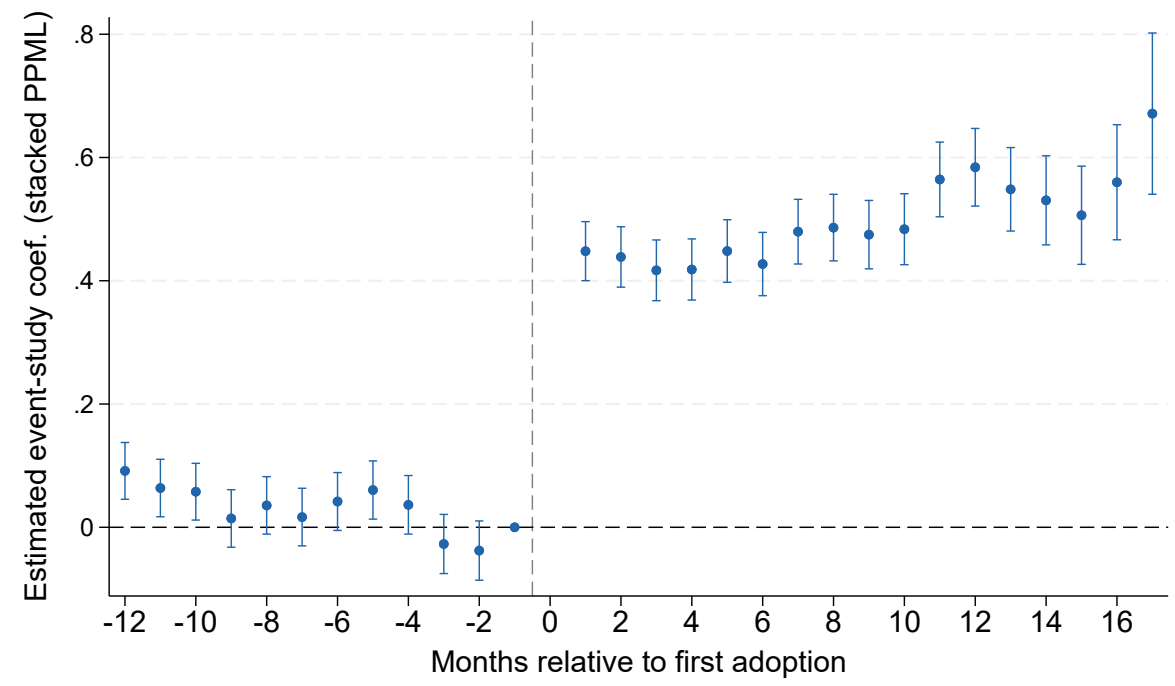

(B) Replication

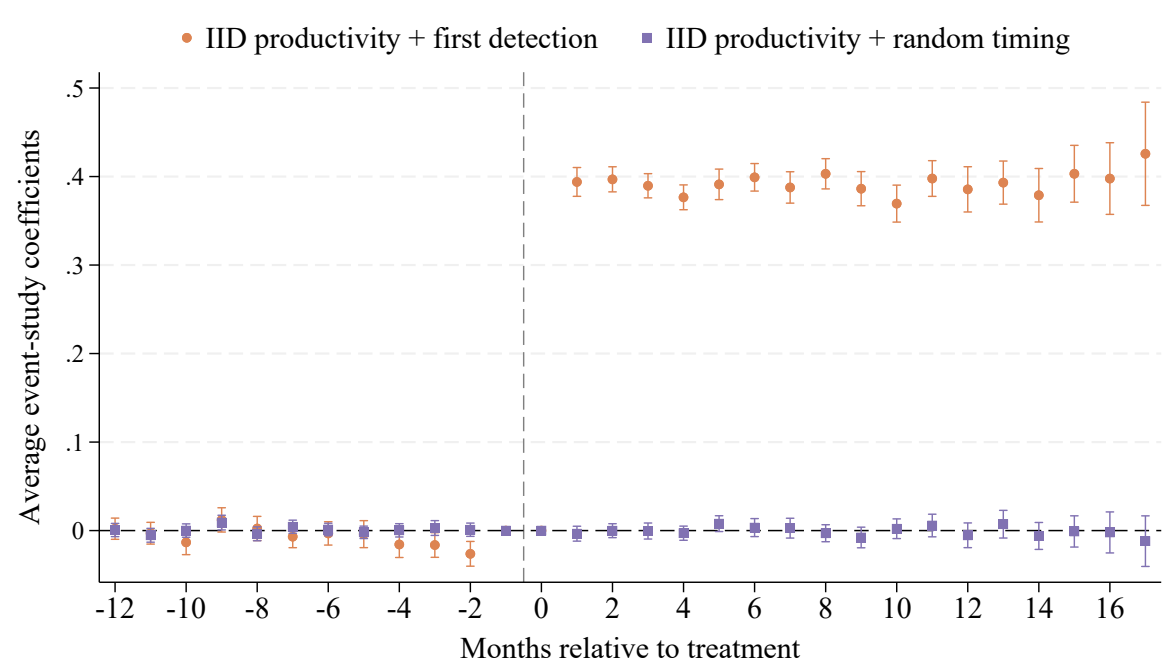

(C) Simulations

Figure S18: **Replications and simulations.** Panels (A) and (B) report stacked-PPML event-study coefficients, with 95% confidence intervals. Panel (C) reports the averages of stacked-PPML event-study coefficients across 100 simulations with 10,000 authors and i.i.d. productivity (flag probability p=0.3 in the first-detection scenario).

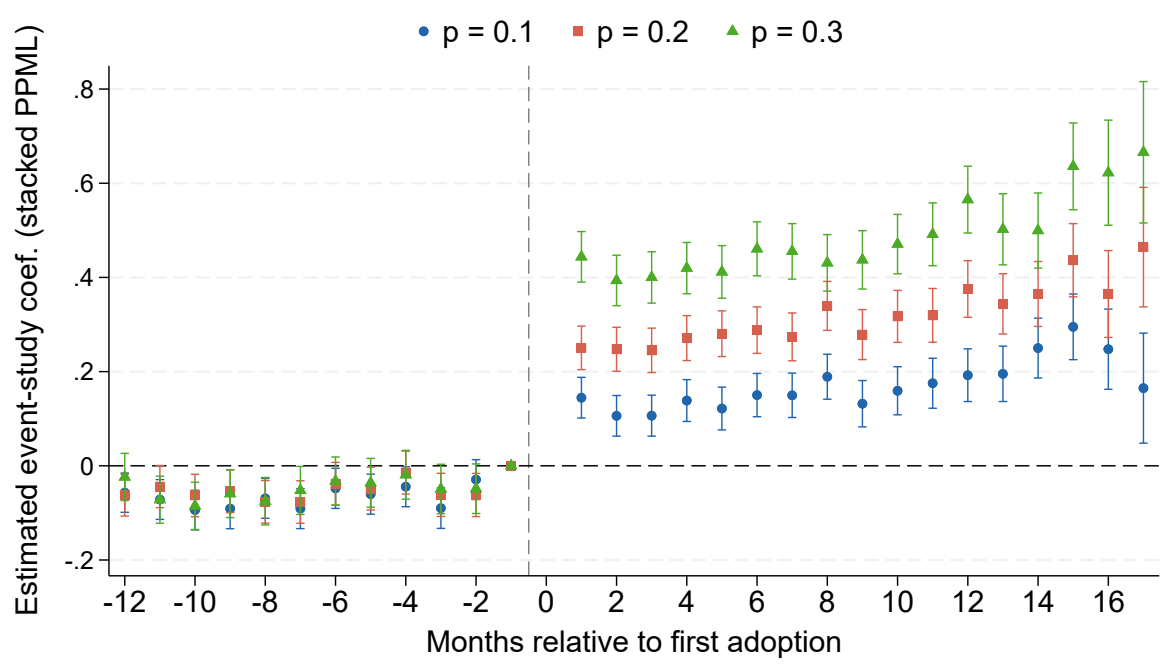

(A) **Placebo 1: random treatment**

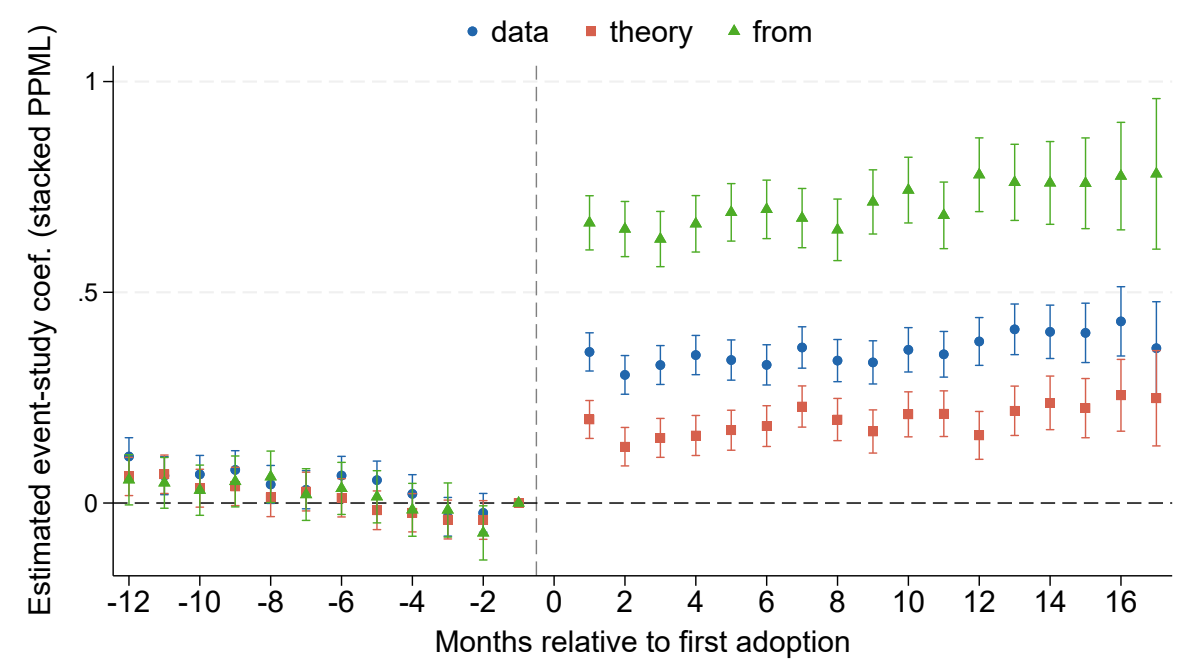

(B) **Placebo 2: keyword treatment**

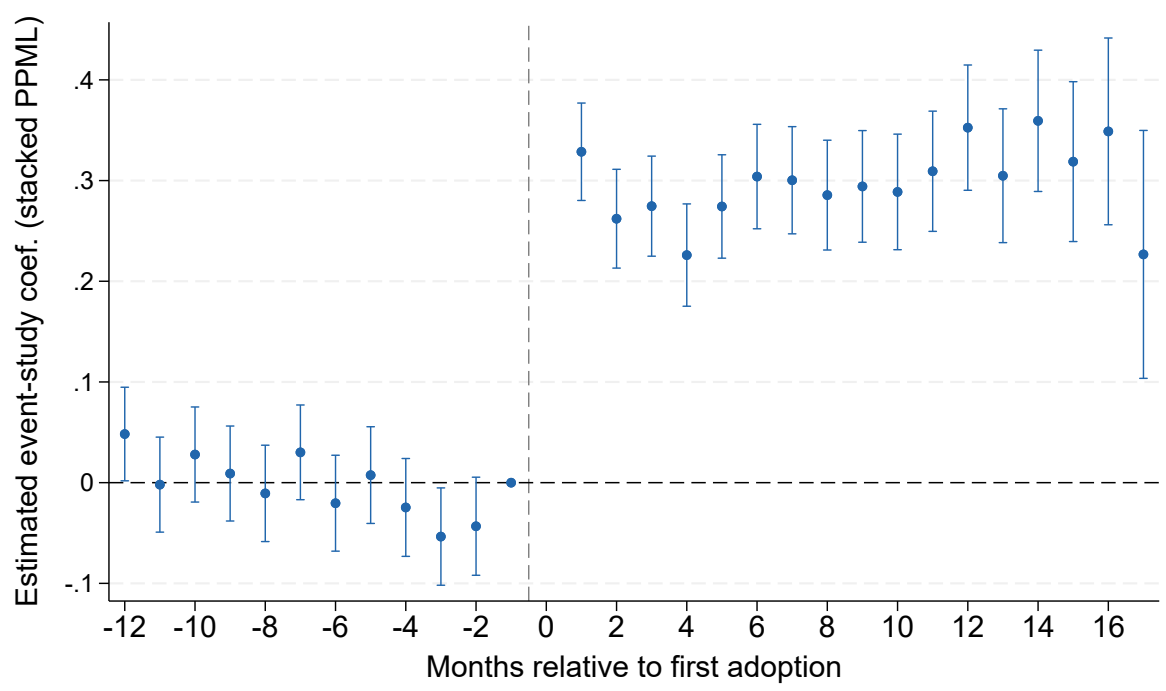

(C) **Placebo 3: pre-ChatGPT period**

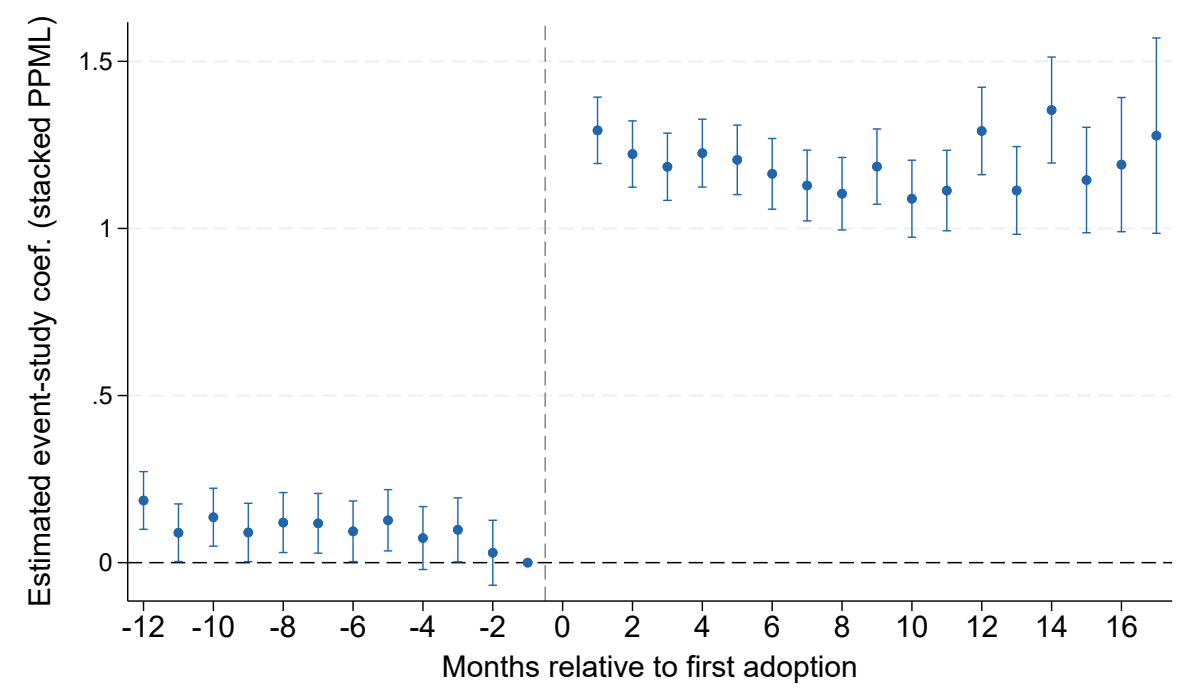

(D) **Placebo 4: inverted treatment**

Figure S19: **Placebo event studies.** All panels report stacked-PPML event-study coefficients, with 95% confidence intervals, of placebo exercises that replace the LLM detector with random paper-level flags (A), neutral keyword flags (B), an earlier observation window (C), and inverted treatment using $\alpha < 0.1$ instead of $\alpha > 0.1$ (D).

9.2 Two-by-two DiD estimators (corresponding to Figure 1E)

Table S3 reports the two-by-two difference-in-differences estimates underlying Figure 1E, covering both the main specification and all placebo variants, together with their corresponding semi-elasticities.

Table S3: (stacked-PPML) **two-by-two DiD** coefficients and their corresponding semi-elasticity

	Replications		Placebos							
	Data:		Random-flag			Pre-	Keywords			Inverted
	original	replication	10%	20%	30%	Chat-GPT	data	theory	from	
	(1)	(2)	(3)	(4)	(5)	(6)	(7)	(8)	(9)	(10)
Coefficient	0.361	0.432	0.231	0.361	0.520	0.293	0.295	0.169	0.666	1.087
	(0.007)	(0.007)	(0.007)	(0.007)	(0.008)	(0.007)	(0.007)	(0.007)	(0.009)	(0.014)
Eff. in %	43.5	54.1	25.9	43.4	68.3	34.0	34.3	18.4	94.7	196.7

Standard errors are clustered at the individual level. All coefficients are significant at the 1% level.
Eff. in % is calculated as $(exp(coefficient) - 1) \times 100$.

10 Event studies showing the first-treatment-month coefficient ($k = 0$)

10.1 Placebo exercises of Figure 1 in stacked-PPML event study

Figure S20 reports stacked-PPML event-study coefficients, with 95% confidence intervals, for each placebo specification. Coefficients include the first-treatment month ($k = 0$), which is mechanically positive when treatment is defined by the first occurrence of the outcome.

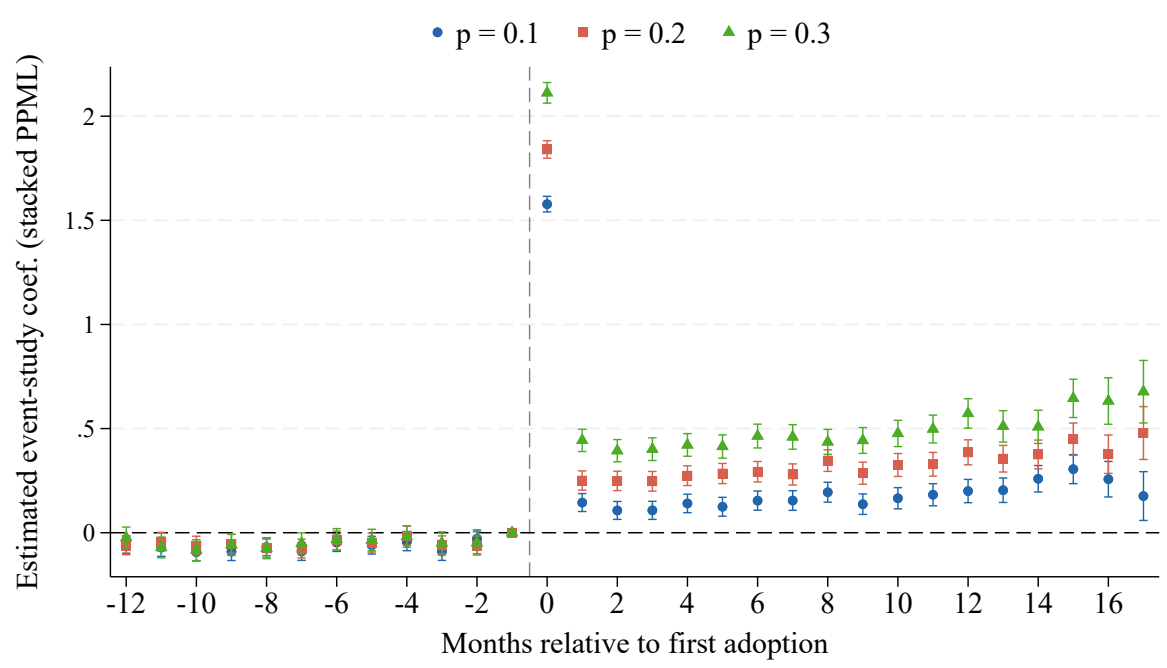

(A) **Placebo 1: random treatment**

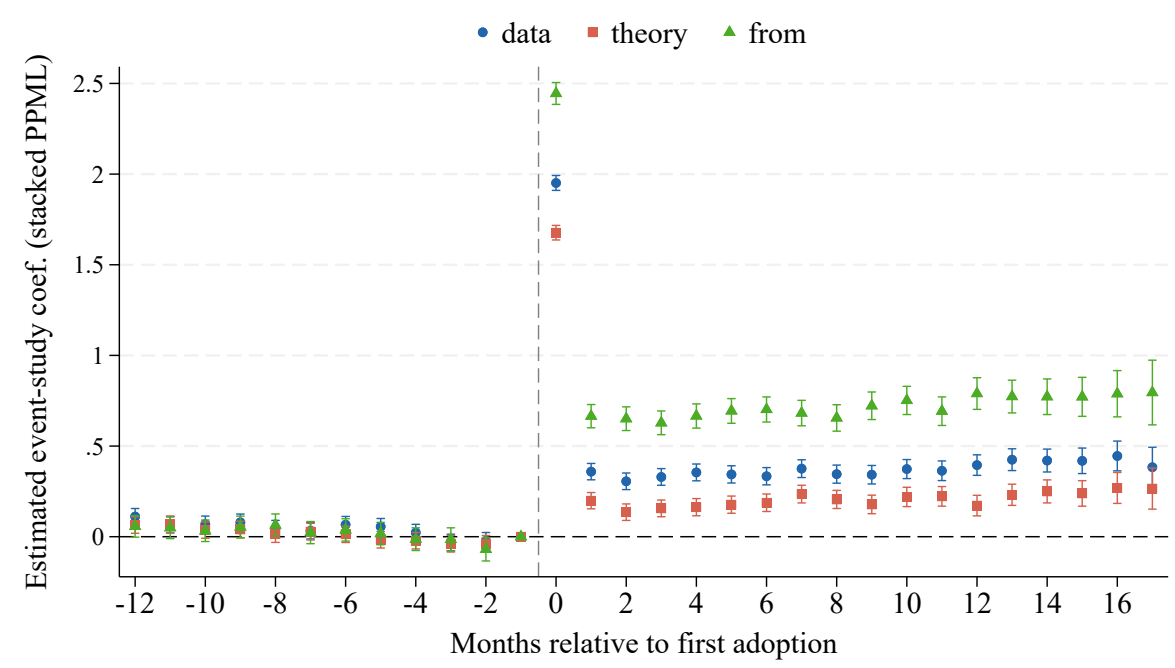

(B) **Placebo 2: keyword treatment**

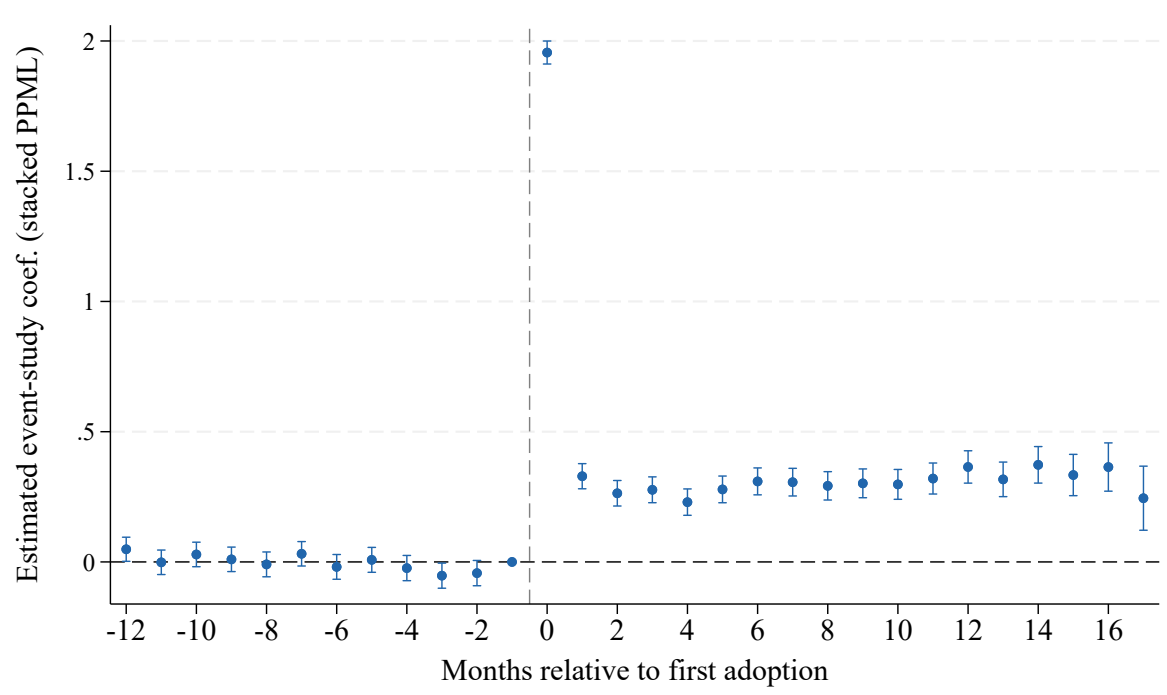

(C) **Placebo 3: pre-ChatGPT period**

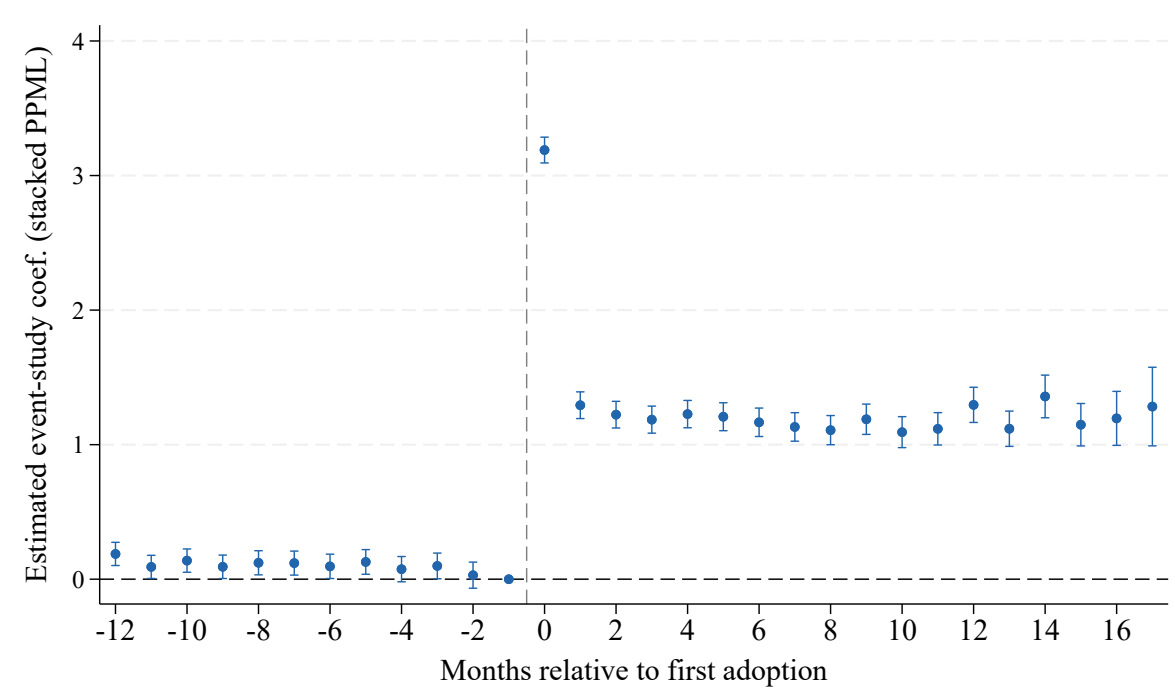

(D) **Placebo 4: inverted treatment**

Figure S20: **Placebo event studies.** All panels report stacked-PPML event-study coefficients, with 95% confidence intervals, of placebo exercises that replace the LLM detector with random paper-level flags (A), neutral keyword flags (B), an earlier observation window (C), and inverted treatment using $\alpha < 0.1$ instead of $\alpha > 0.1$ (D).

10.2 Placebo exercises of Figure 1 in stacked-OLS event study

This section replicates the placebo exercises of Figure 1 using a stacked-OLS estimator instead of the Poisson pseudo-maximum-likelihood (PPML) used in the main text, confirming that the results are robust to the choice of estimator.

10.2.1 With the dependent variable in levels (y = number of papers)

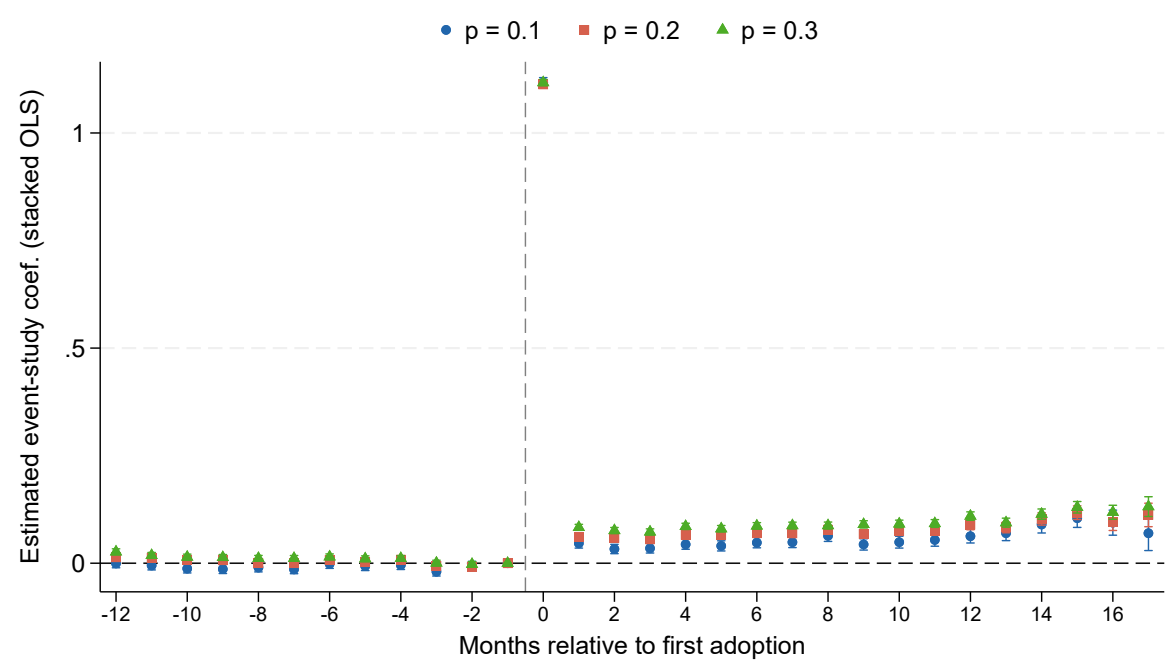

(A) **Placebo 1: random treatment**

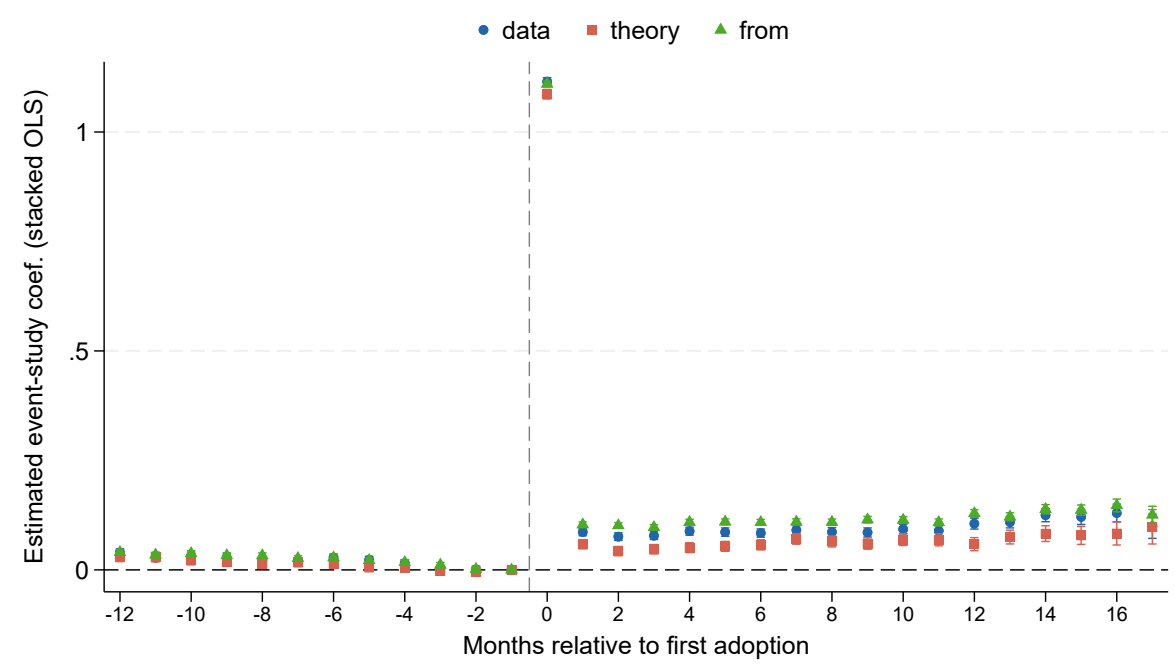

(B) **Placebo 2: keyword treatment**

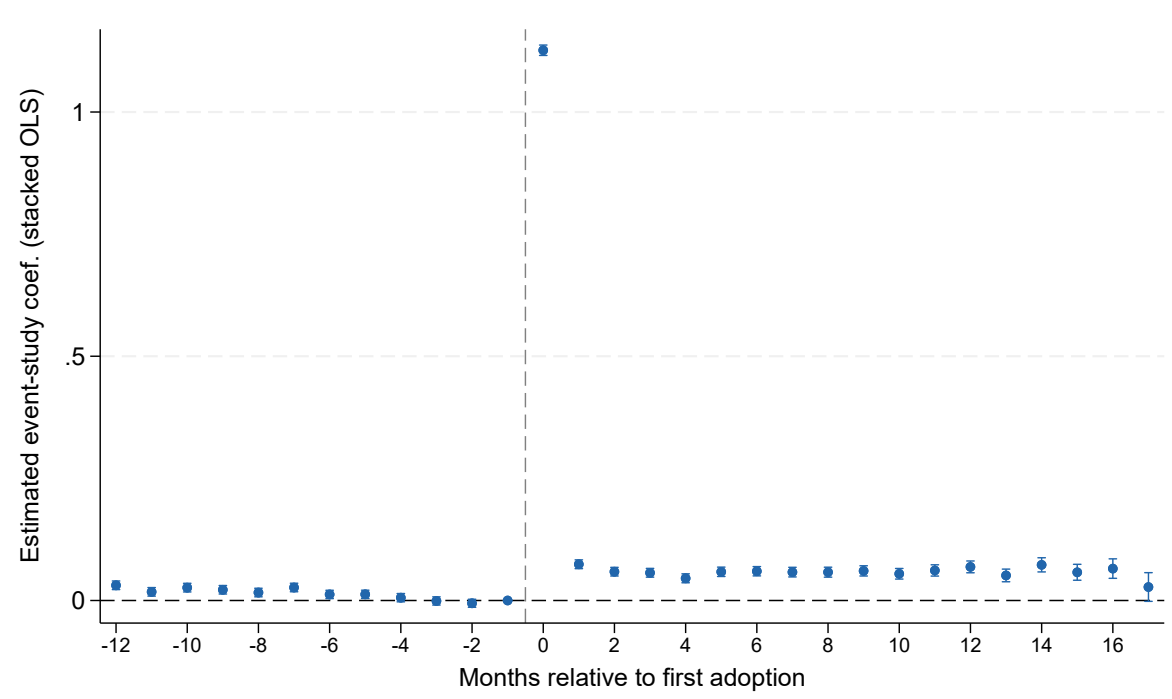

(C) **Placebo 3: pre-ChatGPT period**

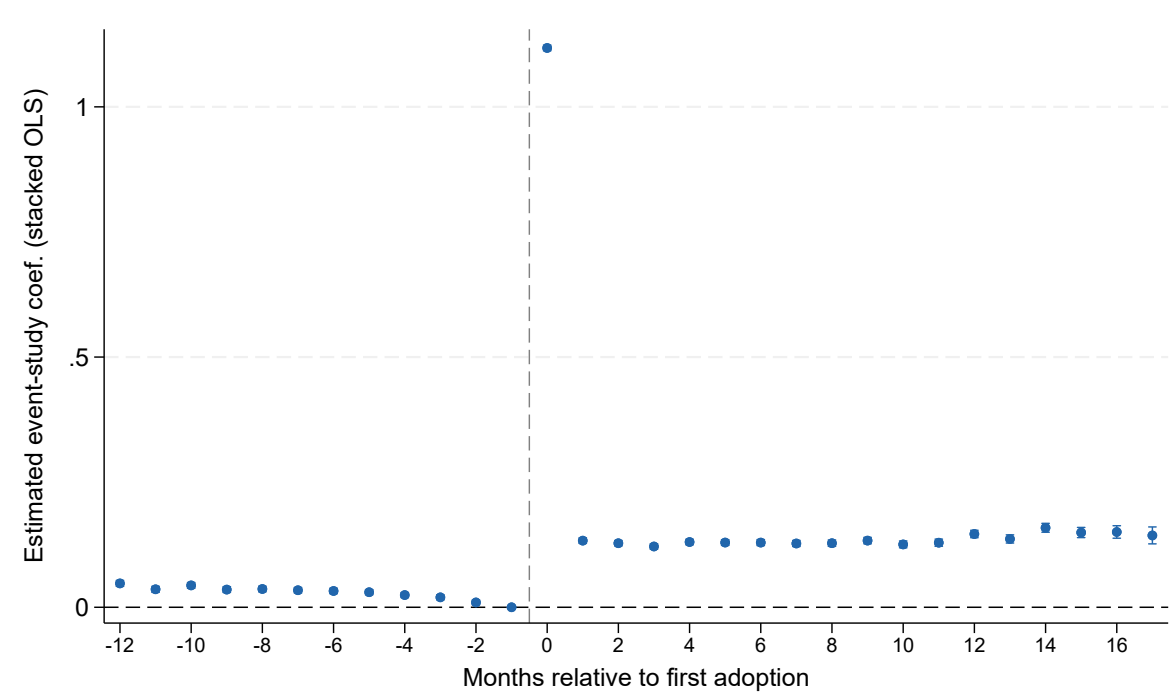

(D) **Placebo 4: inverted treatment**

Figure S21: **Placebo event studies.** All panels report stacked-OLS event-study coefficients, with 95% confidence intervals, of placebo exercises that replace the LLM detector with random paper-level flags (A), neutral keyword flags (B), an earlier observation window (C), and inverted treatment using $\alpha < 0.1$ instead of $\alpha > 0.1$ (D).

10.2.2 with $\ln(0.1 + y)$ as dependent variable

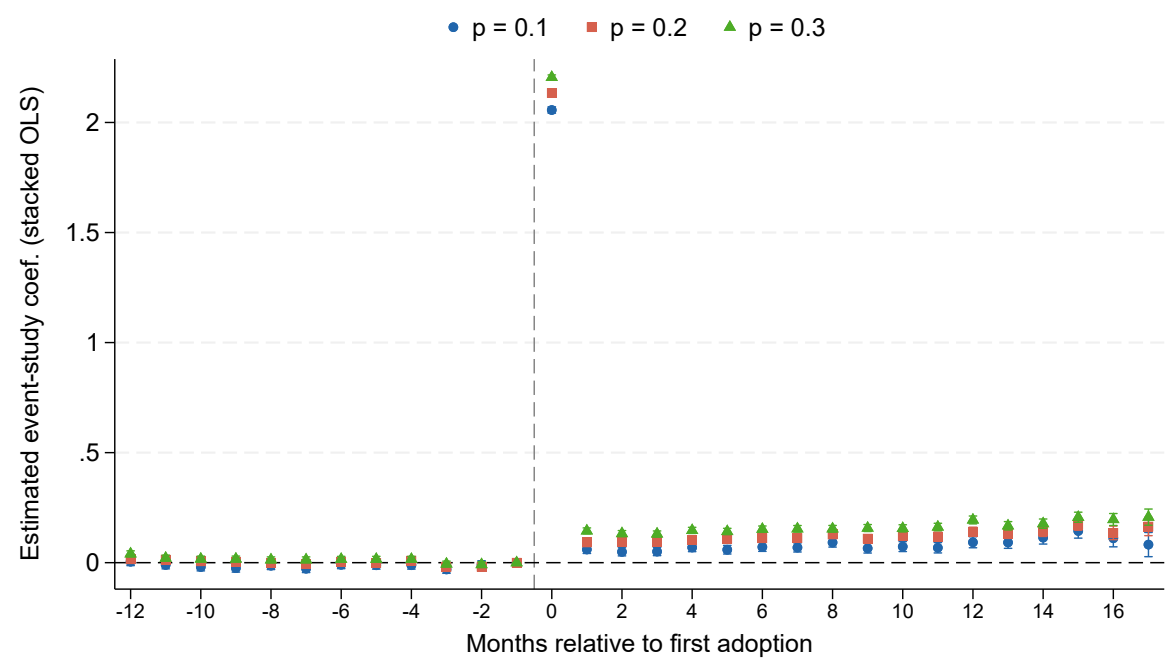

(A) **Placebo 1: random treatment**

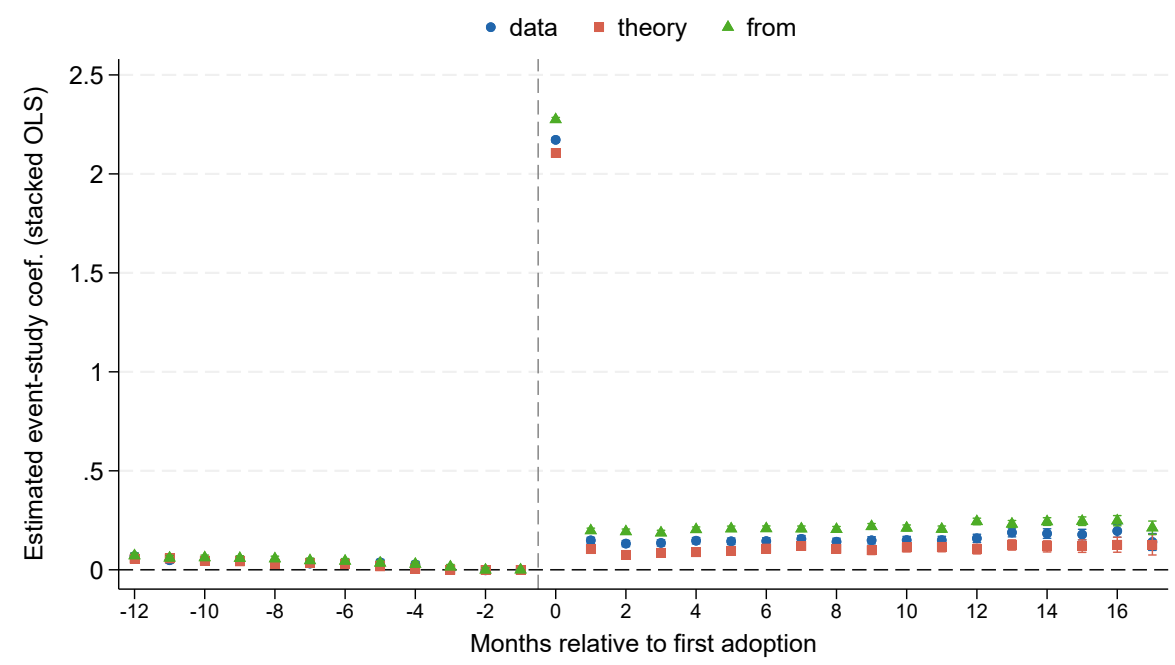

(B) **Placebo 2: keyword treatment**

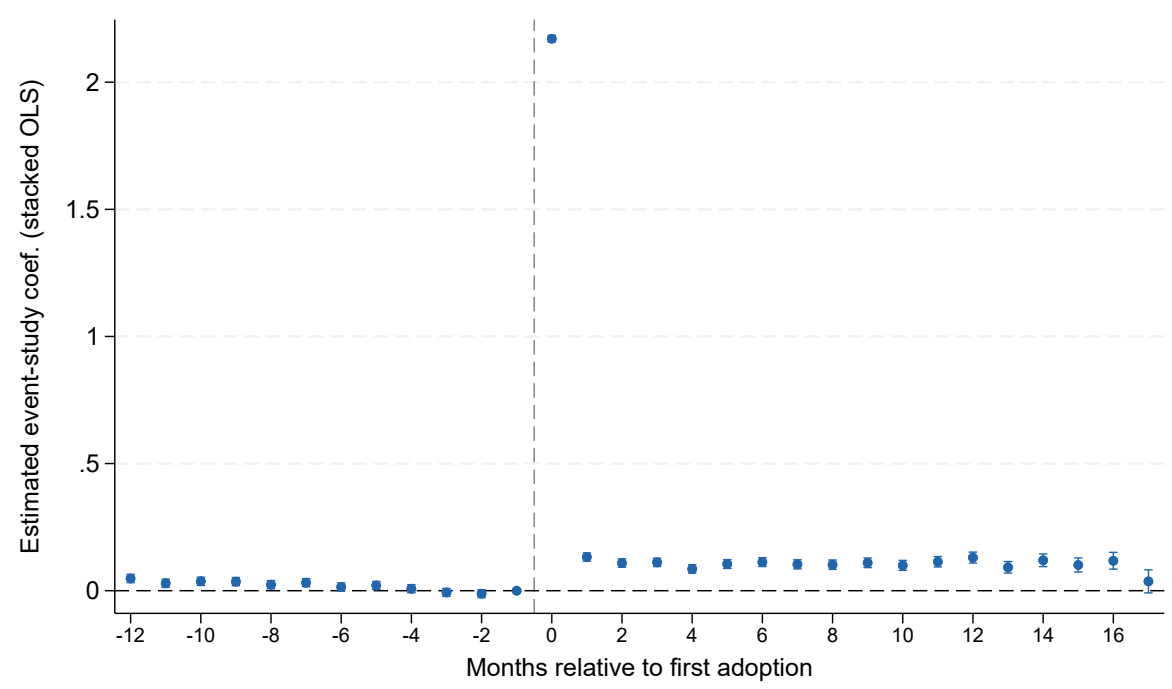

(C) **Placebo 3: pre-ChatGPT period**

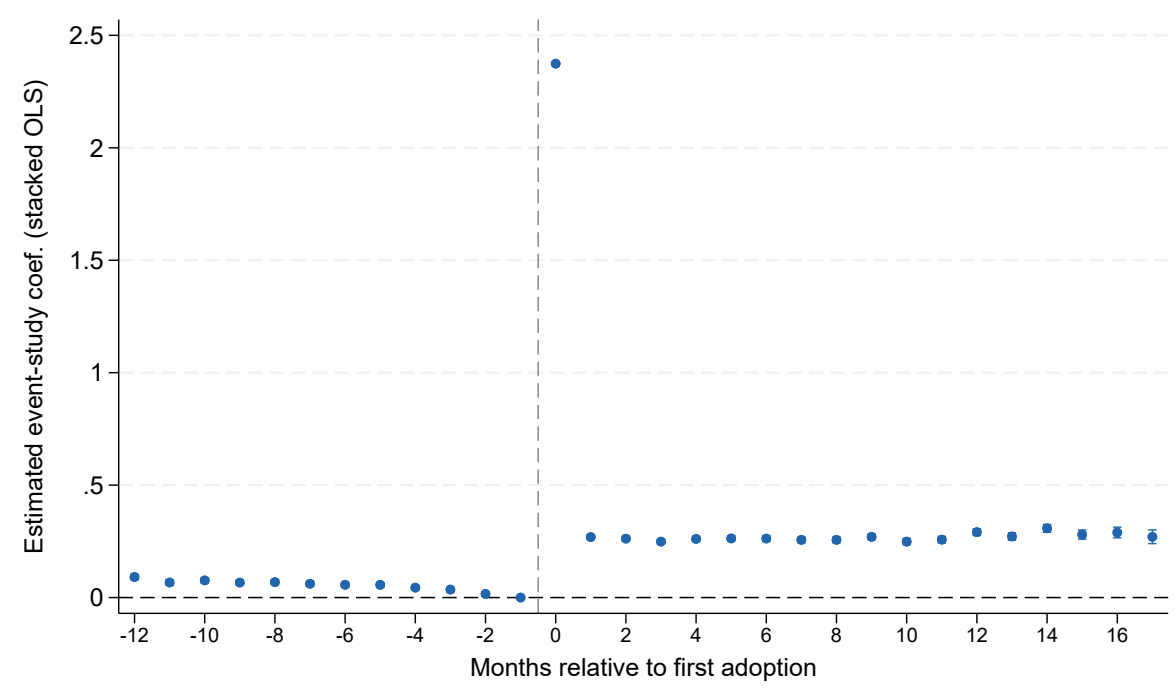

(D) **Placebo 4: inverted treatment**

Figure S22: **Placebo event studies.** All panels report stacked-OLS event-study coefficients, with 95% confidence intervals, of placebo exercises that replace the LLM detector with random paper-level flags (A), neutral keyword flags (B), an earlier observation window (C), and inverted treatment using $\alpha < 0.1$ instead of $\alpha > 0.1$ (D).

10.2.3 with $\ln(0.01 + y)$ as dependent variable

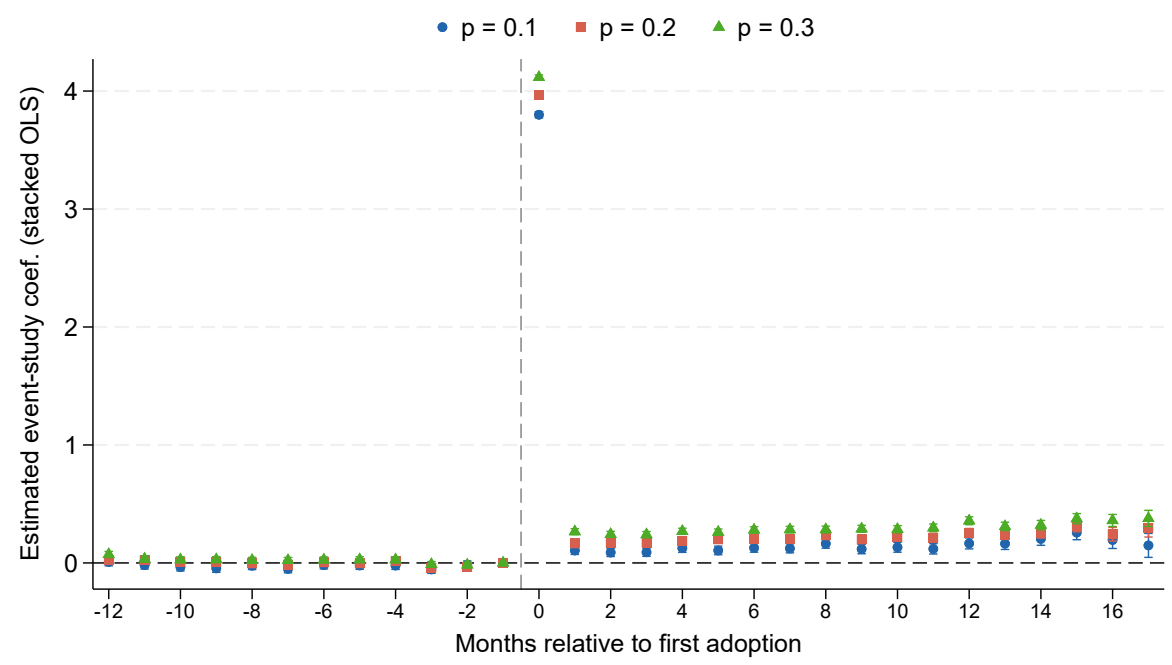

(A) **Placebo 1: random treatment**

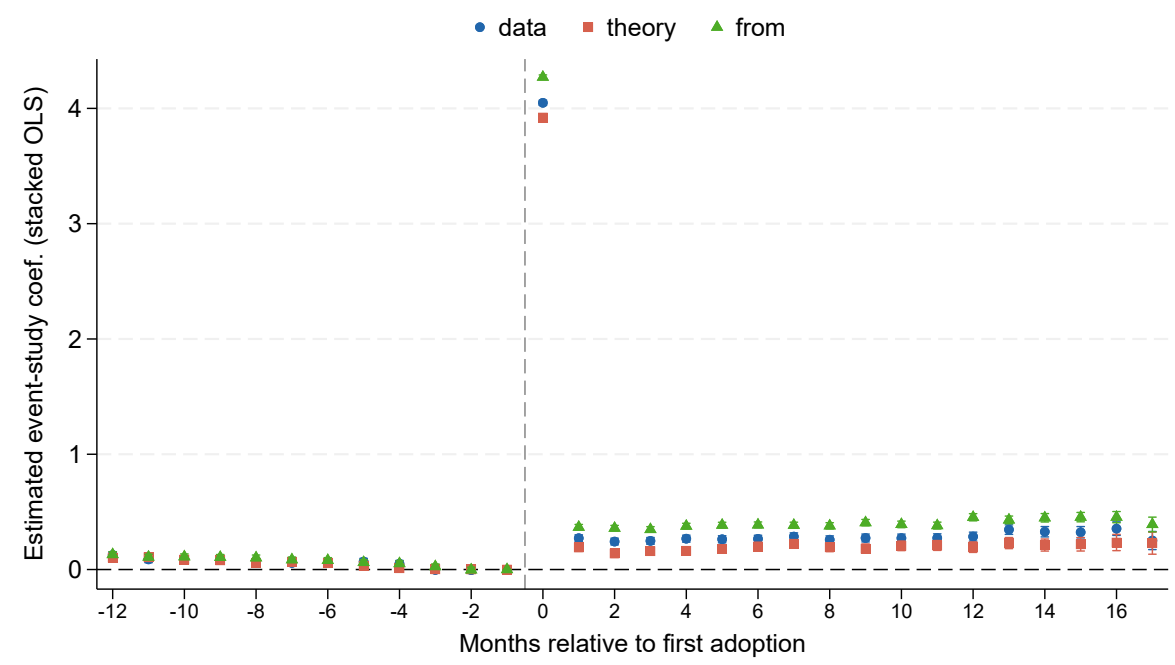

(B) **Placebo 2: keyword treatment**

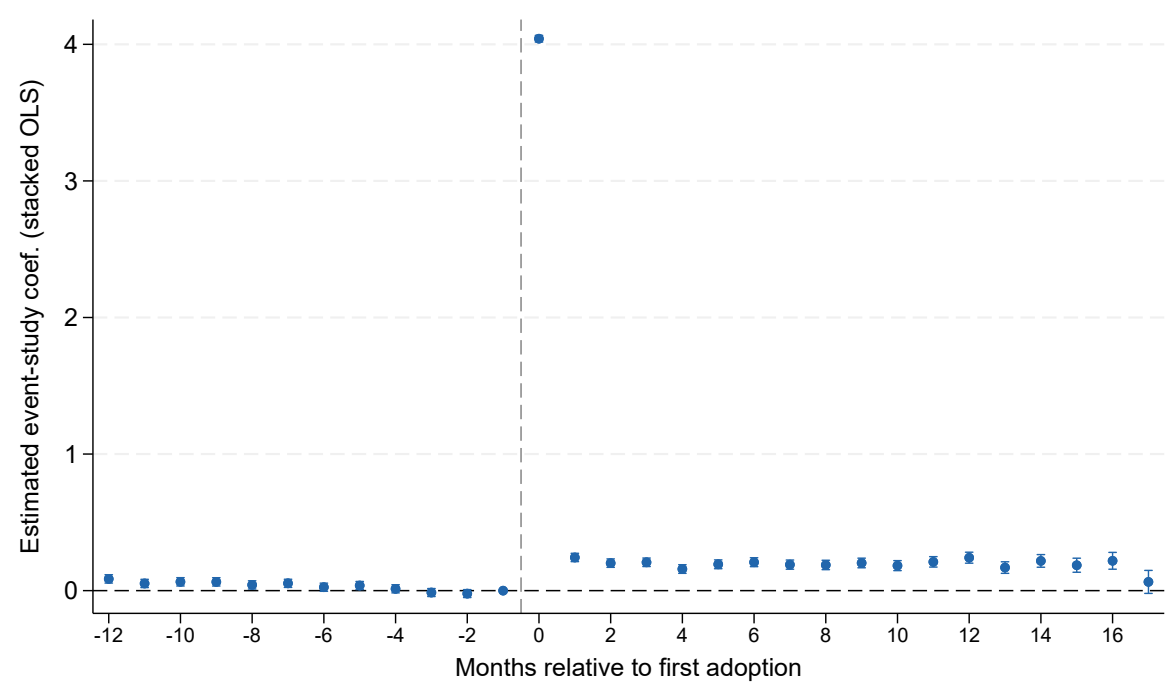

(C) **Placebo 3: pre-ChatGPT period**

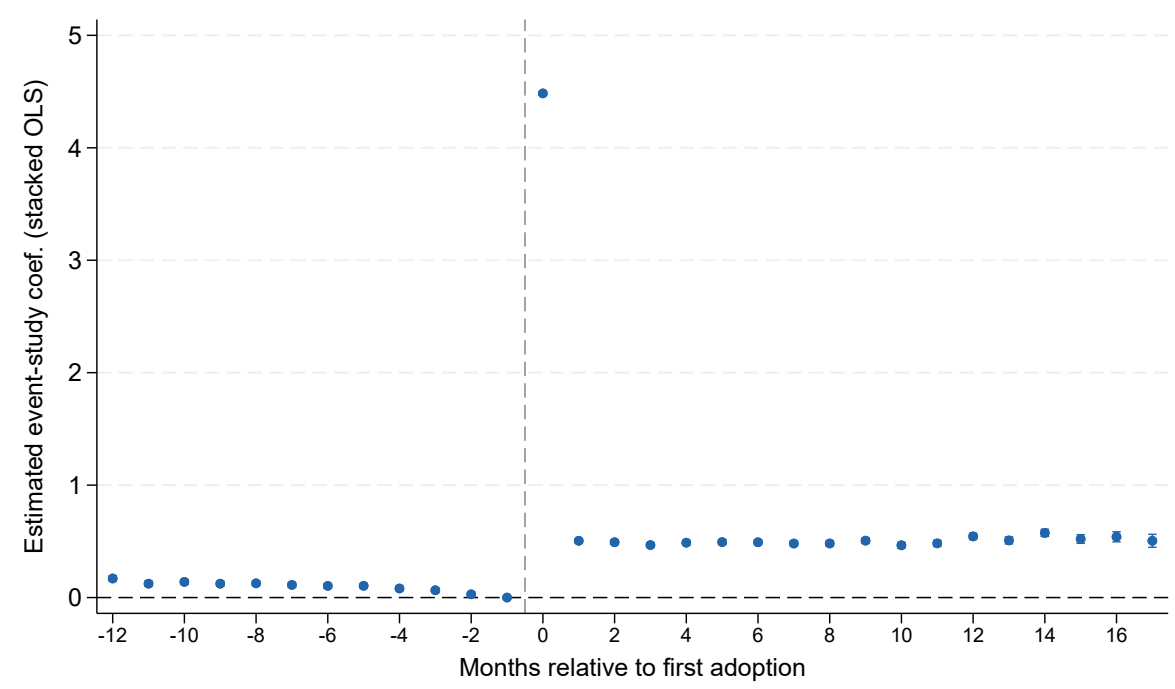

(D) **Placebo 4: inverted treatment**

Figure S23: **Placebo event studies.** All panels report stacked-OLS event-study coefficients, with 95% confidence intervals, of placebo exercises that replace the LLM detector with random paper-level flags (A), neutral keyword flags (B), an earlier observation window (C), and inverted treatment using $\alpha < 0.1$ instead of $\alpha > 0.1$ (D).

10.2.4 with $\ln(x + \sqrt{x^2+1})$ (inverse hyperbolic sine) as dependent variable

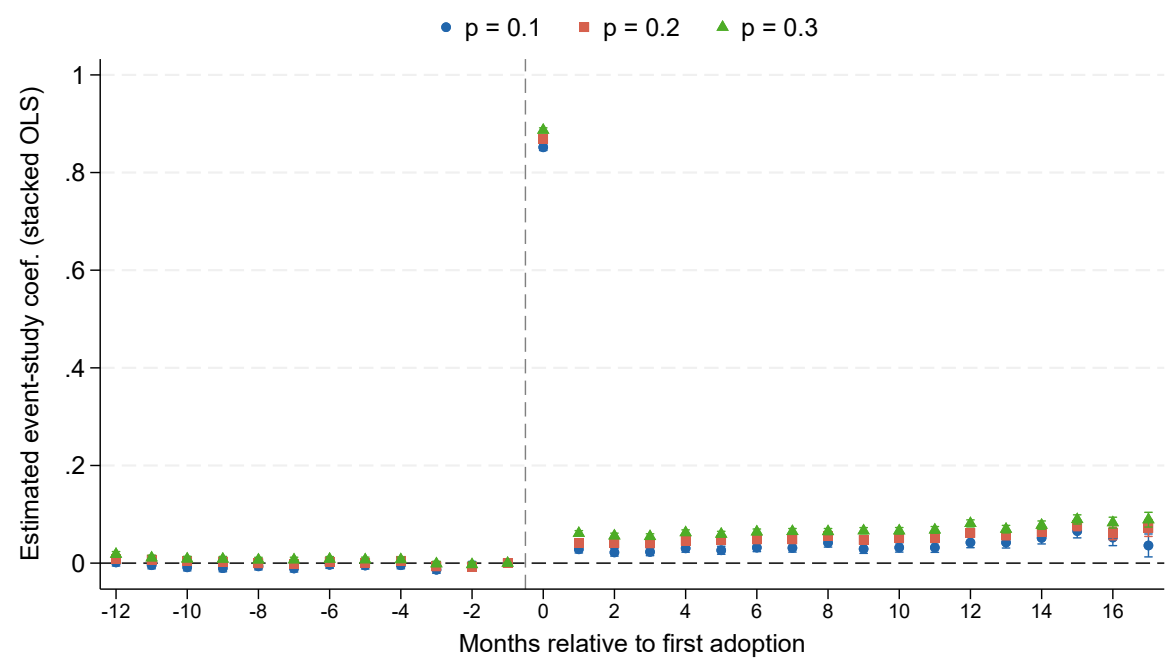

(A) **Placebo 1: random treatment**

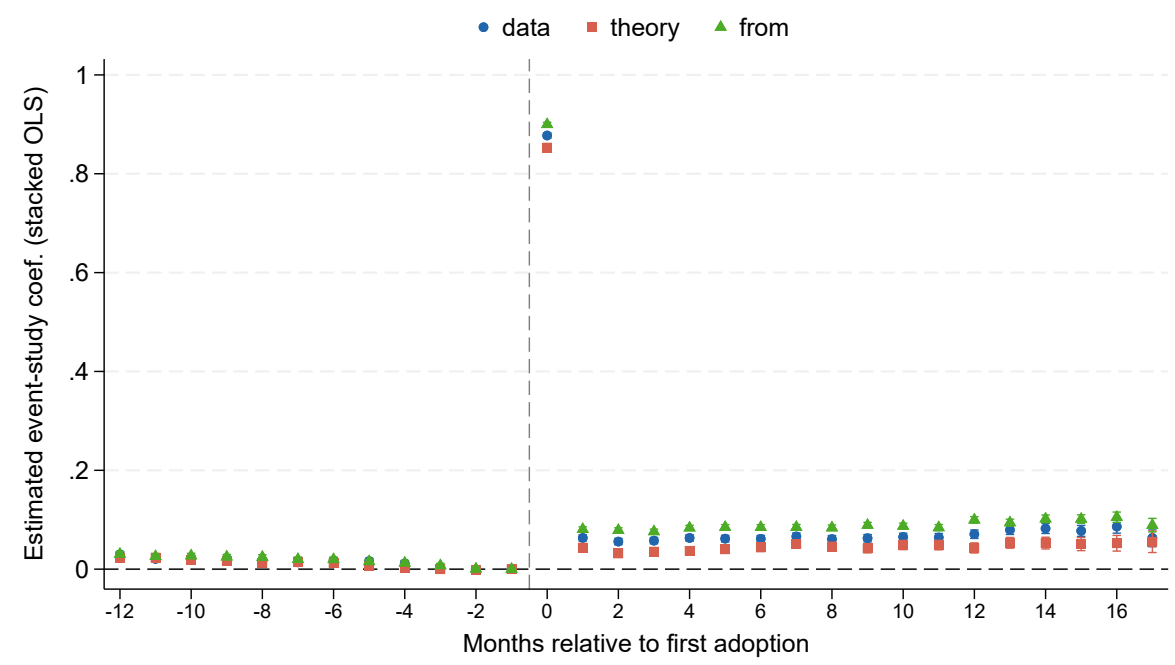

(B) **Placebo 2: keyword treatment**

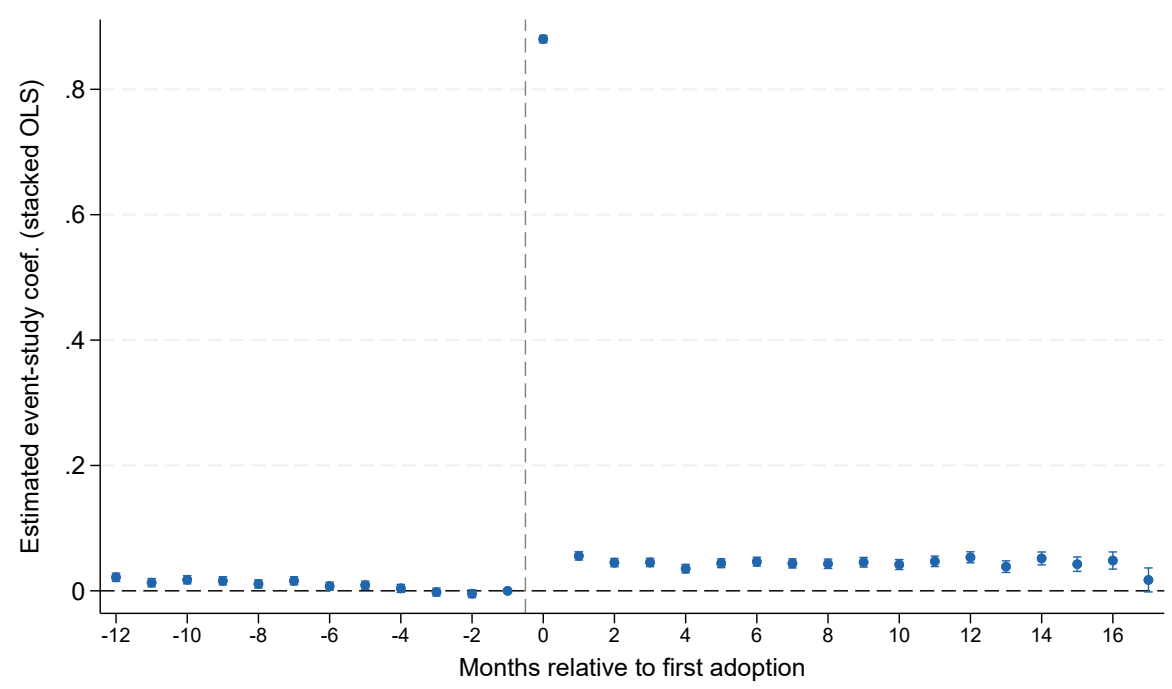

(C) **Placebo 3: pre-ChatGPT period**

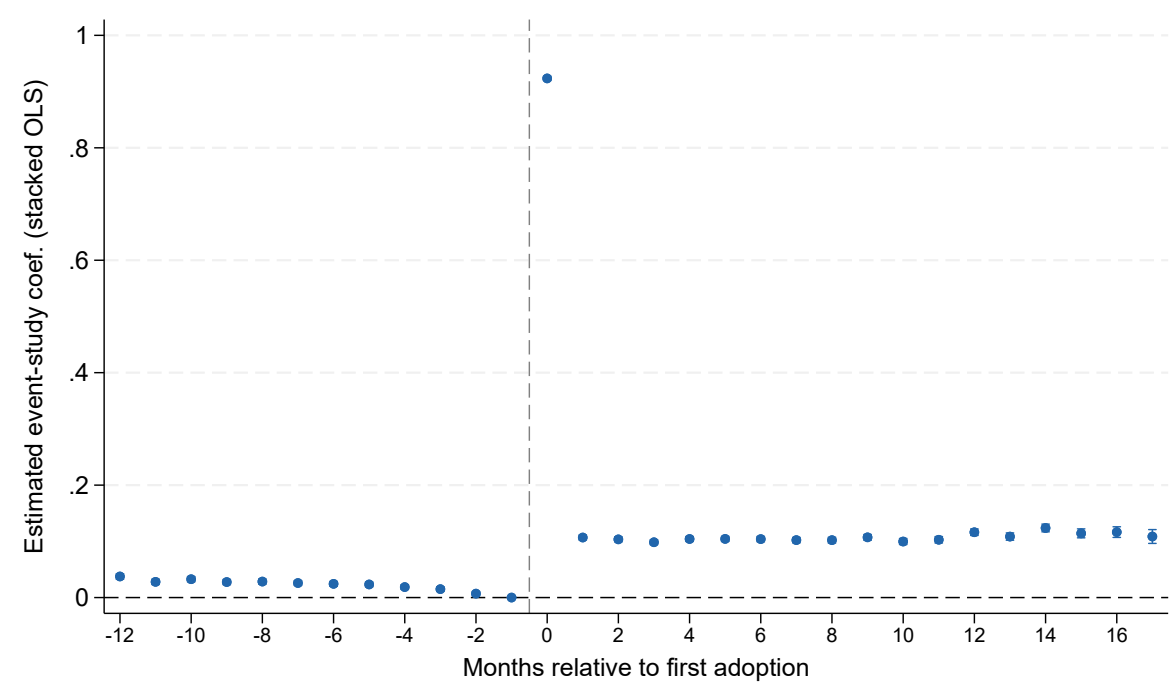

(D) **Placebo 4: inverted treatment**

Figure S24: **Placebo event studies.** All panels report stacked-OLS event-study coefficients, with 95% confidence intervals, of placebo exercises that replace the LLM detector with random paper-level flags (A), neutral keyword flags (B), an earlier observation window (C), and inverted treatment using $\alpha < 0.1$ instead of $\alpha > 0.1$ (D).

10.3 Placebo exercises of Figure 1 with modern event-study estimators for staggered designs

This section replicates the placebo exercises of Figure 1 using modern event-study estimators designed for staggered treatment adoption.

10.3.1 de Chaisemartin and d'Haultfœuille's (2026) estimator

10.3.1.1 with the dependent variable in levels (y = number of papers)

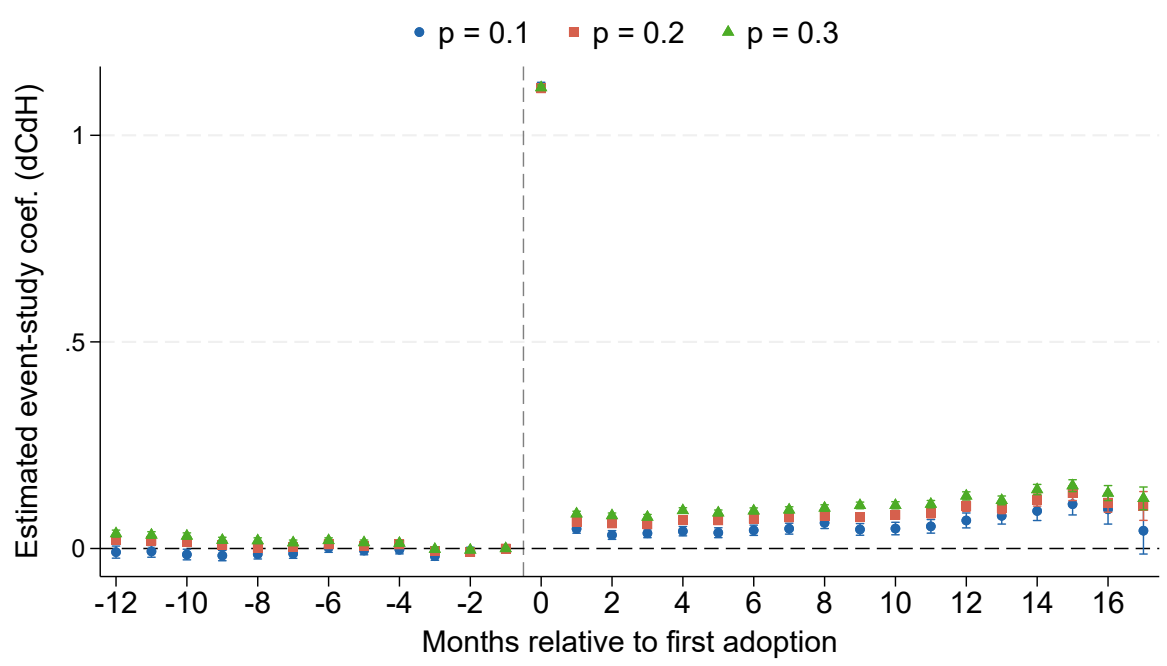

(A) **Placebo 1: random treatment**

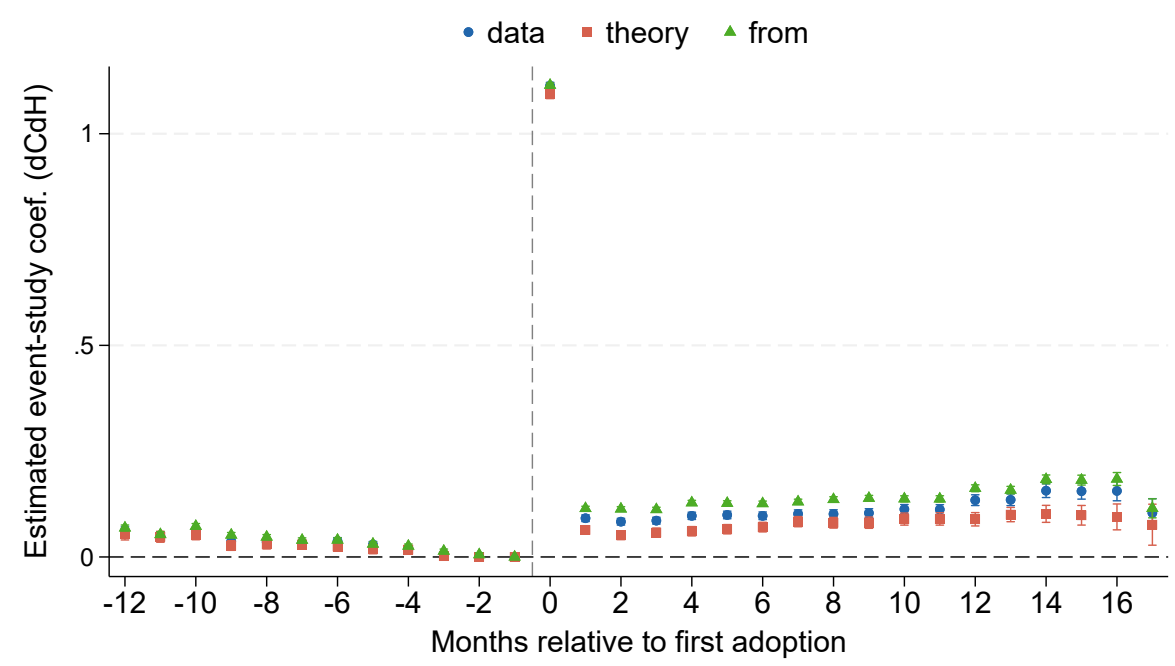

(B) **Placebo 2: keyword treatment**

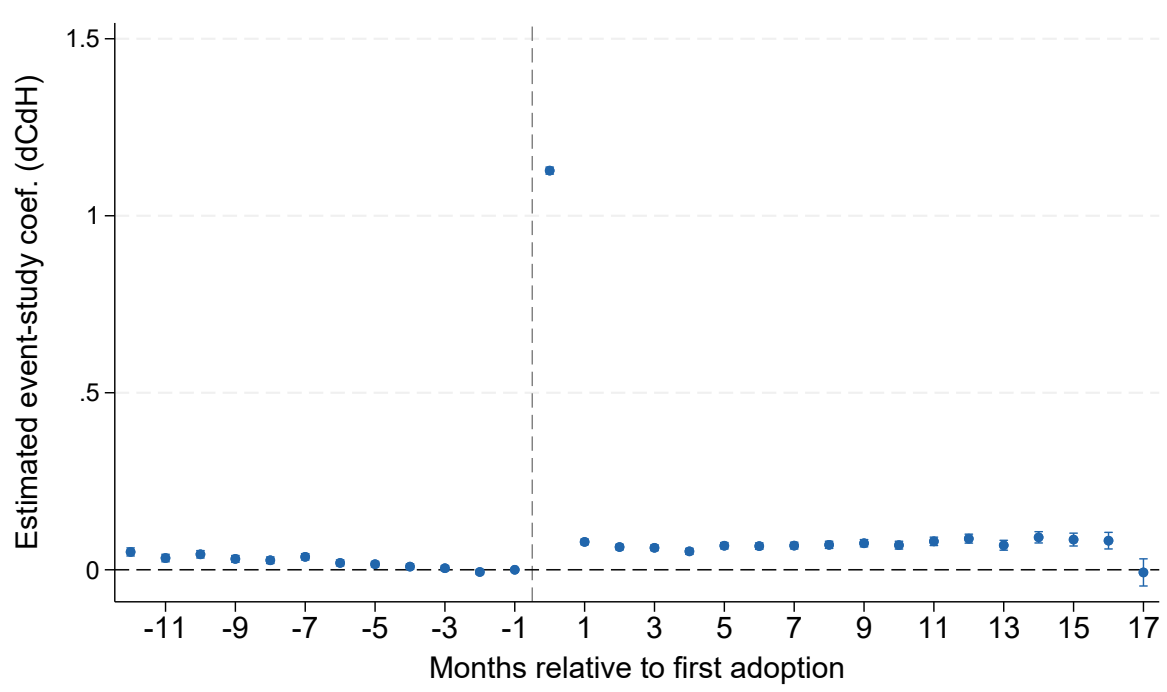

(C) **Placebo 3: pre-ChatGPT period**

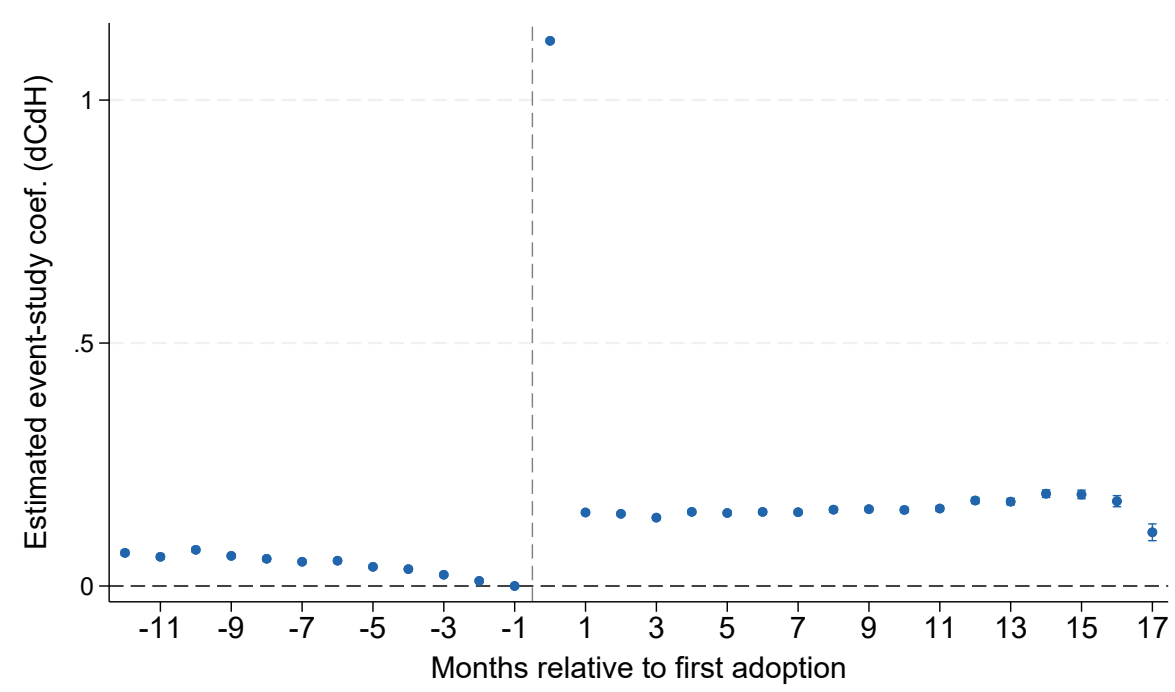

(D) **Placebo 4: inverted treatment**

Figure S25: **Placebo event studies.** All panels report de Chaisemartin and d'Haultfœuille's (2026) event-study coefficients, with 95% confidence intervals, of placebo exercises that replace the LLM detector with random paper-level flags (A), neutral keyword flags (B), an earlier observation window (C), and inverted treatment using $\alpha < 0.1$ instead of $\alpha > 0.1$ (D).

10.3.1.2 with $\ln(0.1 + y)$ as dependent variable

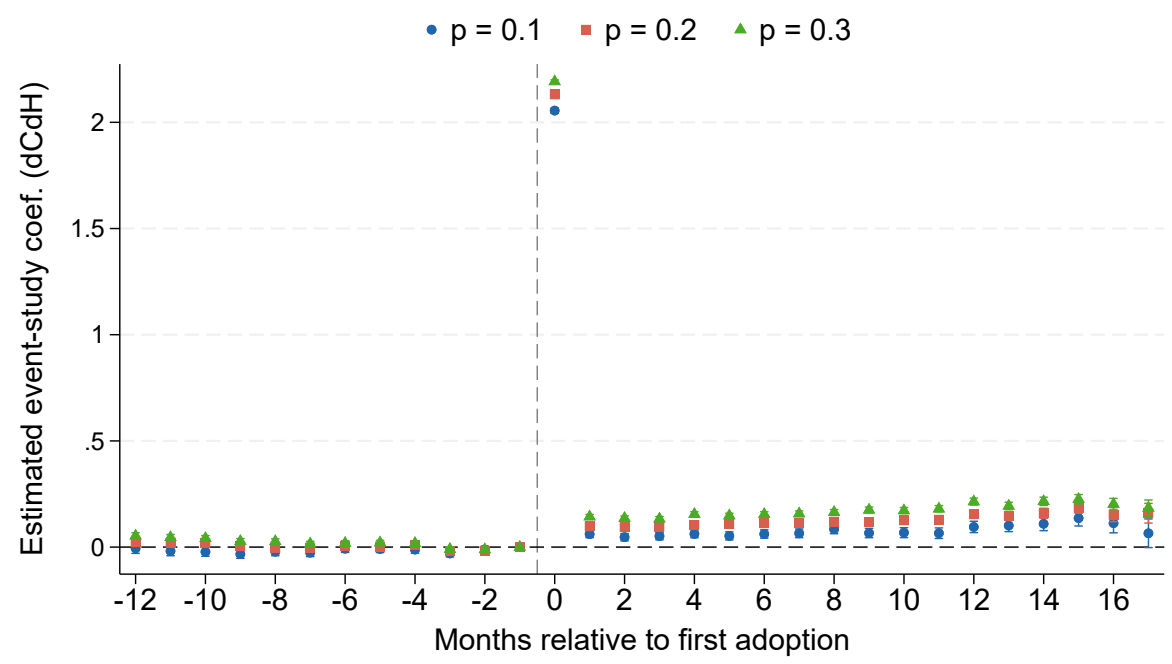

(A) **Placebo 1: random treatment**

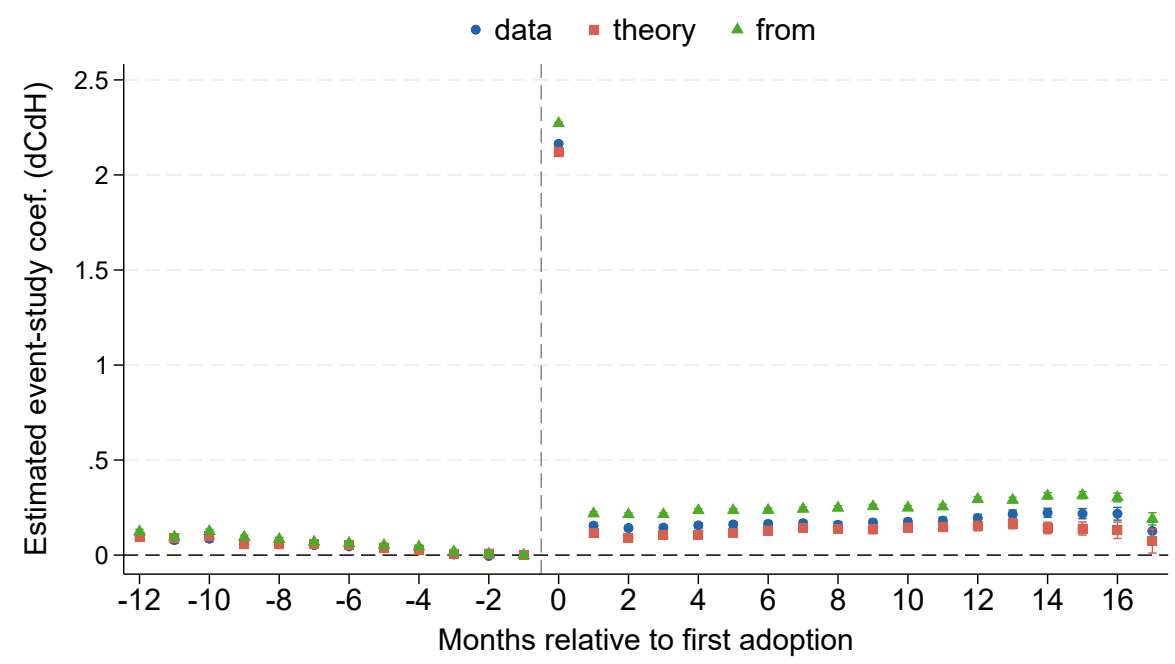

(B) **Placebo 2: keyword treatment**

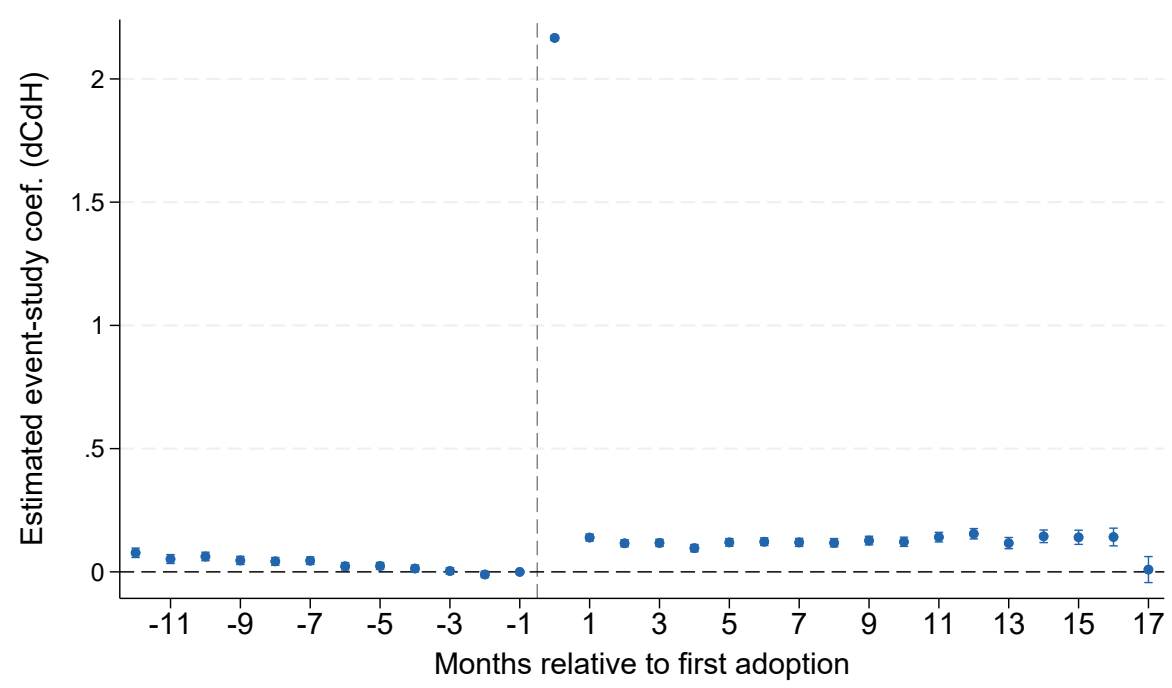

(C) **Placebo 3: pre-ChatGPT period**

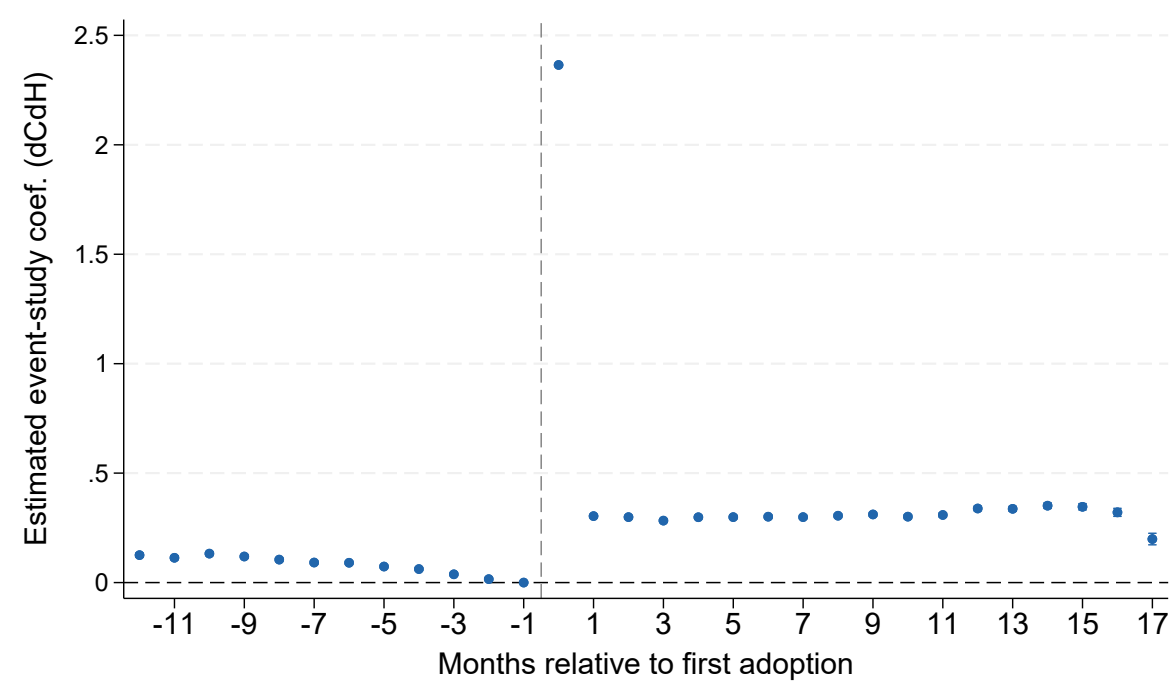

(D) **Placebo 4: inverted treatment**

Figure S26: **Placebo event studies.** All panels report de Chaisemartin and d'Haultfœuille's (2026) event-study coefficients, with 95% confidence intervals, of placebo exercises that replace the LLM detector with random paper-level flags (A), neutral keyword flags (B), an earlier observation window (C), and inverted treatment using $\alpha < 0.1$ instead of $\alpha > 0.1$ (D).

10.3.1.3 with $\ln(0.01 + y)$ as dependent variable

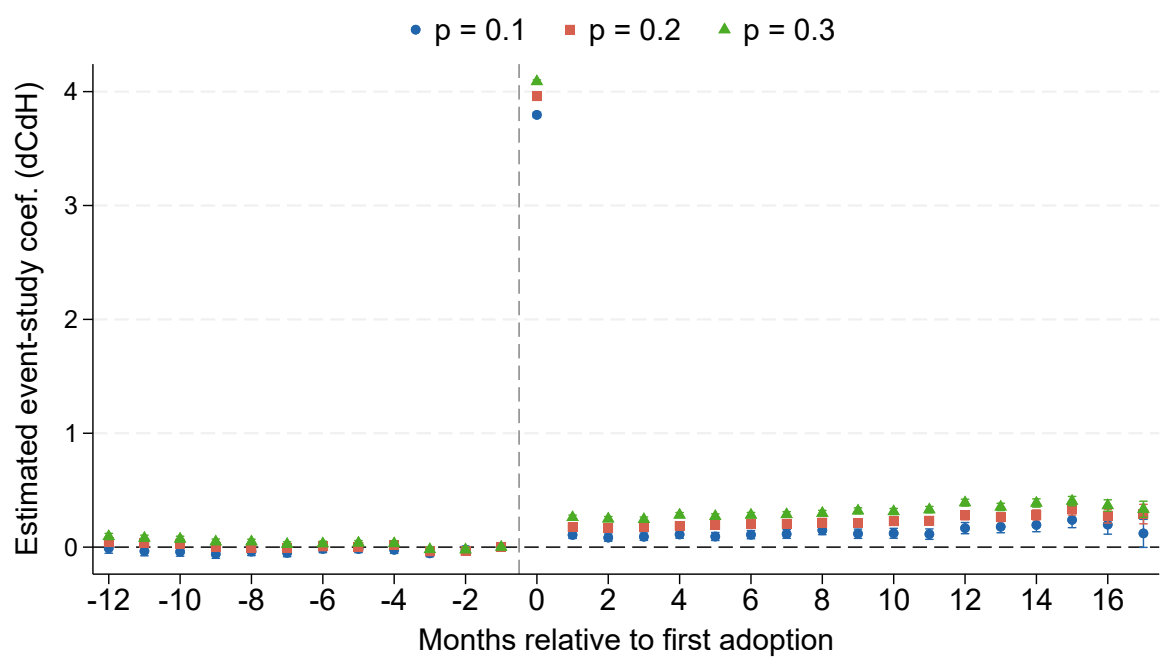

(A) **Placebo 1: random treatment**

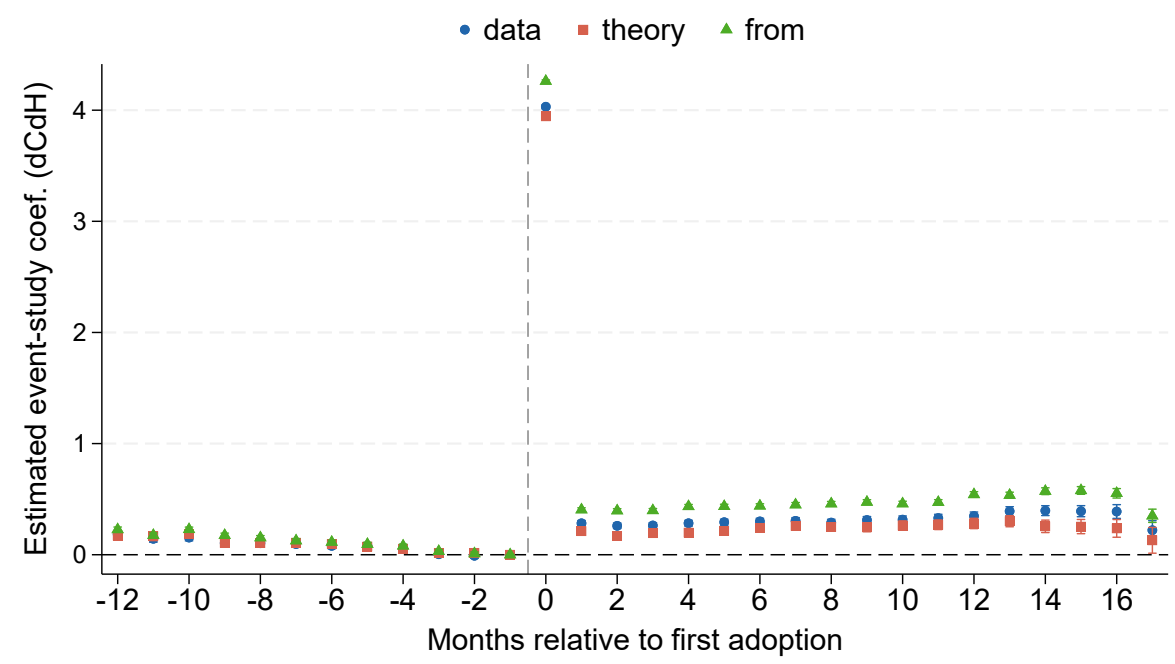

(B) **Placebo 2: keyword treatment**

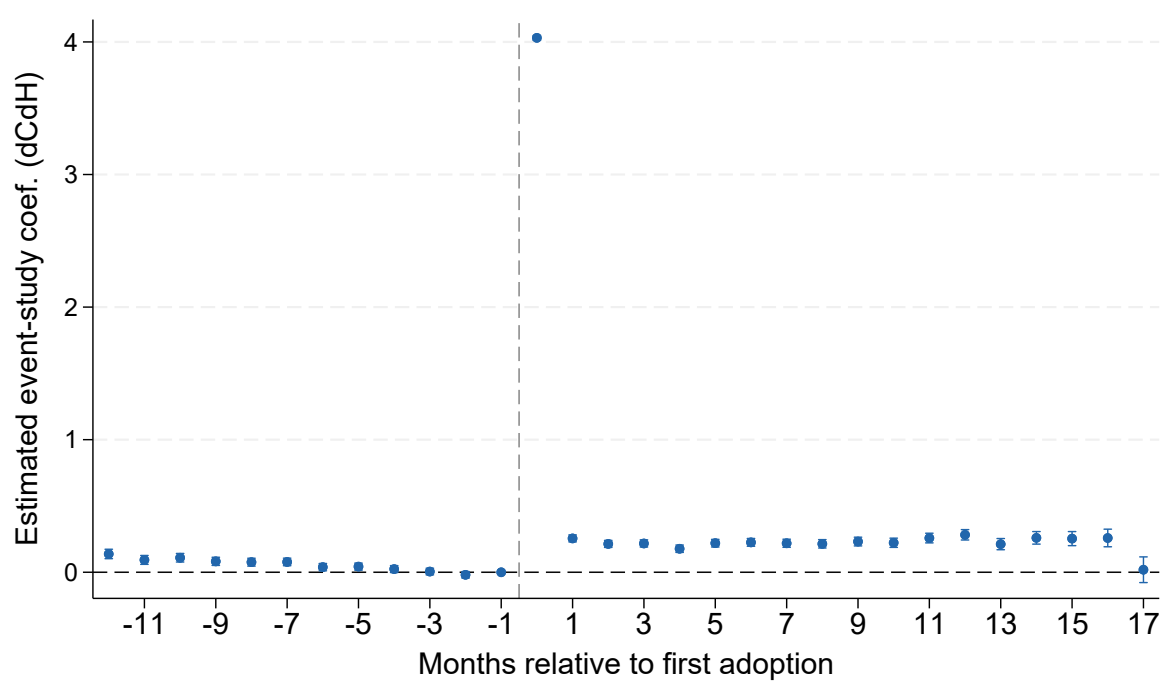

(C) **Placebo 3: pre-ChatGPT period**

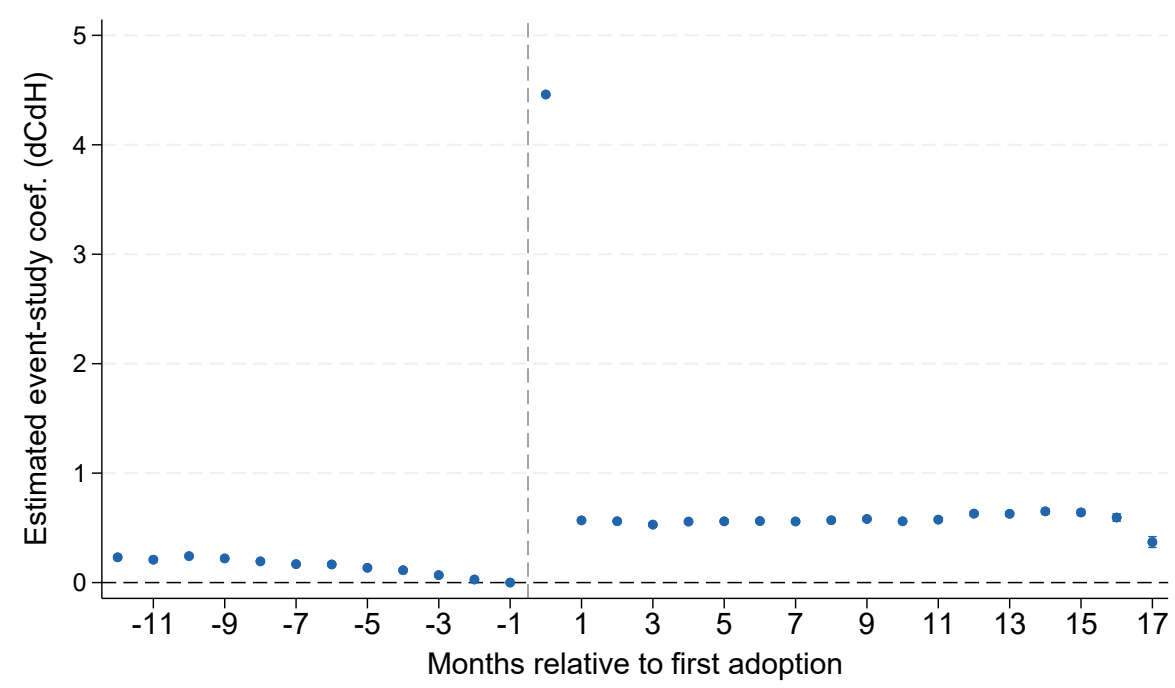

(D) **Placebo 4: inverted treatment**

Figure S27: **Placebo event studies.** All panels report de Chaisemartin and d'Haultfœuille's (2026) event-study coefficients, with 95% confidence intervals, of placebo exercises that replace the LLM detector with random paper-level flags (A), neutral keyword flags (B), an earlier observation window (C), and inverted treatment using $\alpha < 0.1$ instead of $\alpha > 0.1$ (D).

10.3.1.4 with $\ln(x + \sqrt{x^2 + 1})$ (inverse hyperbolic sine) as dependent variable

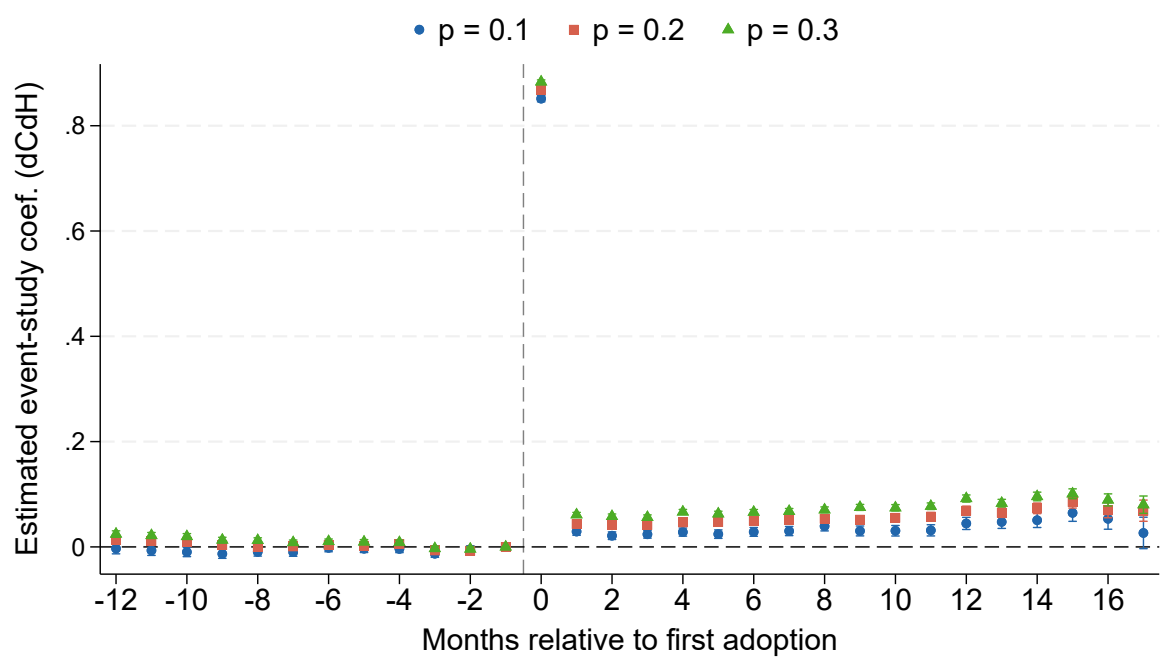

(A) **Placebo 1: random treatment**

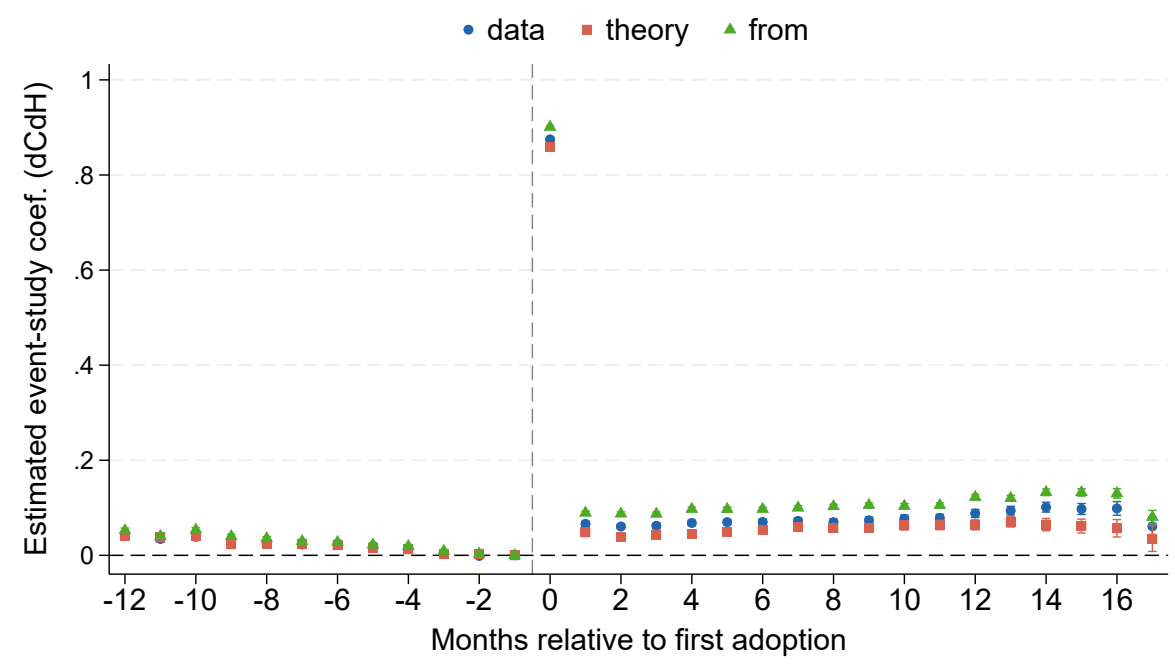

(B) **Placebo 2: keyword treatment**

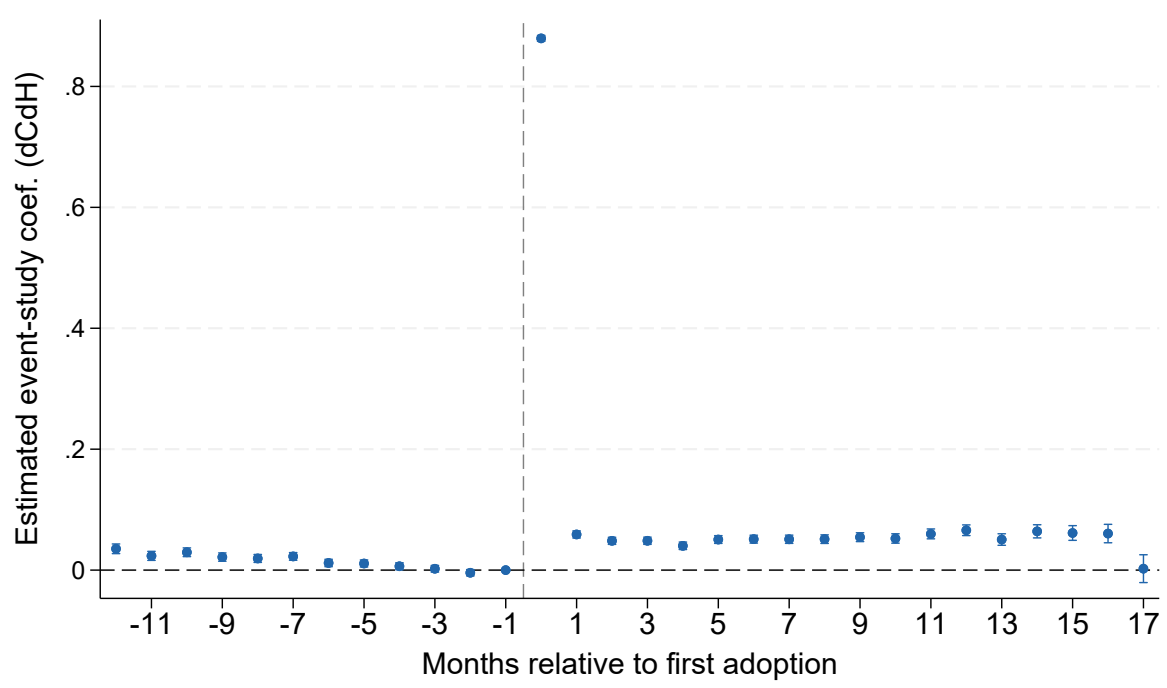

(C) **Placebo 3: pre-ChatGPT period**

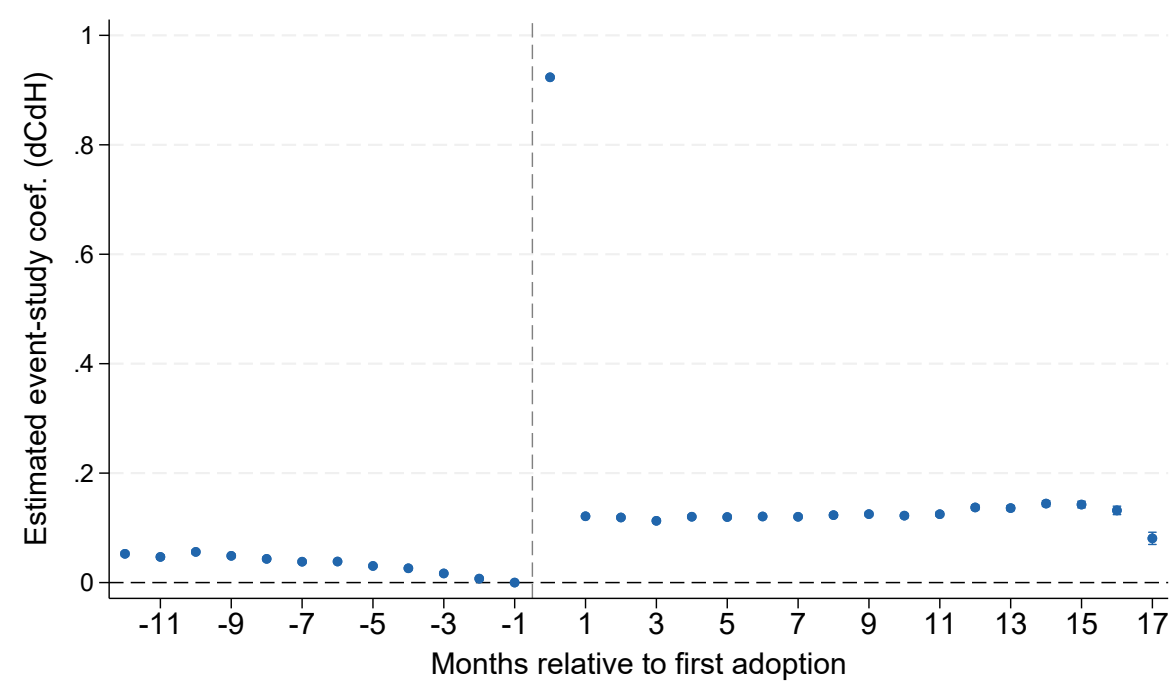

(D) **Placebo 4: inverted treatment**

Figure S28: **Placebo event studies.** All panels report de Chaisemartin and d'Haultfœuille's (2026) event-study coefficients, with 95% confidence intervals, of placebo exercises that replace the LLM detector with random paper-level flags (A), neutral keyword flags (B), an earlier observation window (C), and inverted treatment using $\alpha < 0.1$ instead of $\alpha > 0.1$ (D).

10.3.2 Sun and Abraham's (2021) estimator

10.3.2.1 with the dependent variable in levels (y = number of papers)

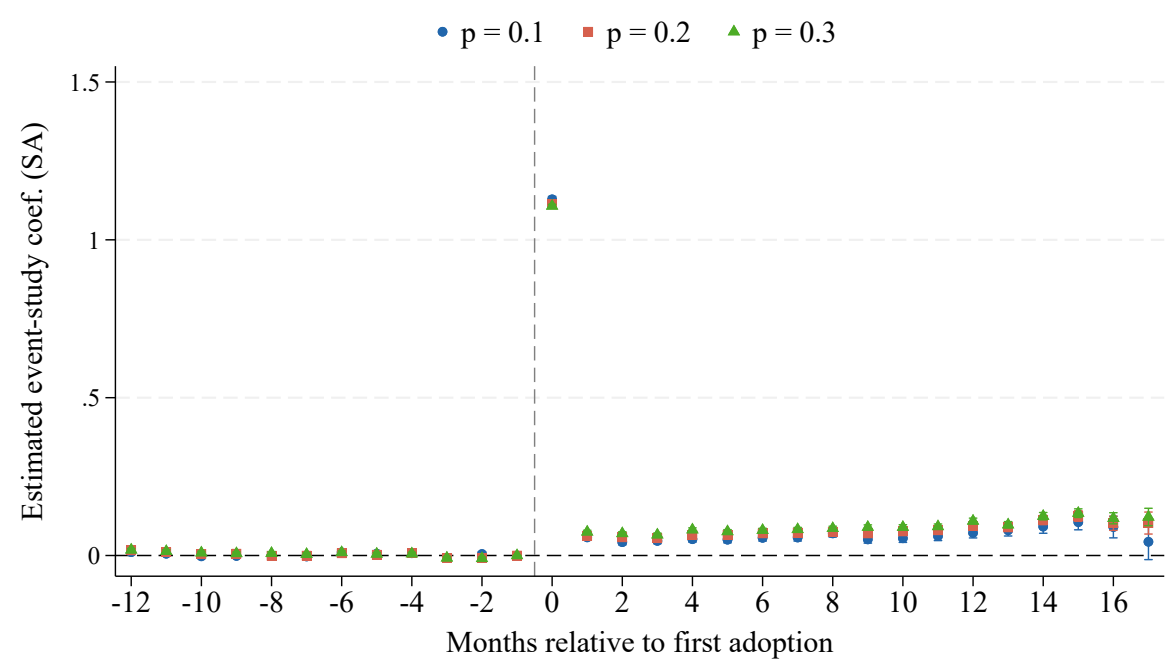

(A) **Placebo 1: random treatment**

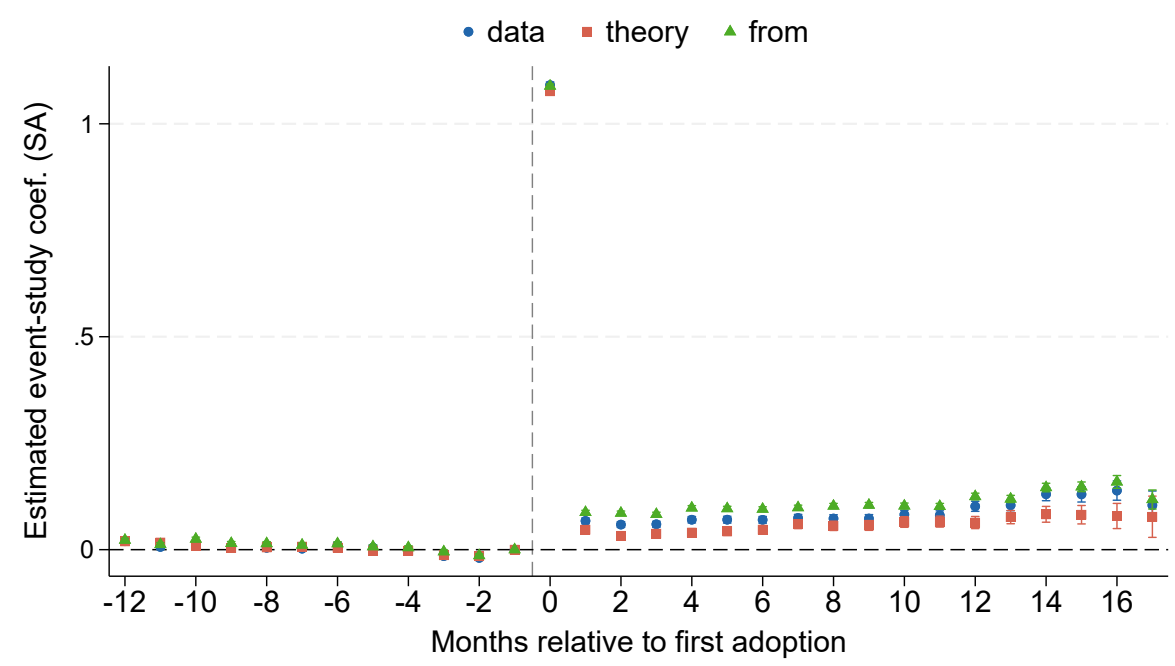

(B) **Placebo 2: keyword treatment**

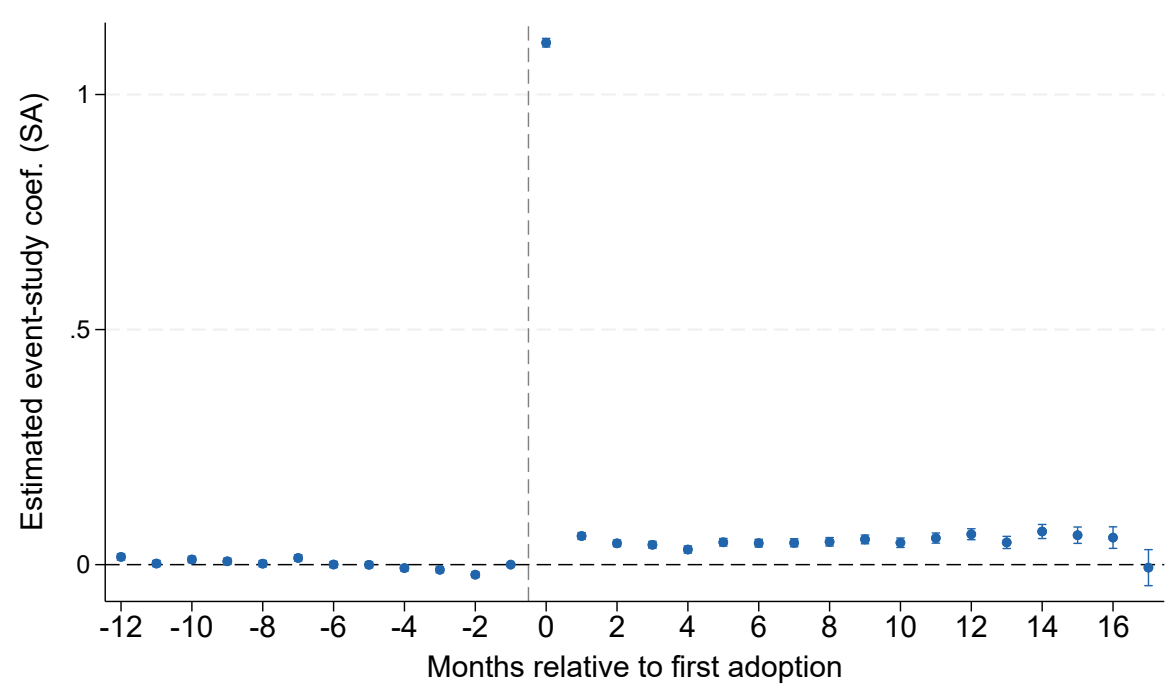

(C) **Placebo 3: pre-ChatGPT period**

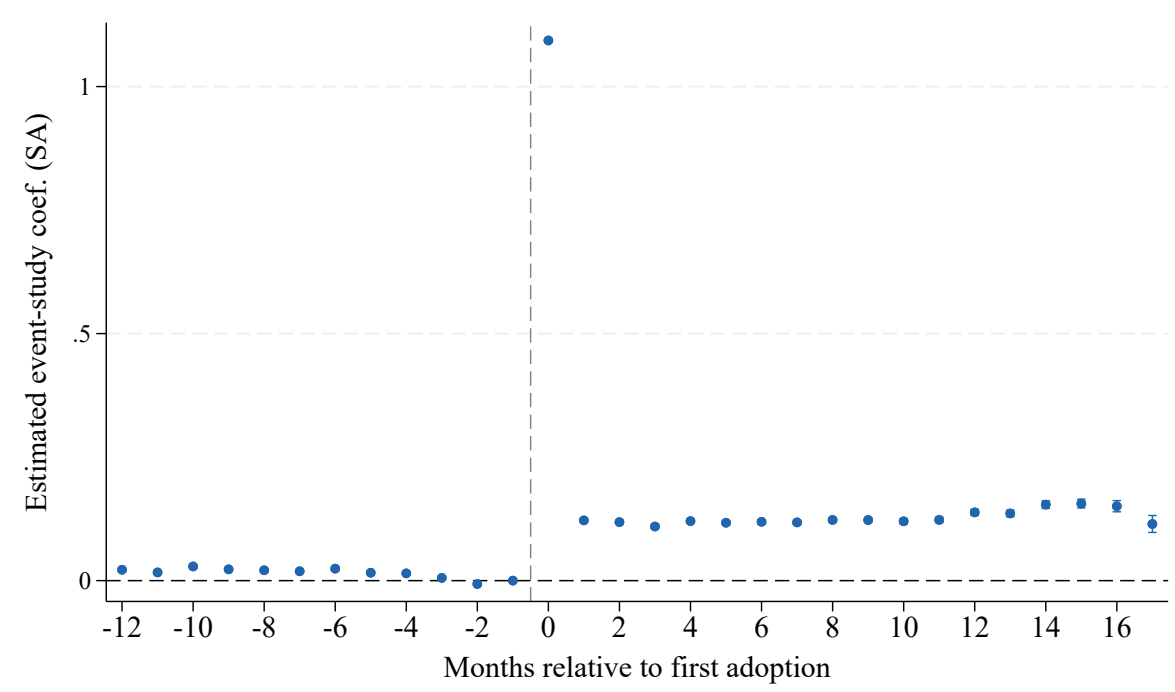

(D) **Placebo 4: inverted treatment**

Figure S29: **Placebo event studies.** All panels report Sun and Abraham's (2021) event-study coefficients, with 95% confidence intervals, of placebo exercises that replace the LLM detector with random paper-level flags (A), neutral keyword flags (B), an earlier observation window (C), and inverted treatment using $\alpha < 0.1$ instead of $\alpha > 0.1$ (D).

10.3.2.2 with $\ln(0.1 + y)$ as dependent variable

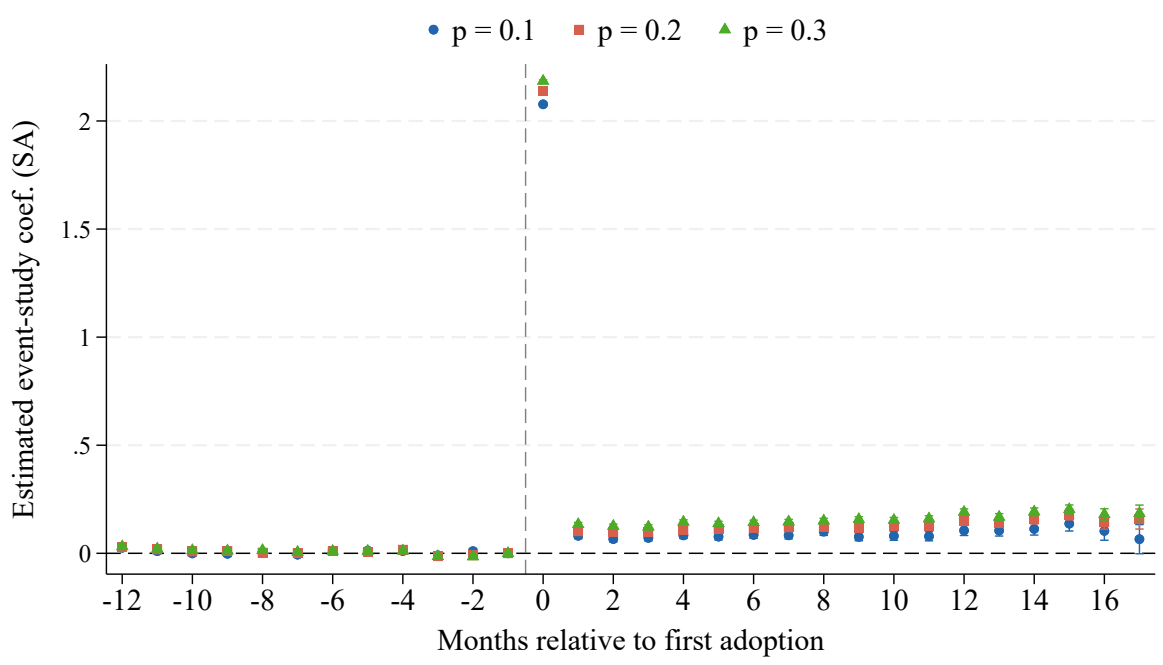

(A) **Placebo 1: random treatment**

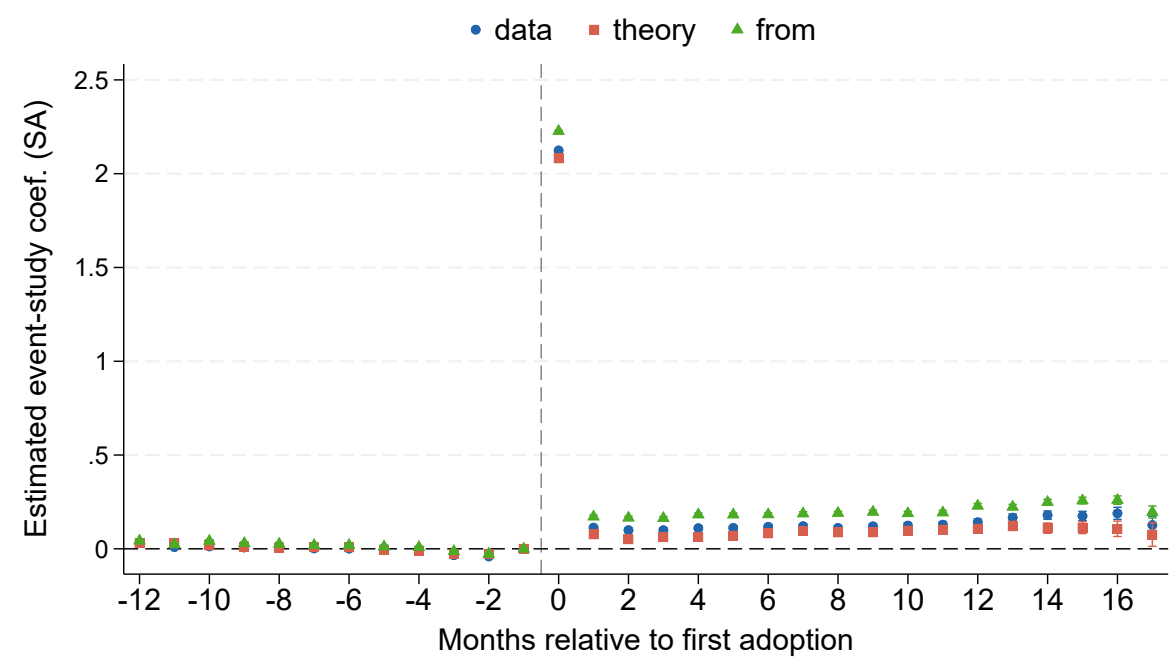

(B) **Placebo 2: keyword treatment**

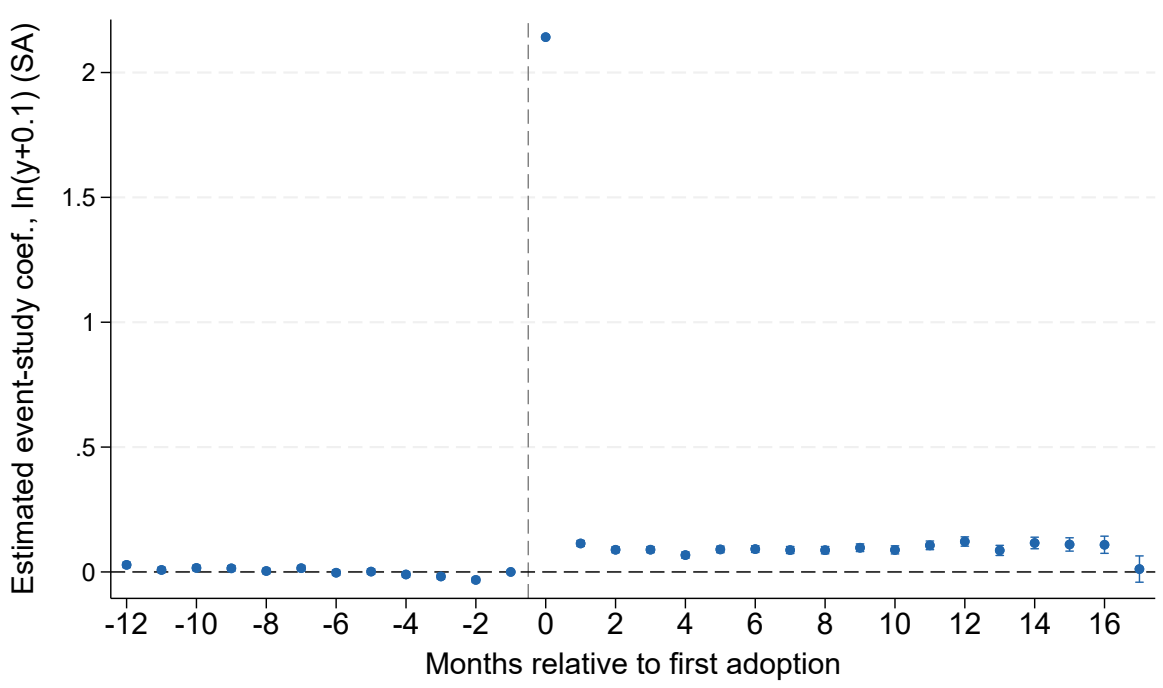

(C) **Placebo 3: pre-ChatGPT period**

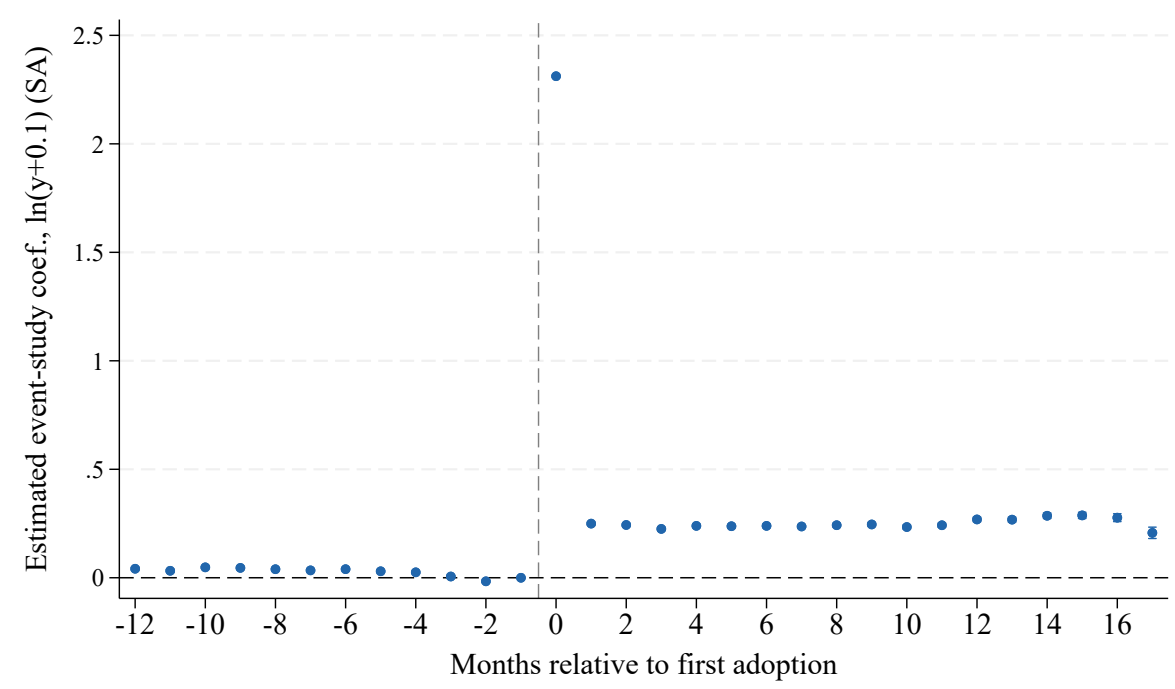

(D) **Placebo 4: inverted treatment**

Figure S30: **Placebo event studies.** All panels report Sun and Abraham's (2021) event-study coefficients, with 95% confidence intervals, of placebo exercises that replace the LLM detector with random paper-level flags (A), neutral keyword flags (B), an earlier observation window (C), and inverted treatment using $\alpha < 0.1$ instead of $\alpha > 0.1$ (D).

10.3.2.3 with $\ln(0.01 + y)$ as dependent variable

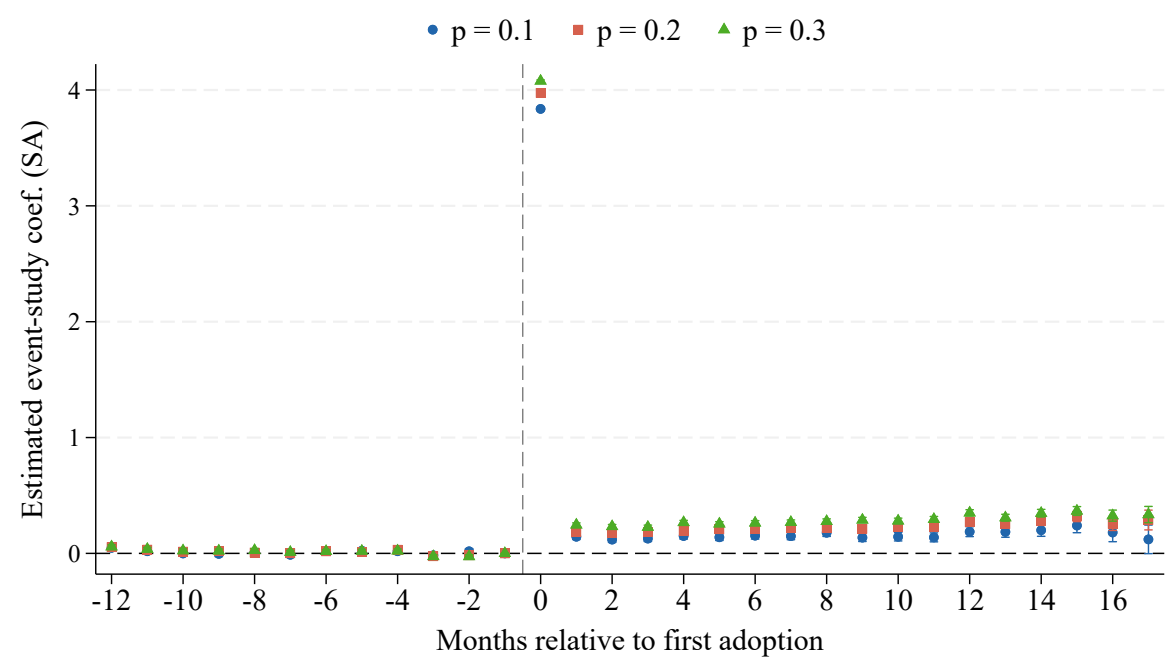

(A) **Placebo 1: random treatment**

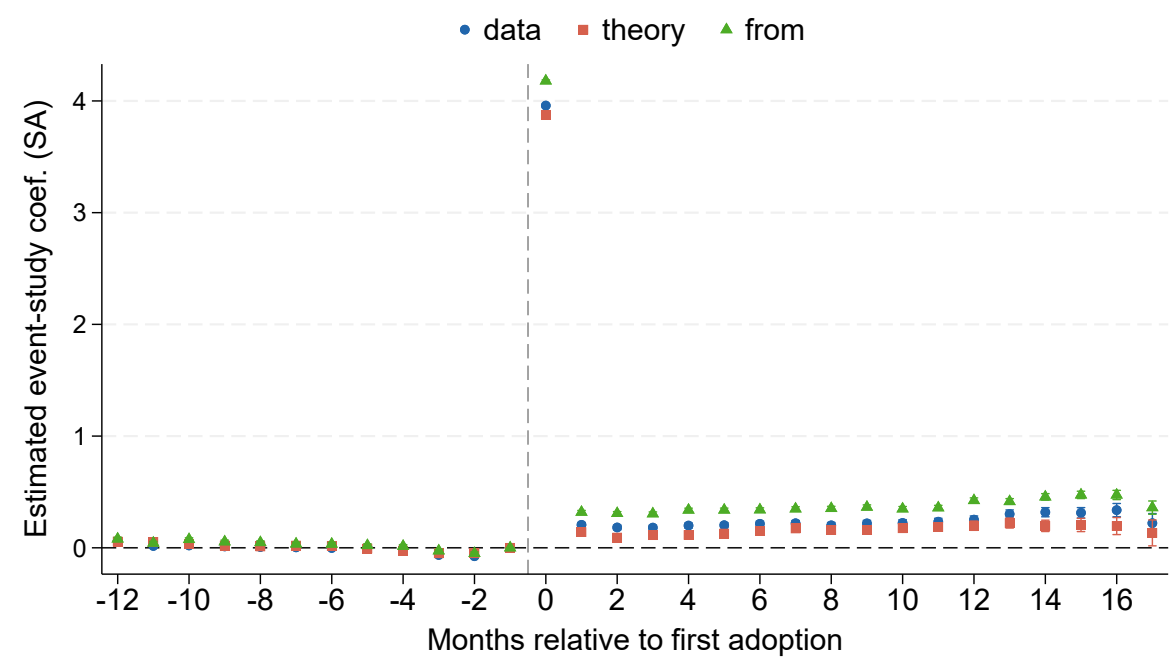

(B) **Placebo 2: keyword treatment**

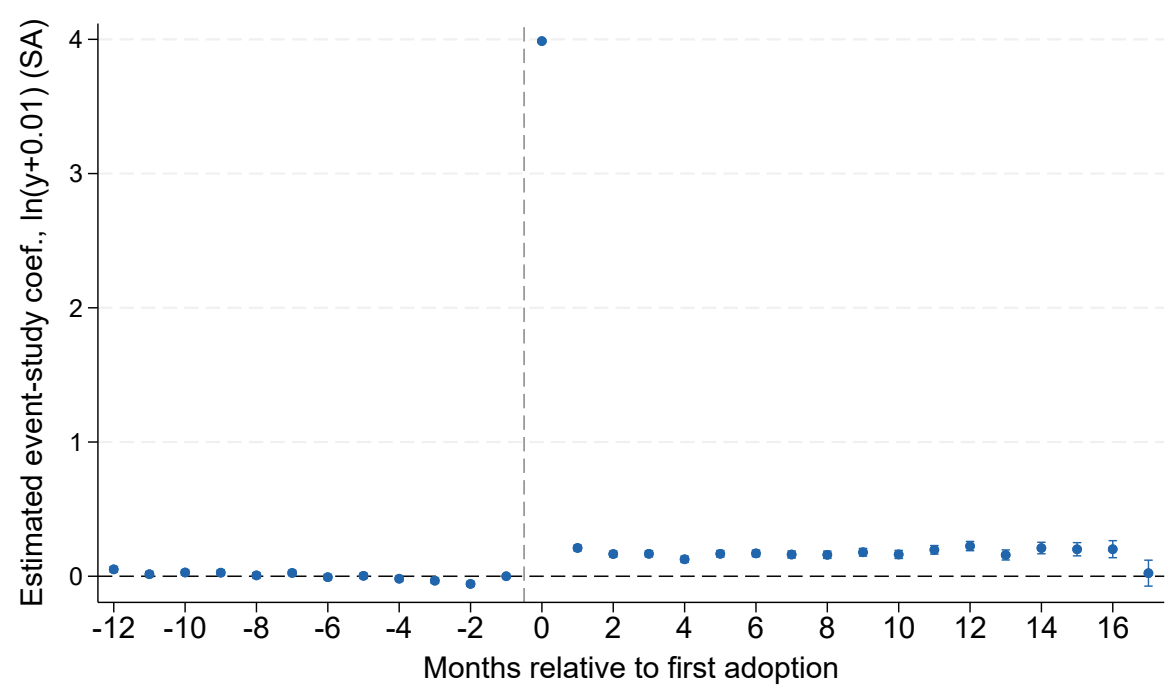

(C) **Placebo 3: pre-ChatGPT period**

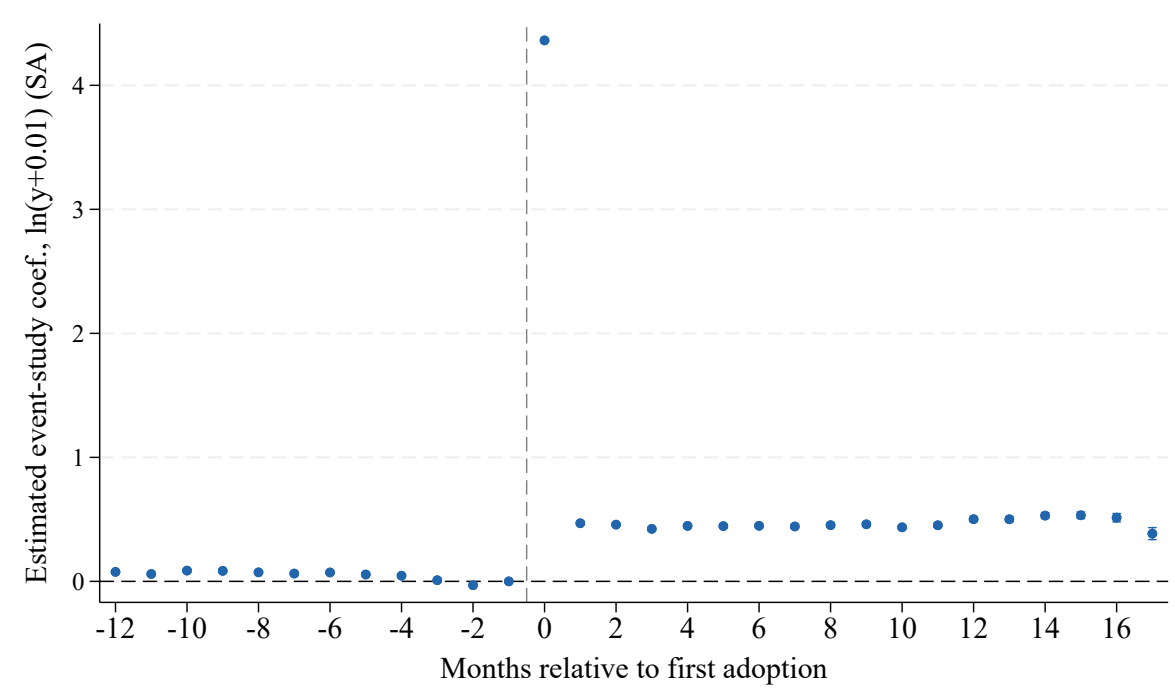

(D) **Placebo 4: inverted treatment**

Figure S31: **Placebo event studies.** All panels report Sun and Abraham's (2021) event-study coefficients, with 95% confidence intervals, of placebo exercises that replace the LLM detector with random paper-level flags (A), neutral keyword flags (B), an earlier observation window (C), and inverted treatment using $\alpha < 0.1$ instead of $\alpha > 0.1$ (D).

10.3.2.4 with $\ln(x + \sqrt{x^2 + 1})$ (inverse hyperbolic sine) as dependent variable

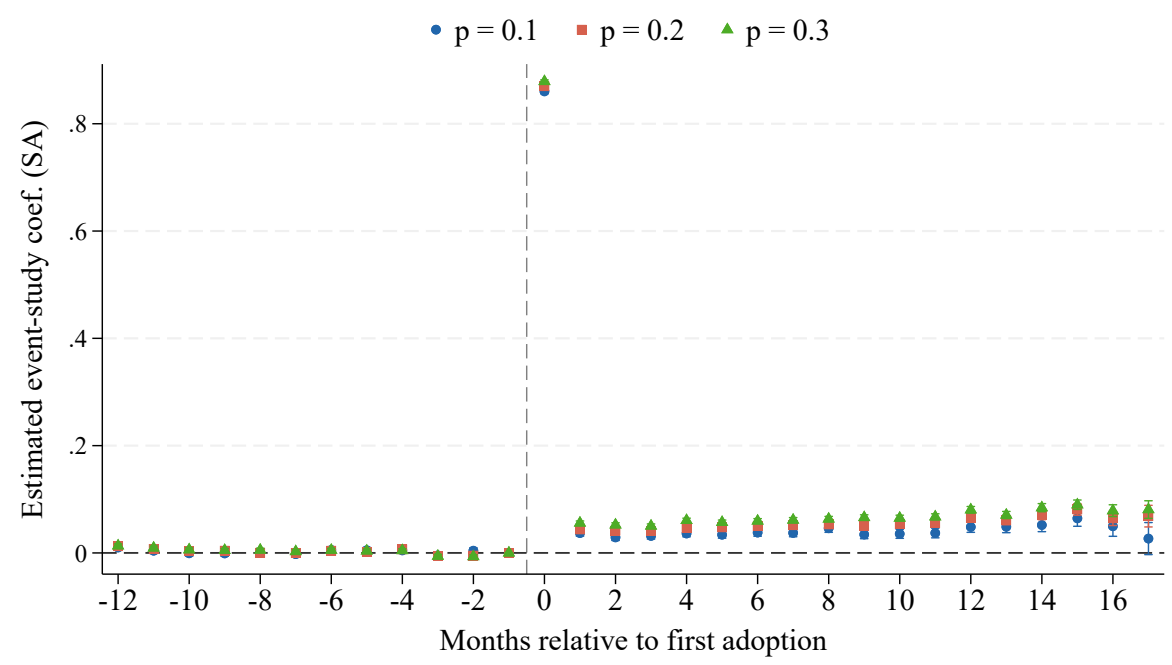

(A) **Placebo 1: random treatment**

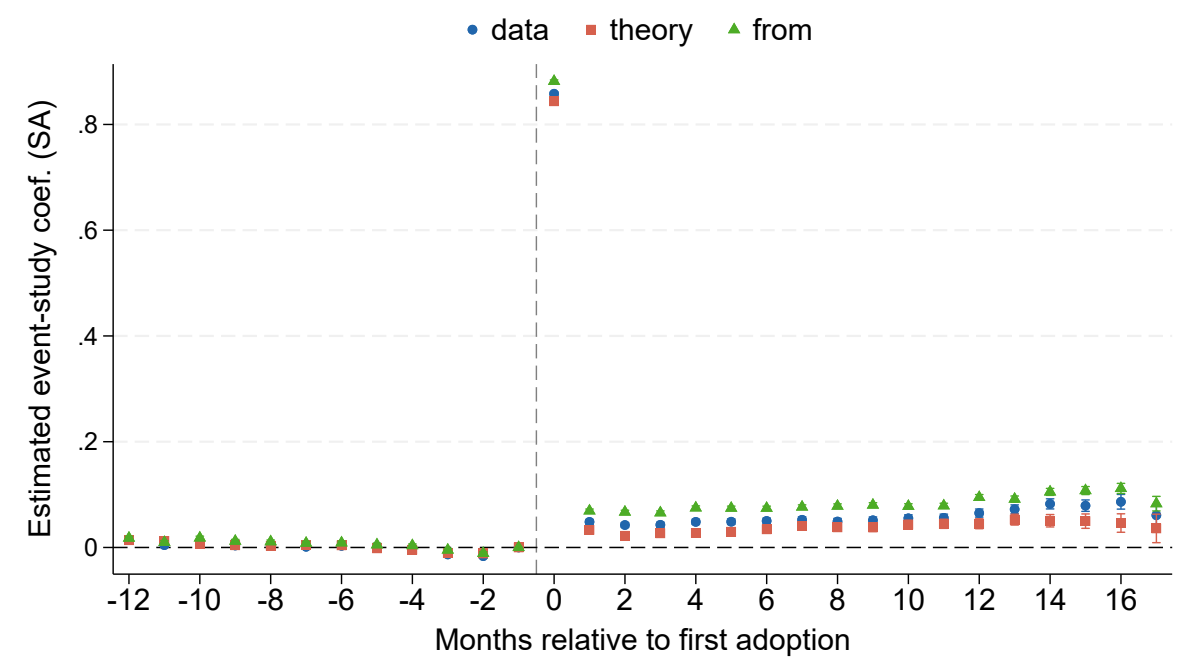

(B) **Placebo 2: keyword treatment**

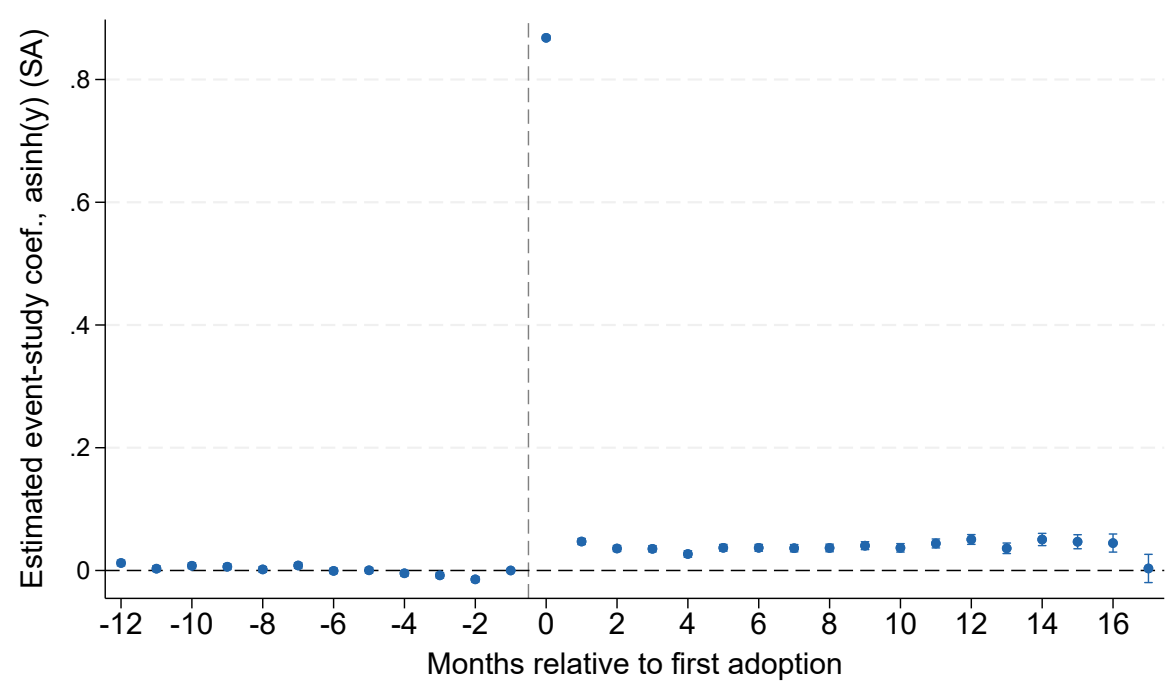

(C) **Placebo 3: pre-ChatGPT period**

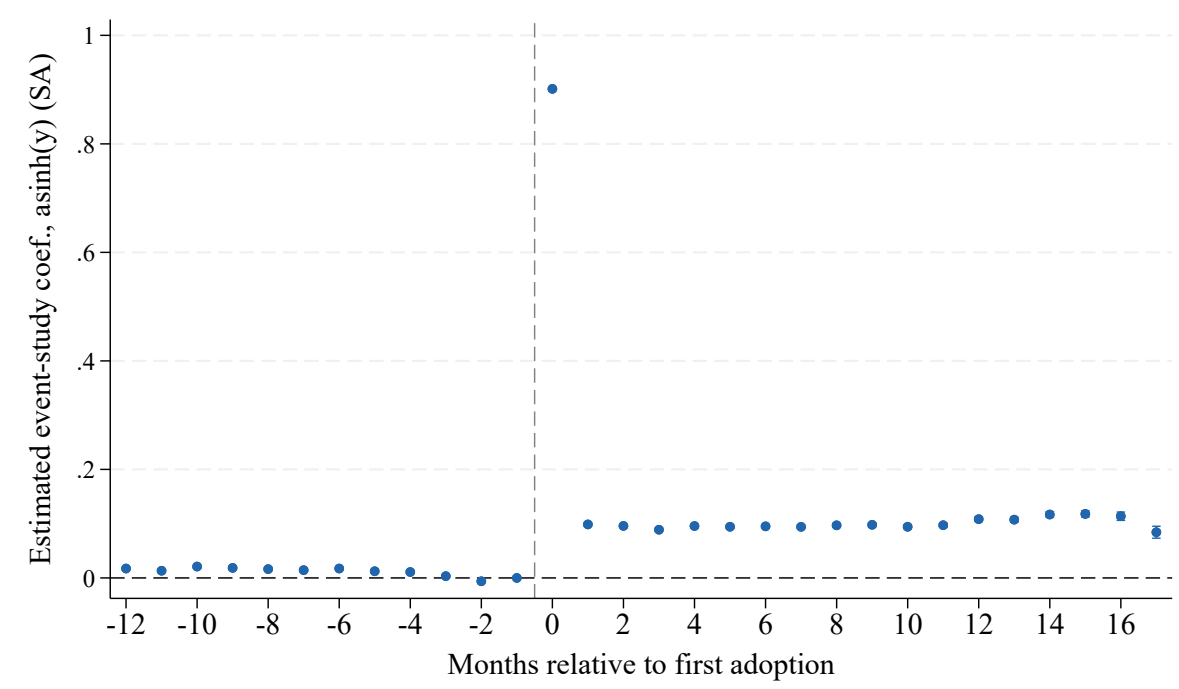

(D) **Placebo 4: inverted treatment**

Figure S32: **Placebo event studies.** All panels report Sun and Abraham's (2021) event-study coefficients, with 95% confidence intervals, of placebo exercises that replace the LLM detector with random paper-level flags (A), neutral keyword flags (B), an earlier observation window (C), and inverted treatment using $\alpha < 0.1$ instead of $\alpha > 0.1$ (D).

10.4 Random treatment placebo with event-time balanced windows

Figure S33 reports the random treatment placebo results using event-time balanced windows, ensuring that the comparison between treated and control authors covers the same range of relative periods.

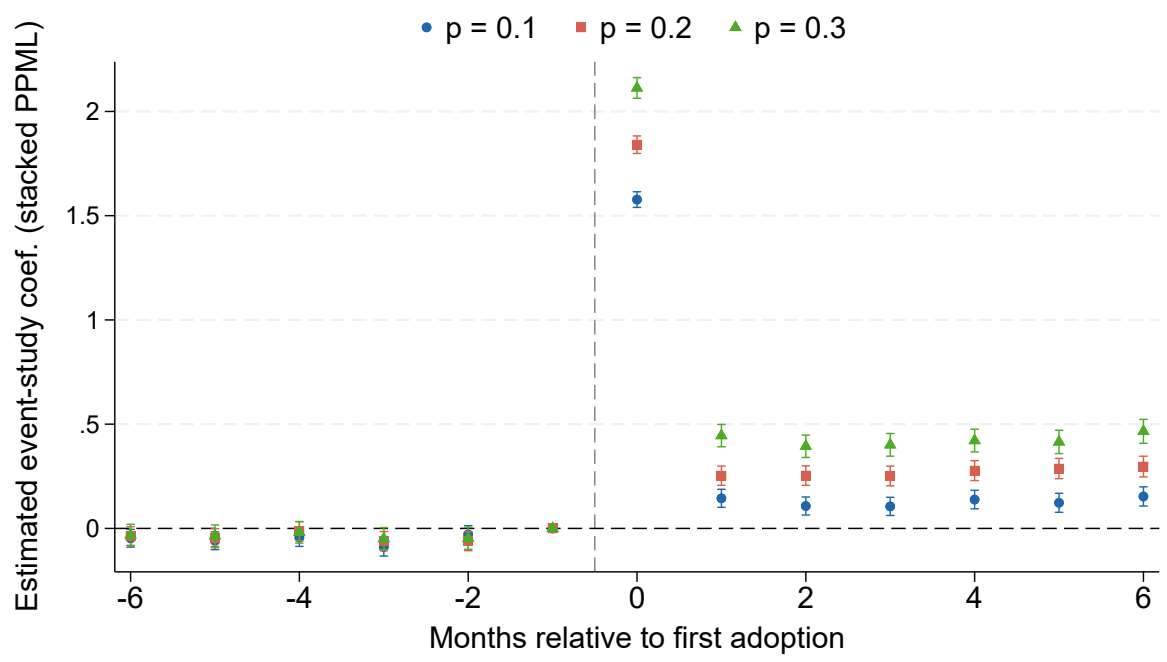

(A) **Balanded window:** $[-6; +6]$

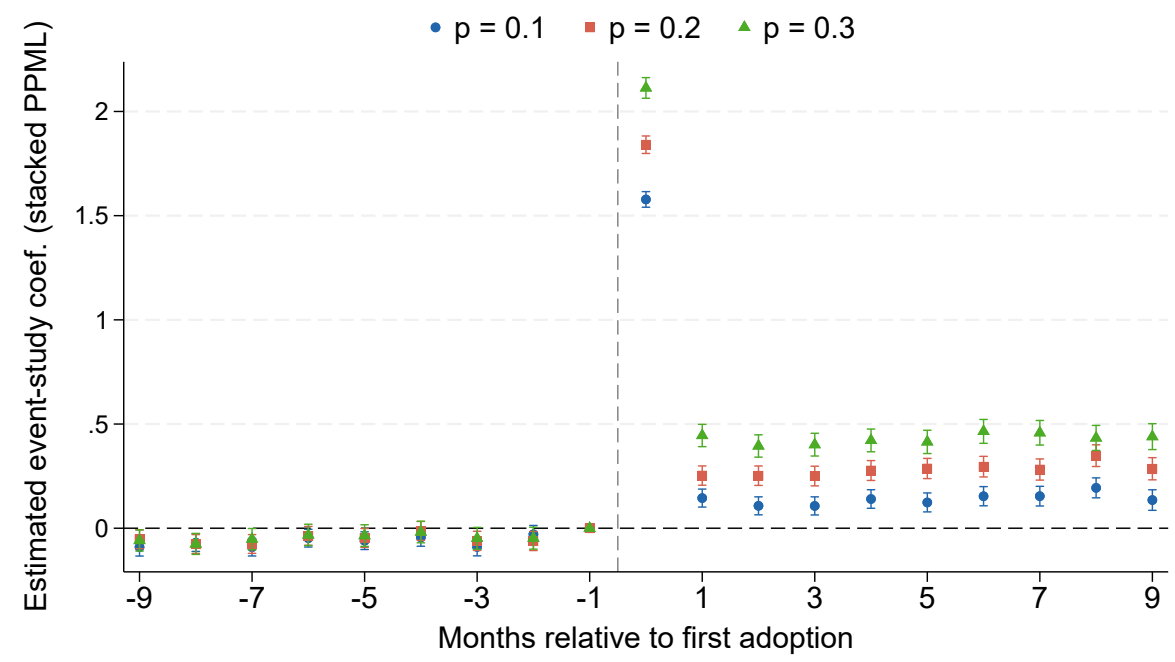

(B) **Balanded window:** $[-9; +9]$

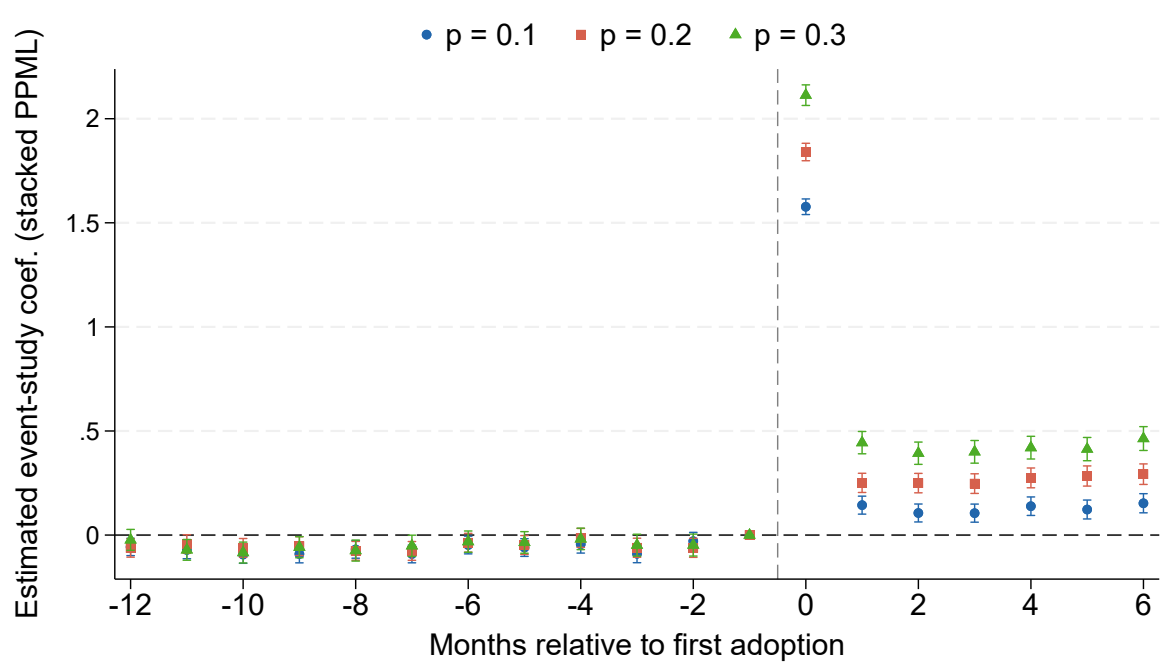

(C) **Balanded window:** $[-12; +6]$

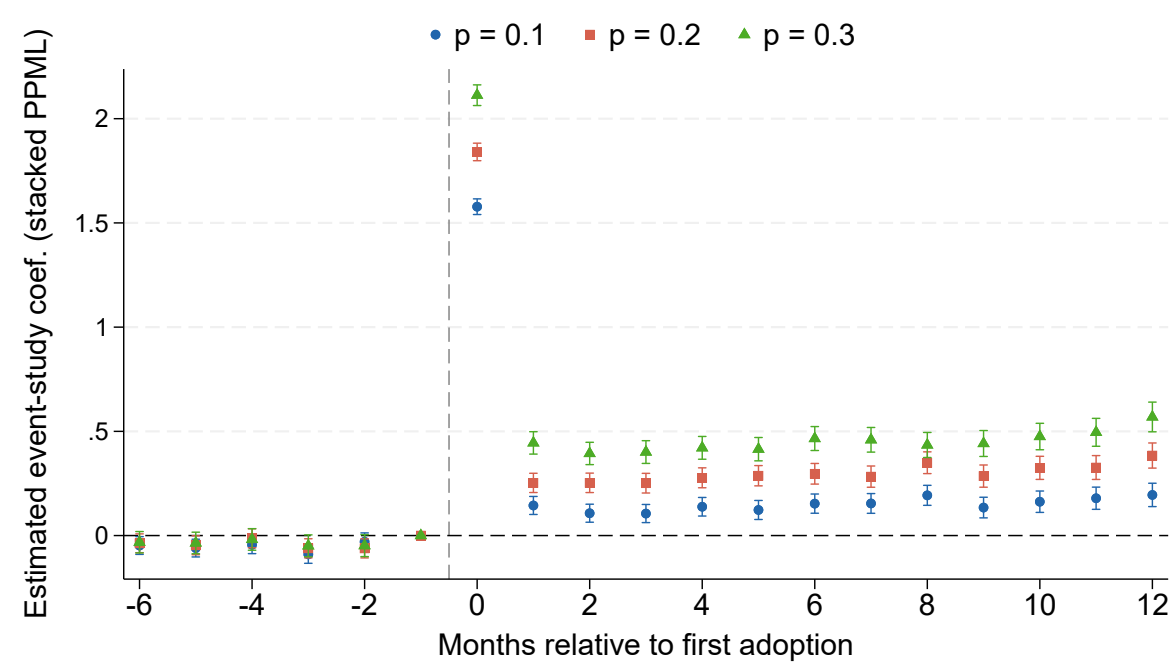

(D) **Balanded window:** $[-6; +12]$

Figure S33: **Random placebo event studies.** All panels report stacked-PPML event-study coefficients, with 95% confidence intervals, of the random placebo exercise, with different event-time balanced windows.

10.5 Stacked-PPML event study with alternative control group timing

Figure S34 reports stacked-PPML event-study estimates when applying alternative methods for defining the timing of the control group in the stacked design. Rather than assigning control authors a pseudo-treatment date equal to the treated cohort date, each control author is assigned a pseudo-date drawn from the empirical distribution of treated authors' adoption dates.

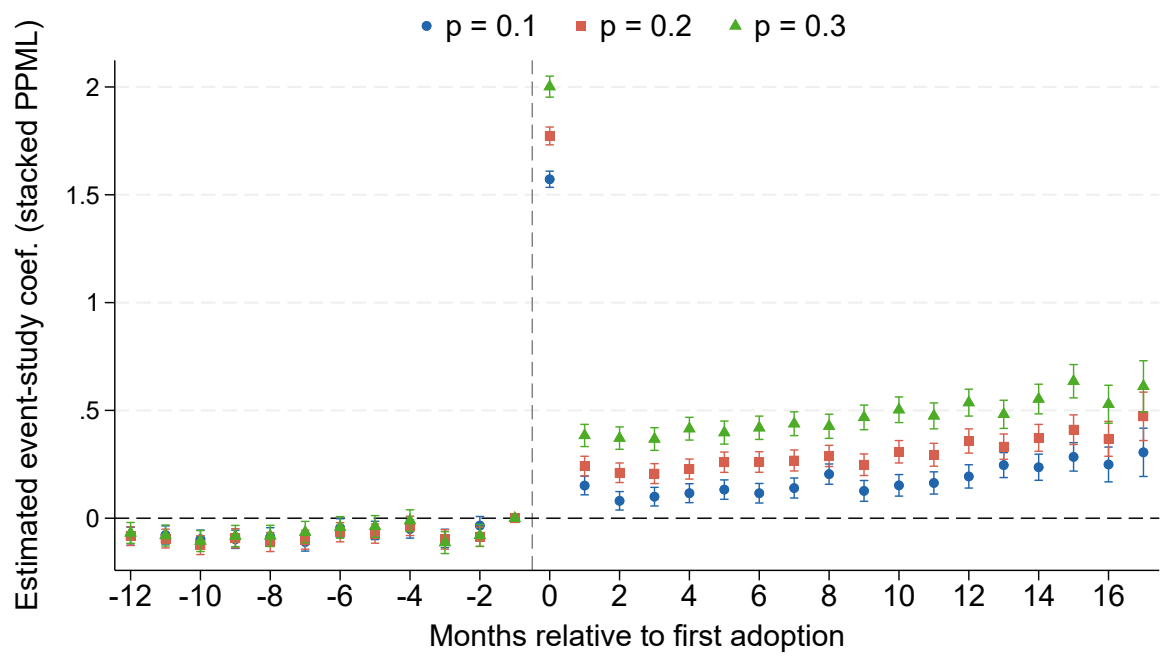

(A) **Placebo 1: random treatment**

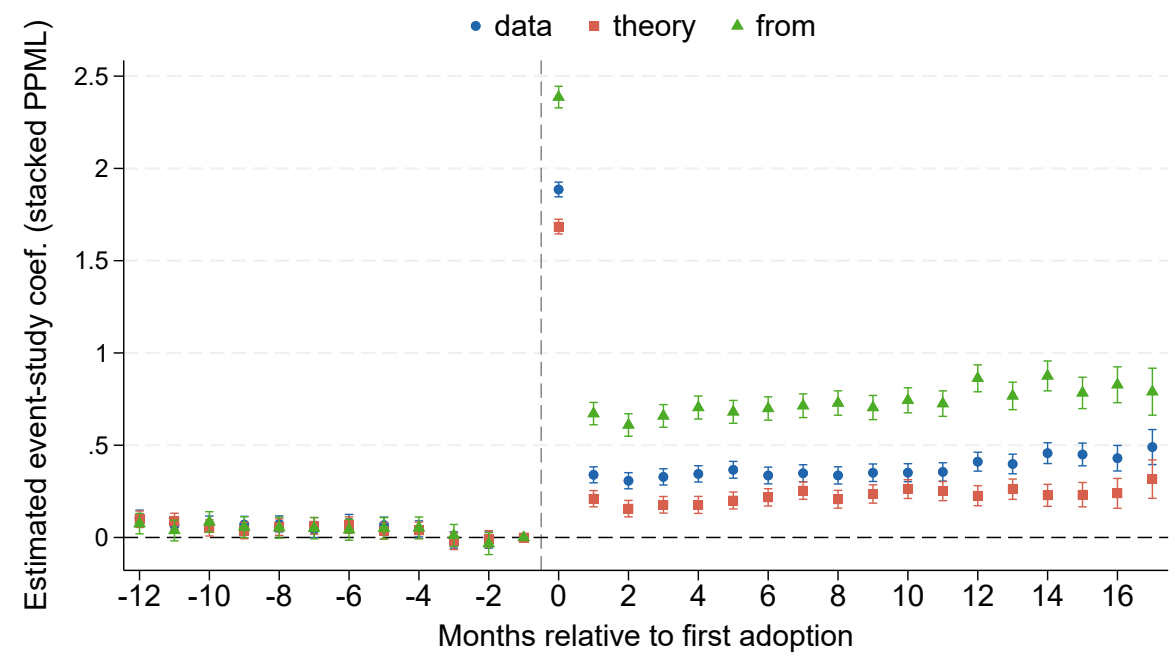

(B) **Placebo 2: keyword treatment**

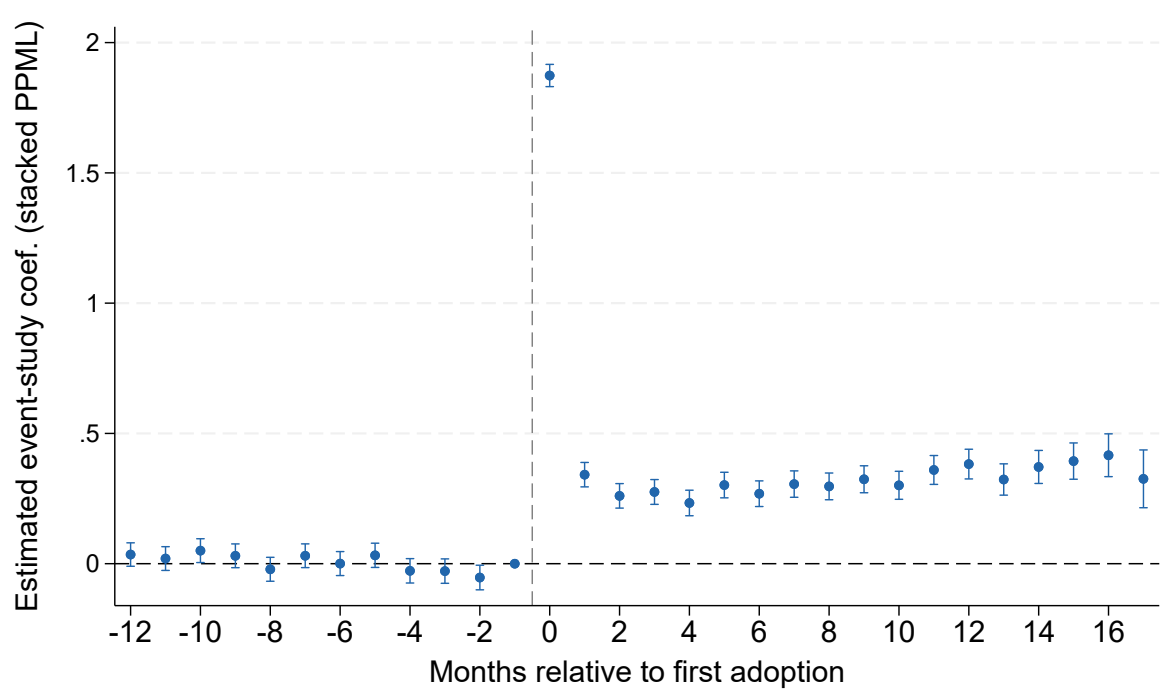

(C) **Placebo 3: pre-ChatGPT period**

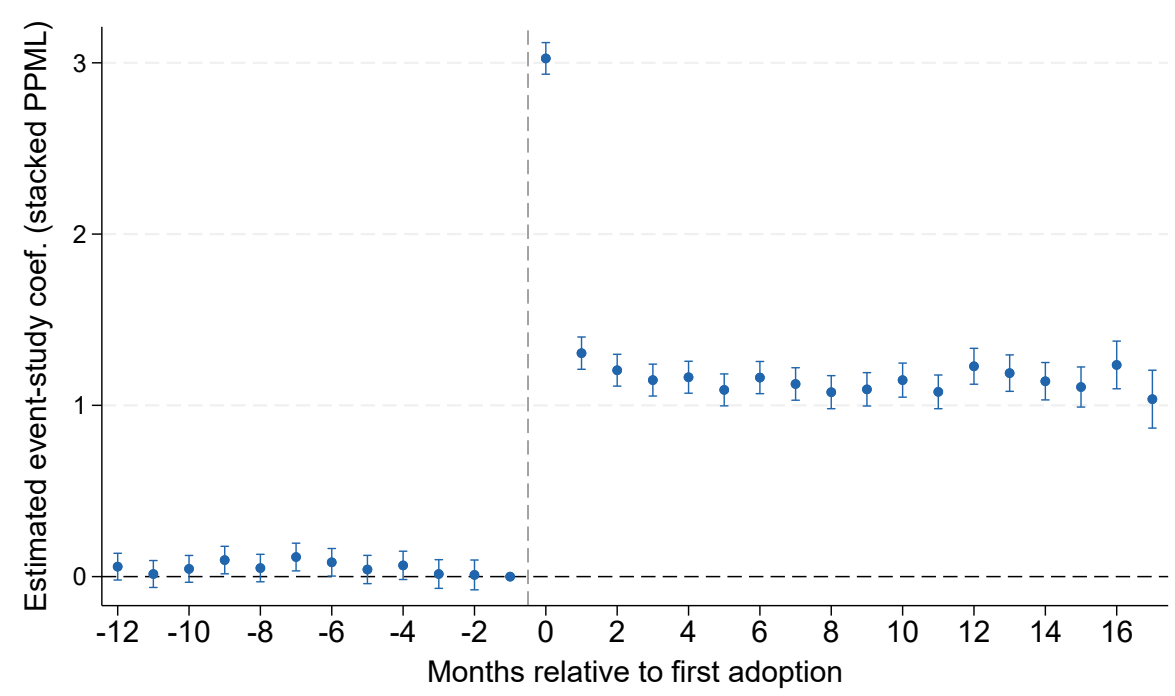

(D) **Placebo 4: inverted treatment**

Figure S34: **Placebo event studies.** All panels report stacked-PPML event-study coefficients, with 95% confidence intervals, of placebo exercises that replace the LLM detector with random paper-level flags (A), neutral keyword flags (B), an earlier observation window (C), and inverted treatment using $\alpha < 0.1$ instead of $\alpha > 0.1$ (D).

11 Materials and Methods: Additional Details

Panel construction: We use arXiv metadata from the Cornell/arXiv Kaggle snapshot covering January 2016 to June 2024. Following the sample construction of Kusumegi et al. [1], we exclude papers from core AI fields, including computer vision, machine learning, artificial intelligence, information retrieval, and computational linguistics. The main analysis focuses on active researchers with at least four publications between 2018 and 2021 and uses a January 2022 to June 2024 observation window. For the pre-ChatGPT placebo exercise, the activity filter is shifted backward to 2016-2019 and the observation window to January 2020-June 2022.

LLM detection model: To approximate the classification procedure used in Kusumegi et al. [1], and following Liang et al. [4], we implement a mixture-style detector trained on 20,000 synthetic abstract pairs composed of original abstracts and GPT-3.5 rewrites. We compute word frequencies separately in the human and rewritten corpora and derive word-level likelihood ratios from the difference between the two distributions. For each abstract, sentence-level scores are aggregated and the implied mixture share is estimated numerically. Papers are classified as LLM-assisted when the estimated mixture share exceeds 0.1.

Placebo tests: All placebo exercises preserve the same treatment-timing structure while removing any meaningful information about LLM adoption.

- Random assignment: Each paper submitted after December 2022 is independently assigned a random flag with probability p, irrespective of abstract content.
- Neutral keywords - Papers are flagged when their abstracts contain neutral words such as *data*, *theory*, or *from*.
- Pre-ChatGPT period - The entire empirical design is shifted to the January 2020 to June 2022 period, before the release of ChatGPT, using December 2020 as a placebo introduction date.
- Inverted treatment - Papers are flagged when the mixture-style detector is lower than 0.1, instead of greater than 0.1 in the original paper.

Stacked difference-in-differences design: Cohorts are defined by the calendar month of first detection under each treatment rule. Never-treated researchers are assigned placebo treatment dates drawn uniformly from the post-introduction period. For each cohort, treated researchers are stacked together with the corresponding never-treated controls. We estimate Poisson pseudo-maximum-likelihood (PPML) event-study regressions with researcher fixed effects and calendar-month-by-cohort fixed effects, clustering standard errors at the researcher level. The omitted reference period is the month immediately preceding treatment ($k = -1$). For consistency with the original analysis, the treatment-month indicator ($k = 0$) is omitted from the stacked 2×2 specification.

Simulation design: Our simulation generates a 30-month panel of author-level publication counts for 10,000 researchers. Monthly productivity is independently drawn from a Poisson distribution with an author-specific mean, so output is independent over time by construction. Starting in month 13 (this mirrors the original paper, where the sample starts in January 2022, but treatment cannot occur before January 2023 because ChatGPT was released only then), each simulated manuscript independently receives a flag with probability $p = 0.3$. We compare two treatment-timing rules using the same stacked PPML event-study specification: first-detection timing, in which a researcher becomes treated in the first month with at least one flagged paper, and random treatment timing assigned independently of output.

References

[1] Keigo Kusumegi et al. "Scientific production in the era of large language models". In: *Science* 390.6779 (2025), pp. 1240–1243.

[2] Clément de Chaisemartin and Xavier d'Haultfœuille. "Difference-in-Differences Estimators of Intertemporal Treatment Effects". In: *The Review of Economics and Statistics* (2026), pp. 1–18.

[3] Liyang Sun and Sarah Abraham. "Estimating dynamic treatment effects in event studies with heterogeneous treatment effects". In: *Journal of Econometrics* 225.2 (2021), pp. 175–199.

[4] Weixin Liang et al. "Quantifying large language model usage in scientific papers". In: *Nature Human Behaviour* (2025), pp. 1–11.
